# Integrating microRNA and mRNA expression profiling in *Symbiodinium microadriaticum*, a dinoflagellate symbiont of reef-building corals

**DOI:** 10.1186/1471-2164-14-704

**Published:** 2013-10-12

**Authors:** Sebastian Baumgarten, Till Bayer, Manuel Aranda, Yi Jin Liew, Adrian Carr, Gos Micklem, Christian R Voolstra

**Affiliations:** 1 Red Sea Research Center, King Abdullah University of Science and Technology (KAUST), 4700 KAUST, Thuwal 23955, Saudi Arabia; 2 Cambridge Systems Biology Centre & Department of Genetics, University of Cambridge, Cambridge, UK

**Keywords:** *Symbiodinium*, Dinoflagellates, Scleractinian corals, Symbiont, Coral reef, Small RNA (smRNA), microRNA (miRNA), Small interfering RNA (siRNA), mRNA, Expression profiling, RNAseq

## Abstract

**Background:**

Animal and plant genomes produce numerous small RNAs (smRNAs) that regulate gene expression post-transcriptionally affecting metabolism, development, and epigenetic inheritance. In order to characterize the repertoire of endogenous smRNAs and potential gene targets in dinoflagellates, we conducted smRNA and mRNA expression profiling over 9 experimental treatments of cultures from *Symbiodinium microadriaticum*, a photosynthetic symbiont of scleractinian corals.

**Results:**

We identified a set of 21 novel smRNAs that share stringent key features with functional microRNAs from other model organisms. smRNAs were predicted independently over all 9 treatments and their putative gene targets were identified. We found 1,720 animal-like target sites in the 3'UTRs of 12,858 mRNAs and 19 plant-like target sites in 51,917 genes. We assembled a transcriptome of 58,649 genes and determined differentially expressed genes (DEGs) between treatments. Heat stress was found to produce a much larger number of DEGs than other treatments that yielded only few DEGs. Analysis of DEGs also revealed that minicircle-encoded photosynthesis proteins seem to be common targets of transcriptional regulation. Furthermore, we identified the core RNAi protein machinery in *Symbiodinium*.

**Conclusions:**

Integration of smRNA and mRNA expression profiling identified a variety of processes that could be under microRNA control, e.g. protein modification, signaling, gene expression, and response to DNA damage. Given that *Symbiodinium* seems to have a paucity of transcription factors and differentially expressed genes, identification and characterization of its smRNA repertoire establishes the possibility of a range of gene regulatory mechanisms in dinoflagellates acting post-transcriptionally.

## Background

Only recently it has been shown that animal and plant genomes produce numerous small, noncoding RNAs that act as a guide for the Argonaute effector protein regulating gene expression and affecting processes of metabolism, development, epigenetic inheritance, and others [1-4]. Three classes of small RNAs (smRNAs) have been described, microRNAs (miRNAs), small interfering RNAs (siRNAs), and Piwi-interacting RNAs (piRNAs) [5]. miRNAs are the most common and best understood class of non-coding RNAs, but with ongoing research in the field of RNAi, differences and similarities in biogenesis and functionality of the different smRNA classes are becoming clearer [6]. miRNAs are ~22 nt small non-coding RNAs implicated in the regulation of gene expression in development and cell differentiation, the immune system, and homeostasis [7,8]. Homologous binding of a miRNA to its target genes leads to mRNA degradation and translational inhibition but also induces DNA methylation [9-14].

miRNAs are assumed to occur at a frequency of approximately 1% - 2% of the total number of genes in the genome of an organism [15]. Furthermore, it is estimated that about 20% to 30% of human genes are targeted by miRNAs as indicated by conserved seed pairing, often flanked by adenosines [16]. After the discovery of the first miRNAs in *Caenorhabditis elegans*, sequencing surveys have identified miRNAs in more than 100 organisms including those at the base of the metazoan tree [17]. Only recently, miRNAs have been shown to be expressed in unicellular eukaryotes and algae, e.g. *Chlamydomonas reinhardtii* and *Ectocarpus siliculosus.* Accordingly, it has been suggested that miRNAs have a long evolutionary history among eukaryotes [18]. However, a recent study by Tarver et al. [19] that proposed a number of criteria to unambiguously identify miRNAs (e.g. presence of miRNA and miRNA*, non-repetitive match to the genome, miRNA and miRNA* form a 2 nt overhang on the 3′ ends of the duplex) showed that the majority of identified miRNA types from unicellular protists might be explained by alternative means. The authors consequently stated that while the RNAi core molecular pathway and genes are conserved among eukaryotes (e.g. Dicer and Argonaute proteins), the products they produce are not, and hence RNAi might be an example of molecular exaptation [19].

Dinoflagellates are typically unicellular, photosynthetic, free-swimming, biflagellate organisms. They are important primary producers and constitute an important component of freshwater and marine phytoplanktonic communities. There are currently ~2,000 living species of dinoflagellates known, which are classified in ~125 genera. Dinoflagellates form one of the three main phyla of the alveolates (together with the ciliates and apicomplexans) [20]. About half of all dinoflagellates are autotrophic (photosynthetic), some are heterotrophic, saprophytic, symbiotic, or even parasitic. The autotrophic dinoflagellates are either free-living, or associated with a broad range of hosts as endosymbionts. Dinoflagellates possess unique molecular traits that differ from ‘classical’ model organisms. For instance, dinoflagellates have permanently condensed chromosomes [21-23] and DNA that contains some 5-hydroxymethyluracil in place of thymine [23]. Furthermore, dinoflagellates seem to harbor unusual genes and gene arrangements, such as unidirectional orientation of genes in the genome [24], bacterial type II RUBISCO [25], and minicircular plastid DNA [26]. Recent transcriptome studies in dinoflagellates show that dinoflagellates have a paucity of common transcription factors, and seem to only regulate few genes at the level of transcription [21,22,27-29].

One of the most successful mutualistic associations of dinoflagellates is found with scleractinian corals, which contain members of the genus *Symbiodinium* as endosymbiotic algae. This endosymbiotic relationship provides the foundation of coral reef ecosystems by providing the energy to construct the three-dimensional framework of coral reefs [30]. Together with a specific assemblage of bacteria (among other organisms) the coral host and dinoflagellate symbiont constitute the so-called coral holobiont [31]. While coral reefs form biodiversity hotspots in the oceans, their presence is declining because of local (e.g. overfishing, eutrophication, tourism) and global (e.g. ocean acidification and warming) impacts [32]. In order to characterize the molecular mechanisms driving these processes, understanding the contribution of each of the holobiont members to coral functioning is crucial. So far, researchers have conducted gene expression analyses mainly in the coral host [33-39] and looked at changes in the microbial community [40,41], while large scale gene expression studies in *Symbiodinium* are lacking. Given the apparent paucity of regulation of gene expression in *Symbiodinium* and dinoflagellates, a study investigating the integrated expression of smRNAs and mRNAs presents a compelling possibility to determine the presence of RNAi-related regulatory mechanisms that act post-transcriptionally, and provide an alternative means of regulating gene expression.

In this study, we conducted a comprehensive smRNA and mRNA expression-profiling screen in the dinoflagellate *Symbiodinium microadriaticum* (clade A1, strain CCMP2467, strain synonym 370, National Center for Marine Algae and Microbiota), which is a photosynthetic symbiont of scleractinian corals. We sequenced and analyzed 9 different experimental treatments of a cultured strain via Illumina single and paired-end sequencing. We were interested in 1) understanding presence, diversity, and expression of smRNAs and mRNAs, 2) identifying proteins of the RNAi machinery, and 3) integrating smRNA and mRNA expression in order to identify functional links between genes and potential smRNA regulators.

## Results

### smRNA diversity in *Symbiodinium microadriaticum*

**Figure 1:**
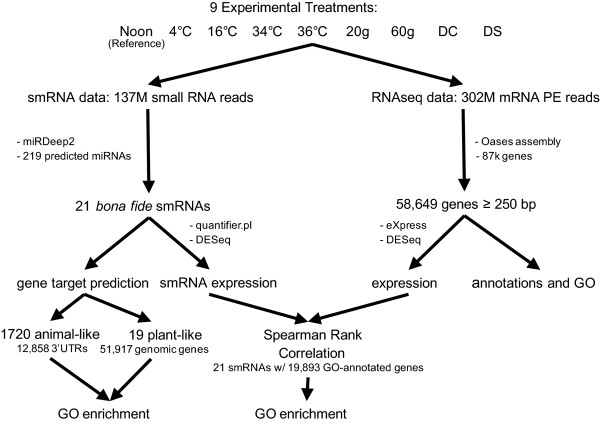
**Overview of smRNA and mRNA analysis workflow.** Cultures of *Symbiodinium microadriaticum* were subjected to 9 experimental treatments (noon: 12 h/12 h day/night cycle, sampled at noon; 4°C: 4°C for 4 hours; 16°C: 16°C for 4 hours; 34°C: 34°C for 12 hours; 36°C: 36°C for 4 hours; 20 g: 20 g/L NaCl salt content for 4 hours; 60 g: 60 g/L NaCl salt content for 4 hours; DS (dark stress): 18 hour dark period; DC (dark cycle): 12 h/12 h day/night cycle, sampled at midnight). Noon was selected as the reference condition for differential expression analyses. A total of 137 million small RNA reads resulted in the prediction of 219 miRNAs in 9 experimental treatments with the software miRDeep2, yielding a set of 21 smRNAs after further quality filtering. miRNA target gene prediction yielded 1,720 animal- and 19 plant-like miRNA binding sites via bowtie software in the set of 12,858 3'UTRs and 51,917 genes, respectively. Annotated miRNA targets were subsequently tested for GO category enrichment. A total of 302 million paired-end (PE) reads were assembled to a final gene set of 58,649 genes ≥ 250 bp with the Oases software. smRNA and mRNA expression over 9 experimental treatments was quantified with the DESeq software. Expression estimates of 21 smRNAs and 19,893 GO-annotated genes were assessed for correlation over 9 experimental treatments, and smRNAs-mRNA expression pairs displaying a correlation > 0.8 or < −0.8 (Spearman Rank) were tested for GO category enrichment.

**Figure 2:**
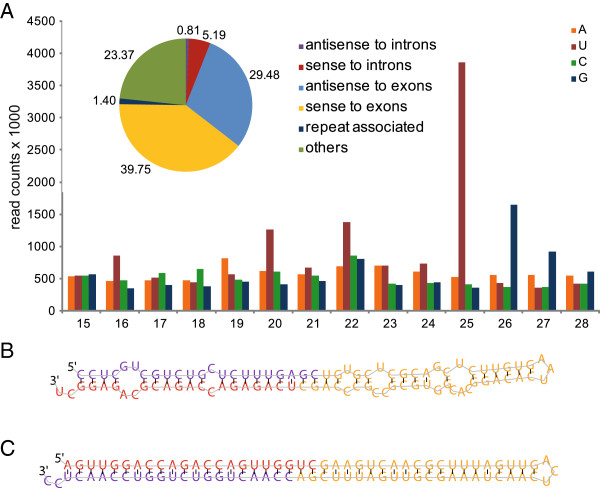
**smRNA identification in *Symbiodinium microadriaticum *over 9 experimental treatments. (A)** Lengths, read count distribution, and 5' identity of 5,125,940 distinct genome-matching small RNAs from the set of 137 million sequenced reads. All small RNA reads were mapped to a draft genome assembly via bowtie software. Genomic location is indicated in the pie chart. **(B)** miRDeep2 output for a miRNA precursor indicating the guide (red) and passenger (blue) strand as well as the hairpin loop (yellow). **(C)** miRDeep2 output for a siRNA precursor. Note the perfect complementarity between guide (red) and passenger (blue) strand as well as the hairpin loop (yellow).

A total of 137 million small RNA reads were sequenced over 9 experimental treatments (Table 1, Figure 1). After quality filtering and adapter trimming, 103 million high-quality reads were retained. Subsequent filtering of assembled small RNA contigs matching either the *Symbiodinium* transcriptome or known non-coding RNAs such as rRNAs, tRNAs, and snoRNAs removed an additional 3,743,490 (3.65%) reads. The remaining 99 million small RNA reads collapsed to 5,125,940 distinct genome-matching small RNA sequences in a size range from 15 – 28 nt with the highest read counts falling into the 25 nt size fraction, followed by the 22 nt fraction (Figure 2A). Both size fractions were strongly biased towards a 5′-uridine identity (Figure 2A). More than two-thirds of the small RNAs could be mapped either antisense (29.48%) or sense (39.75%) to exons, only a small portion were found to be repeat-associated (1.40%), or in sense (5.19%) or antisense (0.81%) direction to introns. 23.37% of smRNA reads were mapped to other genomic locations.

**Table 1:** Overview over smRNA and mRNA sequencing and assembly statistics

**Library name**	**4°C**	**16°C**	**34°C**	**36°C**	**20 g**	**60 g**	**DC**	**DS**	**Noon**
**Experimental treatment**	**[4°C 4hs]**	**[16°C 4hs]**	**[34°C 12hs]**	**[36°C 4hs]**	**[20 g/L NaCl 4hs]**	**[60 g/L NaCl 4hs]**	**[midnight]**	**[24hs dark]**	**[reference]**
***smRNA***									
Total base pairs	476,999,446	327,143,288	185,735,770	254,824,059	427,785,834	220,539,256	258,016,695	133,416,325	361,512,933
No. of reads (after trimming)	17,193,220	12,593,192	8,433,048	10,658,877	15,378,342	8,725,667	10,662,995	6,131,326	13,591,577
Mean read length	28	26	22	24	28	25	24	22	27
No. of reads (after smRNA filtering)	16,404,546	12,102,929	8,234,625	10,180,915	14,747,100	8,399,643	10,259,502	6,025,546	13,017,083
No. of unique reads	2,288,514	2,198,132	1,633,046	2,338,445	2,352,632	1,705,659	1,964,913	1,405,173	1,881,585
No. of miRNAs (miRDeep2)	118	96	131	114	136	104	129	115	138
Mean readcount of miRNAs	149	418	300	199	257	170	253	171	446
No. of miRNAs w/ miRNA* (miRDeep2)	55	83	84	66	61	70	77	75	81
No. of miRNAs (after manual inspection)	21	21	21	21	21	21	21	21	21
***mRNA***									
No. of read pairs (2 x 100 bp)	26,119,370	34,506,164	41,918,850	30,712,175	39,211,121	34,918,126	28,787,997	36,782,164	29,985,278
No. read pairs mapped	16,777,195	23,084,001	27,761,160	19,905,837	24,920,330	23,277,719	19,357,409	24,141,222	20,023,519
No. of 58,649 genes expressed (FPKM > 0)	55,881	56,353	57,389	55,518	55,287	56,526	56,292	56,041	56,778
No. of 58,649 genes expressed (FPKM > 5)	40,078	39,125	39,246	37,256	37,435	39,198	38,041	38,422	40,206
Median FPKM	12.58	12.29	11.87	11.42	11.13	12.38	11.41	11.66	12.49
BLASTX annotation [%]	44.81	44.51	43.80	45.10	45.39	44.44	44.59	44.81	44.18
Pfam [%]	34.50	34.23	33.63	34.71	34.87	34.13	34.27	34.43	33.98
GO annotation [%]	34.90	34.67	34.08	35.14	35.32	34.59	34.71	34.89	34.39
DEGs all (vs. noon)	119	37	351	2,465	138	48	67	60	-
DEGs up (vs. noon)	47	12	105	293	21	14	22	28	-
DEGs down (vs. noon)	72	25	246	2,172	117	34	45	32	-
log_2_ difference (minimum/maximum)	−8.30/+6.61	−7.27/+5.27	−6.84/+7.34	−8.43/+8.17	−10.43/+5.46	−7.02/+6.24	−7.32/+5.19	−7.06/+7.64	-
log_2_ difference (mean)	2.93	3.15	2.29	2.79	2.73	3.27	2.99	3.01	-

DEG = Differentially Expressed Gene.

miRDeep2 [42] predicted 219 non-redundant and so far unknown miRNAs. From this set, we identified 8 novel miRNAs (Figure 2B, Table 2, Additional file 1) that fulfilled all criteria for miRNA identification from higher eukaryotes (see Material and Methods). Another 13 miRNA candidates fulfilled these criteria but additionally featured perfect base pair complementarity of the passenger/guide duplex (Figure 2C, Table 2, Additional file 1). This feature is known from endogenous small-interfering RNAs (siRNAs) that are specifically cleaved from dsRNA [6]. This set of 21 *bona fide* smRNAs was not significantly differentially expressed in pairwise comparisons of treatments to the selected reference condition noon (DESeq, FDR < 0.1).

**Table 2:** Set of 21 smRNAs (8 miRNAs and 13 siRNAs) that were independently identified over 9 experimental treatments and matched all criteria for smRNA identification

**miRNA**	**Sequence**	**Length**	**Stem-loop length**	**MFE [kcalmol-1]**	**Read count mature strand**	**Read count star strand**
smb107.2	CAAGGAUGGGAUGCUCAGAGAA	22	88	−69.3	13,315	398 (9)
smb107.3	CAAGGAUGGGAUGGUCAGAGAA	22	88	−64.1	3,322	398 (9)
smb123	CAGUCGGCCAAAGUGCUGGACC	22	89	−64.1	451	154 (9)
smb203	CUUUGUAUCCCGGAUCCUGAUA	22	87	−46.6	1,087	389 (9)
smb215	GAGGAUGCUGAUCAUUCACUGG	22	87	−80.6	85	34 (8)
smb295	UCAGAGACCAGACGCAGAGGCU	22	90	−40.6	12,543	160 (9)
smb297	UCAGUGGCAGAAGCUGGGAACU	22	87	−63.5	965	60 (8)
smb313	UCGAACUUUCAGGAAUAGUAUC	22	87	−54.8	2,707	1475 (9)
**siRNA**	**Sequence**	**Length**	**Stem-loop length**	**MFE [kcalmol-1]**	**Read count mature strand**	**Read count star strand**
smb21	AAUUUGAACGUUGCCAUCUAUC	22	87	−72.5	123	9 (7)
smb41	ACCUGCAGCAUUUGGCGCCUGA	22	84	−77.9	299	18 (7)
smb51	ACUUAGAACUCUCCUACGAGGG	22	88	−83.1	510	275 (8)
smb79	AGUUGGACCAGACCAGUUGGUC	22	87	−71.7	489	319 (9)
smb83	AUCACUCCACAAAGGGAUUUG	21	87	−65.5	217	7 (5)
smb101	CAACGAGAUUGGCCUUCUGUGC	22	87	−82.9	5,234	412 (9)
smb163	CGGGACUCGAUUCGGAGGGUGC	22	88	−63.6	2,015	660 (9)
smb271	UAGAAUGUAGUCGUCAUCUUGC	22	88	−68.9	1,044	29 (9)
smb303	UCCGCCGUGCAACUGUCGCAAC	22	88	−80.2	207	107 (9)
smb359	UGAUGUACAUCGAUUGAUCGAC	22	86	−63.8	646	23 (9)
smb365	UGCCAACGUGAUUUGCAACUCC	22	84	−68.1	333	75 (7)
smb379	UGGACUUGGAAAGCUUCUCUGC	22	86	−76.7	2,505	2 (2)
smb427	UUUGUCCAGUGUACCUGCGCU	21	85	−73	728	50 (8)

Read counts for guide and passenger strands were derived by pooling counts over conditions. The number of experimental treatments that identified the passenger strand is indicated in brackets. MFE = Minimum Free Energy of precursor.

The majority of guide smRNAs were predicted in 9 conditions (n = 12) and another 9 smRNAs were predicted in 8 (n = 8) and 7 (n = 1) conditions, respectively. For most guide smRNA sequences (n = 19), the respective passenger smRNA sequence was found in at least 7 conditions, and only for 2 smRNAs the corresponding passenger smRNAs were only found in 5 and 2 libraries (Table 2). The lengths of the 21 smRNAs varied between 21 nt an 22 nt, but the majority had a length of 22 nt (n = 19) with a bias towards uridine as the 5′ nucleotide (n = 9) (Table 2). This provides support to the presence of miRNA functionality in *Symbiodinium* as Mi et al. [43] have shown that loading of small RNAs in Argonaute proteins in *Arabidopsis* is directed and critically dependent on a 5′ terminal uridine. The lengths of the corresponding smRNA precursors (i.e. guide strand, passenger strand, and hairpin loop) varied from 84 nt to 90 nt, and guide strands were found to be processed from the 5′ and 3′ end of the fold-back. 8 of the 21 smRNA precursors could be mapped to either intronic regions (n = 3) or to unannotated transcripts (n = 5), both regions have been described to encode precursor miRNAs [44]. Minimum predicted free energies ranged from −40.6 kcalmol^-1^ to −83.1 kcalmol^-1^ with an average of −68.2 kcalmol^-1^ for the fold back (Table 2). This is in line with values of validated pre-miRNAs from other studies. For instance, estimated values for wheat averaged at −72.4 kcalmol^-1^[45]. It is important to note that sufficiently low fold back energies for miRNA annotations can also be attained by complementary pairing outside of the duplex region, while the miRNA guide-passenger duplex itself features mismatch base pairing. Accordingly, the number of matching base pairs between the mature and star sequences, and not the energies themselves, are the critical aspect.

### smRNA target genes in *Symbiodinium microadriaticum*

smRNA-dependent post-transcriptional regulation works through binding of smRNAs to specific complementary target sites within transcripts, which ultimately results in gene silencing. The composition of target sites is different for animals and plants. smRNA target sites in animals are characterized by short complementary regions in the UTR of a gene giving rise to mismatches and bulges, but a general feature is Watson-Crick base pairing of miRNA nucleotides 2–8 in three canonical manners (i.e. 7mer-m8, 7mer-A1, 8mer) [15]. In contrast, plant miRNAs bind over their entire length (with only few bp mismatches) to the coding sequence and/or the UTR of a gene (i.e. near-perfect complementarity). Given that *Symbiodinium* diverged between 1,300 and 1,800 million years ago from the last common ancestor of eukaryotes [46-48], and therefore shares a similar evolutionary distance to plant and animals [49], we searched for both animal- and plant-like target genes.

**Figure 3:**
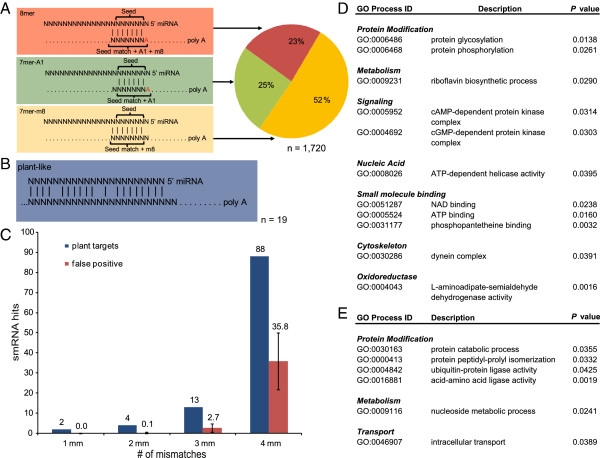
**smRNA target prediction for 21 smRNAs within the gene set of *Symbiodinium microadriaticum*. (A)** 3 distinct animal-like target sites in the 3'UTR of genes exist that are characterized by seeds of lengths 6-8nt that display perfect complementary base pairing between the miRNA and mRNA sequence. Vertical dashes indicate Watson-Crick base pairing. The pie chart displays the relative frequency of these target sites in the 3'UTRs of 1,720 genes (from a set of 12,858 genes with available 3'UTRs). **(B)** 19 plant-like mRNA target sites were identified in the coding sequence and 3'UTRs of 51,917 genomic genes of *Symbiodinium microadriaticum*. Plant-like mRNA target sites are characterized by full-length complementary base pairing between a miRNA and its mRNA with only few mismatches (i.e. near-perfect). **(C)** Number of identified plant-like mRNA target sites (blue bars) in relation to number of mismatches allowed. Number of false positives in 1,000 randomly generated cohorts of small RNA sequences of length 22 nt (red bars) are displayed for comparison. A cutoff of 3 mismatches (mm) over the aligned smRNA and mRNA provides a False Positive Rate of about 1 in 5. **(D)** Enriched GO terms within the set of matching and annotated animal-like targets (n = 519, *P* < 0.05, 4,047 3' UTRs). **(E)** Enriched GO terms within the set of matching and annotated plant-like targets (n = 7, *P* < 0.05, 18,290 genes).

In total, we found 1,720 animal-like target sites in the 3′ UTRs of 12,858 genes from the set of 51,917 genomic genes (Figure 3A, Additional file 2). Most target sites matched 7mer-m8 seeds (n = 878). The remaining 842 target sites were flanked by a 3′ adenosine in the mRNA (7mer-A1: n = 438; 8mer: n = 404). Previous studies showed that the 3′ adenosine anchor of miRNA targets is highly overrepresented for miRNAs of any 5′ identity, and accordingly, presents a feature that increases confidence in miRNA target predictions [16]. Most stable miRNA-mRNA duplexes were formed by 8mer (mean *ΔG*_Duplex_ = −23.82 kcalmol^-1^) and 7mer-m8 target sites (mean *ΔG*_Duplex_ = −23.77 kcalmol^-1^), followed by 7mer-A1 sites (mean *ΔG*_Duplex_ = −21.92 kcalmol^-1^). Taking into account the energy needed to open mRNA secondary structures (i.e. *ΔG*_Open_), overall energy requirements (i.e. *ΔΔG* = *ΔG*_Duplex_ – *ΔG*_Open_) averaged at −12.83 kcalmol^-1^ and only differed slightly with respect to target seeds (i.e. 8mer −13.27 kcalmol^-1^, 7mer-m8 -12.67 kcalmol^-1^, 7mer-A1 -12.56 kcalmol^-1^). 36 predicted target genes provided at least two copies of landing sites for a specific miRNA (mean *ΔΔG*_Score_ = −12.9 kcalmol^-1^).

We searched for plant-like target sites by looking for near-perfect base pairing between smRNAs and mRNAs in the set of 51,917 genomic genes (Figure 3B) [6]. 20 of the 21 smRNAs targeted a total of 107 genes with four or fewer mismatches over the entire lengths of the smRNAs. 100 of these showed complementarity to the predicted coding sequence (CDS), whereas for 7 genes smRNA binding sites were identified in the 3′ UTR. In order to control the rate of false positives, we conducted 1,000 identical searches with cohorts of 21 randomized small RNAs against the set of 51,917 genomic genes. This analysis showed that by decreasing the number of mismatches from 4 to 3, the ratio of false positives dropped from around 1:2.4 to 1:4.8 (Figure 3C). Adjusting smRNA-mRNA complementarity to a maximum of 3 mismatches resulted in 19 plant-like targets with high confidence. Of these targets, 16 showed complementarity within the predicted CDS and 3 were found in the respective 3′ UTRs (Additional file 3).

Next we analyzed the set of predicted and GO-annotated animal- and plant-like target genes for GO category overrepresentation via GOEAST [50] in order to identify molecular processes in which smRNAs are potentially involved. GO annotations for 519 animal-like miRNA target genes were tested for GO term enrichment against a background set of 4,047 GO-annotated genes (Fisher’s Exact Test, Adrian Alexa’s scoring algorithm, *P* < 0.05) (Figure 3D). Similarly, an enrichment analysis was conducted for 7 GO-annotated plant-like miRNA target genes that were searched against a background set of 18,290 GO-annotated genomic genes (Figure 3E). Both analyses showed an enrichment of processes related to protein modification and metabolism (among others). Additionally, we found processes related to signaling, nucleic acid, small molecule binding, cytoskeleton, and oxidoreductase to be enriched in the set of predicted animal-like target genes.

### *Symbiodinium microadriaticum* transcriptome and expression

A total of 302 million RNA read pairs were sequenced over all 9 libraries and assembled into 58,649 genes ≥ 250 bp with an average transcript length of 1,324 bp (Figure 1, Table 3). About 43% of the genes could be annotated via BLASTX to any of the Swiss-Prot, TrEMBL [51], or GenBank nr [52] databases with an e-value < 1e^-5^ (Table 3, Additional file 4). Between 26 and 42 million RNA read pairs were sequenced for each experimental treatment (Table 1), and around 65% of those could be mapped to the transcriptome for expression estimates (Additional file 5). Interestingly, we found between 94% and 98% of all genes expressed in any condition (i.e. Fragments Per Kilobase of transcript per Million mapped reads (FPKM) > 0). Taking into consideration only the genes with an FPKM > 5, we still found between 64% and 69% of the transcriptome to be expressed under any condition. The 9 conditions were equally well annotated and we did not find deviations in the distribution of gene annotations for the different treatments (Table 1). We found an underrepresentation of known transcription factor binding domains (617 domains in 610 genes, ~ 1% of 58,649 genes). The CCCH-type zinc finger domain (n = 147) and the cold-shock domain (n = 127) were the most prevalent. Genes bearing transcription factor domains were expressed between 0.22 and 777.43 FPKM (median expression over all experimental treatments), indicating a highly variant expression.

**Table 3:** **Summary of the *Symbiodinium microadriaticum *transcriptome assembly**

**No. of read pairs**	**302,941,245**	
No. of genes (≥ 250 bp)	58,649	
Mean transcript length bp	1,324	
BLASTX annotation	25,288	43.12%
Pfam annotation	16,446	28.04%
KEGG annotation	9,914	16.90%

For elucidation of differentially expressed genes, the experimental condition noon was selected as a reference to which the other treatments were compared (Table 1, Additional file 6). The 9 experimental treatments were: 1) noon: 12 h/12 h day/night cycle, sampled at noon; 2) 4°C: 4°C for 4 hours; 3) 16°C: 16°C for 4 hours; 4) 34°C: 34°C for 12 hours; 5) 36°C: 36°C for 4 hours; 6) 20 g: 20 g/L NaCl salt content for 4 hours; 7) 60 g: 60 g/L NaCl salt content for 4 hours; 8) DS (dark stress): 18 hour dark period; 9) DC (dark cycle): 12 h/12 h day/night cycle, sampled at midnight). In contrast to the large number of assembled genes in the transcriptome (n = 58,649), we found a very low number of significantly differentially expressed genes (Table 1), namely between 37 (16°C) and 138 genes (20 g), except for heat stress-related treatments. Incubation of cultures at 34°C for 12 hours resulted in differential expression of 351 genes, whereas exposure to 36°C for 4 hours resulted in 2,465 differentially expressed genes. In the 36°C treatment the number of downregulated genes (n = 2,172) exceeded the number of upregulated genes (n = 293) more than 7-fold. On average, we found a higher number of genes to be downregulated than upregulated (Table 1). This might be attributed to our choice of reference. The noon experimental treatment showed the highest number of expressed genes considering a FPKM > 5. Average fold-changes of differentially expressed genes were around 8-fold (i.e. log_2_ difference of ~3), and minimum and maximum fold-changes exceeded −1000-fold (20 g, *Locus_88253*: not annotated, log_2_ difference = −10.43) and +250-fold (36°C, *Locus_11567*: not annotated, log_2_ difference = +8.17), respectively (Additional file 6).

#### Differential expression of photosynthetic genes among experimental treatments

We looked for enrichment of GO categories in the set of significantly differentially expressed genes of a given treatment (Additional file 7). We found the highest number of significantly enriched categories (Fisher’s Exact Test, Adrian Alexa’s scoring algorithm, *P* < 0.05) in the 36°C treatment (n = 97) and the lowest number in the 16°C (n = 11), corresponding to the highest and lowest number of differentially expressed genes over treatments. Only less than half of all genes could be GO-annotated (19,893 of 58,649 genes).

In the 4°C treatment we found photosynthesis-related terms to be enriched (among others). Additionally, and similarly to the 16°C condition, we found carbon utilization as an affected process. This might be explained by the depletion of primary carbon sources due to decrease in photosynthetic productivity as indicated, e.g. by a ~20-fold downregulation of the gene carbonic anhydrase (*Locus_3248*, Additional file 6).

For both, the 34°C and 36°C treatment, we found motility- and membrane-related processes to be affected. However, while we saw upregulation of heat shock and oxidative stress related genes in the 34°C condition (e.g. *Locus_44661*: Chaperone protein DnaJ, *Locus_28763*: Heat shock protein DDB, *Locus_817*: Peroxidredoxin-5), the 36°C condition did not show upregulation of stress-related genes but rather was characterized by an overall downregulation of gene expression. This was substantiated in the GO process analysis where we saw a diversity of processes to be affected that were not necessarily related to a heat shock response (e.g. transmembrane transport, elastic fiber assembly, nitrate assimilation, etc.). Additionally, a suite of photosynthesis-related processes were identified, though primarily in the 36°C condition.

The analysis of treatments related to ionic stress (20 g and 60 g) both showed a consistent and broad downregulation of photosynthesis-related genes (e.g. *Locus_33090*: Photosystem II CP47 chlorophyll apoprotein, *Locus_27958*: Photosystem I P700 chlorophyll a apoprotein A1, *Locus_30419*: Photosystem I P700 chlorophyll a apoprotein A2) and processes (e.g. GO0015979: photosynthesis, GO0015986: ATP synthesis coupled proton transport, GO0009535: chloroplast thylakoid membrane). Overall, we identified a common set of 22 genes and 15 processes in both of these treatments. However, histone demethylation and histone demethylase activity were among the GO terms that were only enriched in the 20 g treatment. This might indicate that alteration of histone methylation states plays a role in ionic stress.

For the dark cycle (DC) and dark stress (DS) treatments we again saw a wide representation of GO processes related to photosynthesis. Overall, we identified a set of 11 out of 21 (DC) and 11 out of 34 (DS) GO terms that were significantly enriched in both treatments (e.g. GO0015986: ATP synthesis coupled proton transport, GO0009535: chloroplast thylakoid membrane, GO0009767: photosynthetic electron transport chain). Additionally, we identified terms related to oxidative stress (i.e. GO:0051920 peroxidredoxin activity, GO:0004601 peroxidase activity) but only in the DS treatment.

### *Symbiodinium microadriaticum* RNAi pathway

While the extent of evolutionary conservation of smRNAs in eukaryotes is controversial, all organisms seem to possess a shared and inherited RNAi machinery that consists in its core of the proteins Dicer (DIC) and Argonaute (AGO) [19]. We identified 1 Dicer and 3 Argonaute homologs in our genome and transcriptome data (Additional file 8, Additional file 9).

In the Dicer homolog, we found the two RNase III domains that occupy a central role in the cleavage of the guide-passenger duplex from its double-stranded precursor [53]. More specifically, we identified the key acidic residues that coordinate a divalent Mg^2+^ ion, which is essential for the activity of the ribonuclease, to be conserved in our homolog [54]. Additionally, we identified the conserved dsRBD domain, whereas a PAZ domain was not found. In contrast, the 3 *Symbiodinium* Argonaute homologs all displayed a PAZ domain as well as a Piwi domain. The PAZ domain binds to dsRNA ends, preferentially with short 3′ nt overhangs [55,56], and is shared between proteins of the Argonaute and Dicer family. Consequently the absence of a PAZ domain in Dicer might be related to the draft nature of the genome used. Overall, all Argonaute homologues displayed strong evolutionary conservation to model organisms as well as to each other. Last, we were interested in elucidating whether homologs of the small RNA 2′-O-methyltransferase (HEN1) existed. This protein is needed for final maturation of a subset of small RNAs (e.g. miRNAs and siRNAs in plants, piRNAs in animals, etc.) by 2′-O-methylation on the 3′ terminal nucleotide [57]. We found 1 homolog of the small RNA 2′-O-methyltransferase (HEN1) showing a high degree of conservation in the crucial methyltransferase domain (Additional file 10).

Despite the absence of the PAZ RNA binding domain in Dicer, conservation of the key protein domains in homologs of Dicer, Argonaute, and HEN1 suggest the presence of a functional RNAi machinery in *Symbiodinium*, and confirms the deep phylogenetic history of the miRNA protein machinery.

### Integrating smRNA and mRNA expression profiling

Previous studies have shown that integrating smRNA with mRNA expression data is able to uncover smRNA-mRNA gene regulatory network relationships [58,59]. Here, we correlated smRNA and mRNA expression estimates over all 9 treatments to identify processes that are under potential smRNA control. To do this, expression of our 21 identified smRNAs was correlated to 19,893 GO-annotated genes of the *Symbiodinium* transcriptome assembly, resulting in 417,753 comparisons. In total, 4,388 smRNA-mRNA comparisons had a correlation coefficient of *Rho* > +0.8 or < −0.8, representing 3,502 distinct genes (Table 4). The total number of negatively and positively correlated genes was similar, but we found a slightly higher number of negatively correlated genes (2,235 genes vs. 2,153 genes).

**Table 4:** Correlation between smRNAs and mRNAs (Spearman’s Rho > +0.8 or < −0.8) over 9 experimental treatments (21 smRNAs, 19,893 annotated mRNAs, 417,753 comparisons)

**smRNAs**	**No. of genes**		
	Negative correlation	Positive correlation	Total
smb123	235	363	598
smb83	237	172	409
smb359	134	188	322
smb21	116	196	312
smb107.3	208	91	299
smb303	149	138	287
smb101	189	76	265
smb107.2	157	106	263
smb215	104	108	212
smb297	86	113	199
smb427	114	74	188
smb79	92	69	161
smb51	70	85	155
smb295	101	39	140
smb163	47	71	118
smb365	43	51	94
smb271	31	60	91
smb203	41	35	76
smb313	17	50	67
smb379	30	36	66
smb41	34	32	66
Total no. genes	2,235	2,153	4,388
Distinct no. genes	1,673	1,602	3,502

Interestingly, the number of distinct (i.e. non-overlapping) genes was very similar to the total number of genes that were negatively or positively correlated. This indicates that relatively little overlap existed between correlated genes identified for the different smRNAs. The number of correlated genes for a given smRNA ranged from 66 to 598.

We searched for enriched functions in the set of correlated genes over all smRNAs (Table 5, Additional file 11). We identified 49 enriched GO terms over all smRNAs that were negatively correlated to mRNA expression (Fisher’s Exact Test, Adrian Alexa’s algorithm, *P* < 0.05). Similarly, we identified 60 enriched GO terms over all smRNAs that were positively correlated to mRNA expression profiles. Manual assortment of enriched GO terms to higher order categories revealed an overlap in processes for positively and negatively correlated smRNA-mRNA pairs (e.g. protein modification, signaling, gene expression, translation, and metabolism). Interestingly, we also found GO terms associated with immunity (e.g. GO:001644 somatic hypermutation of immunoglobulin genes, GO:0002698 negative regulation of immune effector process) and DNA damage (e.g. GO:0006307 DNA dealkylation involved in DNA repair, GO:0008630 intrinsic apoptotic signaling pathway in response to DNA damage).

**Table 5:** Enrichment of GO terms of negatively and positively correlated smRNA-mRNA expression pairs to manually assorted higher order categories

**GO process ID**	**Description**	***P***
**Protein modification**		
*Negative correlation*		
GO:0006457	Protein folding	0.000
GO:0051082	Unfolded protein binding	0.002
GO:0004252	Serine-type endopeptidase activity	0.013
GO:0018106	Peptidyl-histidine phosphorylation	0.009
GO:0016925	Protein sumoylation	0.005
GO:0003755	Peptidyl-prolyl cis-trans isomerase activity	0.025
GO:0036065	Fucosylation	0.034
GO:0000413	Protein peptidyl-prolyl isomerization	0.021
*Positive correlation*		
GO:0019787	Small conjugating protein ligase activity	0.021
GO:0006487	Protein N-linked glycosylation	0.045
GO:0047485	Protein N-terminus binding	0.013
GO:0070534	Protein K63-linked ubiquitination	0.011
GO:0042787	Protein ubiquitination in ubiquitin-dependent protein catabolic process	0.021
GO:0019773	Proteasome core complex, alpha-subunit complex	0.034
GO:0004180	Carboxypeptidase activity	0.000
**Immunity**		
*Negative correlation*		
GO:0016446	Somatic hypermutation of immunoglobulin genes	0.009
*Positive correlation*		
GO:0002666	Positive regulation of T cell tolerance induction	0.015
GO:0042130	Negative regulation of T cell proliferation	0.024
GO:0002698	Negative regulation of immune effector process	0.036
**Signaling**		
*Negative correlation*		
GO:0023014	Signal transduction by phosphorylation	0.046
GO:0009909	Regulation of flower development	0.034
GO:0010019	Chloroplast-nucleus signaling pathway	0.011
GO:0031930	Mitochondria-nucleus signaling pathway	0.011
*Positive correlation*		
GO:0031930	Mitochondria-nucleus signaling pathway	0.000
**DNA damage**		
*Negative correlation*		
GO:0032404	Mismatch repair complex binding	0.001
GO:0006307	DNA dealkylation involved in DNA repair	0.034
GO:0032300	Mismatch repair complex	0.005
GO:0031072	Heat shock protein binding	0.005
GO:0008630	Intrinsic apoptotic signaling pathway in response to DNA damage	0.038
*Positive correlation*		
GO:0008630	Intrinsic apoptotic signaling pathway in response to DNA damage	0.036
GO:0006289	Nucleotide-excision repair	0.015
**Gene expression**		
*Negative correlation*		
GO:0035552	Oxidative single-stranded DNA demethylation	0.028
GO:0030261	Chromosome condensation	0.004
*Positive correlation*		
GO:0031048	Chromatin silencing by small RNA	0.008
**Translation**		
*Negative correlation*		
GO:0006412	Translation	0.030
GO:0003735	Structural constituent of ribosome	0.006
GO:0022627	Cytosolic small ribosomal subunit	0.009
GO:0042255	Ribosome assembly	0.013
GO:0016071	mRNA metabolic process	0.050
GO:0006364	rRNA processing	0.000
*Positive correlation*		
GO:0008353	RNA polymerase II carboxy-terminal domain kinase activity	0.032
GO:0005689	U12-type spliceosomal complex	0.008
GO:0015935	Small ribosomal subunit	0.000
**Metabolism**		
*Negative correlation*		
GO:0043734	DNA-N1-methyladenine dioxygenase activity	0.030
GO:0051747	Cytosine C-5 DNA demethylase activity	0.030
GO:0043462	Regulation of ATPase activity	0.017
GO:0019213	Deacetylase activity	0.017
GO:0016811	Hydrolase activity	0.045
GO:0019478	D-amino acid catabolic process	0.001
GO:0016308	1-phosphatidylinositol-4-phosphate 5-kinase activity	0.050
GO:0042264	Peptidyl-aspartic acid hydroxylation	0.035
*Positive correlation*		
GO:0006662	Glycerol ether metabolic process	0.008
GO:0042775	mitochondrial ATP synthesis coupled electron transport	0.017
GO:0001522	Pseudouridine synthesis	0.006
GO:0003879	ATP phosphoribosyltransferase activity	0.047
GO:0009982	Pseudouridine synthase activity	0.005
GO:0005388	Calcium-transporting ATPase activity	0.024
GO:0016847	1-aminocyclopropane-1-carboxylate synthase activity	0.018
GO:0042218	1-aminocyclopropane-1-carboxylate biosynthetic process	0.017
GO:0015035	Protein disulfide oxidoreductase activity	0.012
GO:0009062	Fatty acid catabolic process	0.003
GO:0015020	Glucuronosyltransferase activity	0.008
GO:0047661	Amino-acid racemase activity	0.025
GO:0009252	Peptidoglycan biosynthetic process	0.008
GO:0016303	1-phosphatidylinositol-3-kinase activity	0.037
GO:0036092	Phosphatidylinositol-3-phosphate biosynthetic process	0.050
GO:0042823	Pyridoxal phosphate biosynthetic process	0.001
GO:0019213	Deacetylase activity	0.015

Only categories that are negatively and positively correlated are shown.

## Discussion

### smRNA diversity in *Symbiodinium microadriaticum*

It is now well established that miRNAs play a central role in gene regulation in plants, animals, and yeast [60]. Only recently, a number of studies started looking into smRNA diversity in unicellular eukaryotes and discovered a rich repertoire of miRNAs, which include lineage-specific as well as previously identified miRNAs from plants or animals [61-64]. However, re-analysis of these data under a set of stringent criteria formulated by Tarver *et al*. [19] indicated firstly that among analyzed protists only brown and green algae possess miRNAs, and secondly that no miRNAs have been identified (yet) that are shared between plants, animals, and protists. Here, we studied smRNA expression in *Symbiodinium*, the photosynthetic dinoflagellate symbiont of scleractinian corals, over 9 different treatments in parallel with RNASeq. Our aim was three-fold: firstly, to characterize smRNA and mRNA diversity and expression in *Symbiodinium*, secondly to identify proteins of the RNAi machinery, and thirdly to correlate smRNA and mRNA diversity and expression. Our study represents the most comprehensive smRNA and mRNA data set for a dinoflagellate to date, and we identified a set of 21 smRNAs as well as 58,649 genes.

None of the 21 identified smRNAs were identified in previous miRNA screens of unicellular protists, and we did not identify known miRNAs from animals or plants. Note that this is despite the fact that we were assaying 9 different conditions, and accordingly, were able to query a much larger sequencing space than previous protist studies [64,65]. Furthermore, we were able to independently verify smRNAs over different experimental treatments potentially reducing the number of false positives considerably. In our set of 21 smRNAs, we identified 8 miRNAs and 13 siRNAs indicating that *Symbiodinium* not only produces miRNAs, but also siRNAs. Interestingly, within the set of smRNAs we found candidates that had only processed guide and passenger sequences, but not products originating from the hairpin loop (Additional file 1). This gives rise to the possibility of a two-step dicer process when cleaving the guide-passenger duplex from the hairpin loop, and warrants further examination.

The lengths of smRNA precursors from our set of 21 *bona fide* smRNAs were between 80–90 nt, which is between the sizes for animal (60–70 nt) and plant miRNAs (e.g. *Arabidopsis thaliana*: 59–689 nt) [66]. We note that due to constraints of the miRDeep2 core algorithm, smRNA precursors longer than 90 nt could not be identified in our approach. Furthermore, miRNA processing in animals takes place in the nucleus and cytoplasm using the endonucleases Drosha and Dicer, respectively. In plants, all miRNA processing takes place in the nucleus by Dicer [6]. We did not identify a Drosha homolog (data not shown). However, we found a homolog of HEN1 that is involved in the biogenesis of small functional RNAs, such as siRNAs and piRNAs in all metazoans [57].

Identifying non-conserved miRNAs but conserved Dicer and Argonaute proteins is in line with the hypothesis that the protein machinery to process miRNAs has a common evolutionary origin, whereas the set of generated miRNAs is lineage-specific [19]. The presence of miRNAs in single-celled dinoflagellates in itself is surprising, but functional processes that involve miRNAs in multicellular organisms (e.g. gene expression regulating metabolism, development, epigenetic inheritance) might be of significance in protists too. Interestingly, although we were focusing on the identification of miRNAs in *Symbiodinium*, 13 of the 21 smRNAs identified by miRDeep2 could be categorized as siRNAs as indicated by the perfect complementarity of the guide passenger duplex. One explanation for this is that the typical pre-miRNA hairpins were not considered initially, so that siRNAs with perfect complementary base pairing of the hairpins were identified as well.

### smRNA target genes in *Symbiodinium microadriaticum*

miRNA target identification was conducted by searching for sites that adhered to the general criteria for animal- and plant-like targets, as no functionally validated target sites of closely related species are available [15]. For mammalian miRNA targets, the rate of false positives is commonly reduced by looking for evolutionary conservation between species as well as the presence of experimentally validated target properties (e.g. an adenosine ‘anchor’ at position 1 of the miRNA-mRNA binding site) [16]. Here, we tried to increase stringency by considering target accessibilities (*ΔΔG* < −10 kcalmol^-1^) and the multiplicity of target sites, both of which have been shown to be important features beyond the seed pairing [17,67].

Mapping of our set of 21 *bona fide* smRNAs to animal- and plant-like targets identified a suite of potential genes that are under smRNA regulation, and we identified considerably more animal- than plant-like targets. Please note that whereas the criteria for animal-like target identification are somewhat relaxed (by the nature of animal-like target sites), we allowed for only 3 mismatches between miRNA-mRNA plant-like pairings after false positive estimation via randomized smRNAs. The signal-to-noise ratio of alignments with less than three mismatches was about 1:5 suggesting that the identified miRNA-mRNA pairings were highly specific. Subsequent GO analysis of predicted target genes identified a common set of processes that were enriched in animal- and plant-like target genes (i.e. protein modification and metabolism), although a larger number of significant GO terms were produced for animal-like target genes.

Following our above reasoning that different lineages possess their distinct set of miRNAs, the characteristics of corresponding mRNA target sites need to be determined experimentally for final proof. Further studies incorporating methods that crosslink Argonaute proteins together with a bound miRNA and the matching mRNA, e.g. HITS-CLIP [68], will unequivocally reveal the nature of miRNA-mRNA target binding in *Symbiodinium*. Similarly, knocking down Dicer will reveal the nature of miRNA biogenesis in *Symbiodinium*[69].

### *Symbiodinium microadriaticum* transcriptome and expression

Our transcriptome assembly produced a set of 58,649 genes, which is in the range of what has been determined previously [21]. 43.12% of all genes in the transcriptome could be annotated via BLASTX, which is also close to what has been found previously [21,28]. Interestingly, more than 90% of all genes were expressed under any condition. We found a low number of differentially expressed genes between the different treatments on average, but this finding might be limited by the low statistical power of the analysis. However, previous studies also suggest that transcriptional regulation is scarce in dinoflagellates, which would be explained by a paucity of transcription factors [21,22,29,70,71]. Further, the two most common transcription factor domains we identified in *Symbiodinium* (CCCH zinc finger and cold shock domain) may bind RNA rather than DNA [72-74]. On the other hand, the 36°C heat shock treatment produced a remarkably high number of differentially expressed genes with the majority of genes being downregulated. It remains to be determined whether exposure to this temperature involved a coordinated environmental shock response (ESR) [75], or whether we rather measured the dysregulation of gene expression and the collapse of the transcriptional machinery resulting in a subsequent downregulation of gene expression across the board.

Treatments related to similar physiologies (e.g. high temperature: 34°C vs. 36°C, ionic stress: 20 g vs. 60 g) produced overlapping sets of enriched GO terms. For instance, ionic stress-related treatments (i.e. 20 g and 60 g) produced a common set of 22 downregulated genes assorted into 15 processes, the majority of which were related to photosynthesis. Chloroplasts have been shown to be one of the most susceptible systems to salt and osmotic stresses [76], and studies in cyanobacteria showed that ionic stress in combination with light stress stimulates repair inhibition of photosystem II [77].

Among the differentially expressed genes of all treatments were genes related to photosynthesis. Accordingly, photosynthesis-related GO terms were enriched in almost all treatments. It has been shown previously that two of the core photosystem genes (*psbA* and *psaA*) are subject to transcriptional regulation under temperature stress in *Symbiodinium*[78]. Further,* psbA* and *psaA* are both encoded on so-called minicircles. Most of the genes from the chloroplast genome in dinoflagellates are not physically linked on one molecule but are located on these small plasmids [79]. Most interestingly, in our data differentially expressed genes contributing to the photosynthesis-related GO enrichment contained exclusively genes that were encoded on minicircles. Accordingly, minicircle-encoded genes might adhere to different mechanisms of transcriptional regulation than genomically-encoded loci, and this might be one of the evolutionary driving forces behind minicircles.

### Integrating smRNA and mRNA expression profiling

Our correlation analysis of smRNA and mRNA expression identified a large number of genes whose expression was highly correlated to the expression of distinct smRNAs. While we almost found an equal number of positively and negatively correlated genes, the notion that only a very small overlap of genes was correlated to the expression of more than one smRNA implies that there is some level of specificity. Additionally, the number of correlated genes for distinct smRNAs was between 66 and 598 indicating non-random smRNA target specificity, and also that ‘small effect size’ and ‘large effect size’ smRNAs might exist in *Symbiodinium*. Brennecke et al. [80] provided evidence that an average miRNA has approximately 100 target sites, and our estimates are within this order of magnitude.

Our downstream analysis of GO term enrichment for target sites revealed a noticeable overlap between enrichment of positively and negatively correlated processes providing independent support to the control of these processes through smRNAs. Within the GO category enrichment analysis a variety of processes were identified (e.g. protein modification, signaling pathways, regulation of immune responses, and chromatin silencing by small RNA) that were identified before in smRNA target screens [6,81-84]. Our data indicate that smRNAs potentially regulate a large fraction of protein-coding genes in *Symbiodinium*, and that the regulation is smRNA-specific as implied by the small overlap of correlated genes between smRNAs. Last, a multitude of processes are potentially prone to regulation by smRNAs as evidenced by the broad variety of GO terms identified, but it is interesting to note that the majority of these processes can be assorted to protein modification, immunity, signaling, DNA damage, gene expression, translation, and metabolism.

## Conclusions

In the past decade, miRNAs have been uncovered as key regulators of gene expression at the post-transcriptional level. In this study we generated and analyzed a comprehensive smRNA and mRNA expression data set over 9 experimental treatments in order to gain insights into smRNA and mRNA diversity and expression in *Symbiodinium.* The paucity of transcription factor domain-bearing proteins, and the fact that the most common domains may be RNA rather than DNA binding poses the question as to exactly how *Symbiodinium* is regulating transcription. Part of the answer to this might come from our analysis of smRNAs in *Symbiodinium*. After application of stringent criteria, we identified a set of 21 distinct and previously unidentified *bona fide* miRNAs and siRNAs alongside the corresponding core protein machinery for smRNA processing. These data together with our analyses of smRNA gene targets and smRNA-mRNA expression correlation indicate that RNAi is operational in *Symbiodinium* and potentially hundreds of genes and processes could be under smRNA control. The properties of identified smRNAs and the structure of potential mRNA target sites fall between the criteria established for animals and plants, but long siRNA precursor hairpins and the lengths of pre-miRNAs as well as the existence of highly specific miRNA plant-like target sites might argue for plant-like smRNAs in *Symbiodinium*. Our data corroborate previous analyses that RNAi core proteins are conserved and have a common evolutionary ancestor, whereas the smRNAs originating from the machinery are lineage-specific. Overall, the emerging picture is that dinoflagellates are not only distinct in terms of genome size, content, and transcriptional regulation, but also rival the complexity of multicellular eukaryotes as evidenced by the presence of a rich set of smRNAs and the corresponding protein machinery. Importantly, the functional significance of RNA-dependent control of organismal processes in single-celled eukaryotes, and their degree of evolutionary conservation, have yet to be determined and await further study.

## Methods

### Culture and experimental treatments

*Symbiodinium microadriaticum* (clade A1, strain CCMP2467, strain synonym 370, National Center for Marine Algae and Microbiota), originally isolated from its *Stylophora pistillata* host at Aqaba, Jordan, was cultured at 23°C in f/2 medium [85] on a 12 h/12 h day/night regime (daytime: 6 am to 6 pm; night-time: 6 pm to 6 am, light intensity 80 μmolm^-2^ s^-1^). The salt content in the medium was set to 40 g/l, matching the average salinity characteristic of the Red Sea. The source culture was checked for bacterial and protist contamination before small volumes were subjected to growth and subsequent application of experimental treatments. We omitted the use of antibiotics in order to exclude any potential contribution of antibiotic treatment to the expression of smRNAs and mRNAs in cultures. Exponentially growing cells were harvested at noon, at the middle of the cultures’ daytime phase to represent a smRNA/mRNA reference (labeled noon: 12 h/12 h day/night). As we were interested in investigating the diversity and dynamics of expressed smRNAs and mRNAs in *S. microadriaticum*, we subjected cultures to 8 additional treatments. Briefly, we subjected cultures to cold shock (labeled 4°C: 4°C for 4 hours), cold stress (labeled 16°C: 16°C for 4 hours), heat stress (labeled 34°C: 34°C for 12 hours), heat shock (labeled 36°C: 36°C for 4 hours), hyposalinity (labeled 20 g: 20 g/L NaCl salt content for 4 hours), hypersalinity (labeled 60 g: 60 g/L NaCl salt content for 4 hours), dark stress (labeled DS: 18 hour dark period), and dark cycle (labeled DC: 12 h/12 h day/night cycle, sampled at midnight). In all cases, separate exponentially growing *S. microadriaticum* cultures were subjected to the treatment conditions and harvested at the end of experimental treatment before they reached 5x10^6^ cells/ml in order to avoid stationary phases that yield lower RNA quality.

### RNA isolation and sequencing

For total RNA isolation, 50 ml of cells were pelleted by spinning cultures at 3,000 × g for 5 minutes and subsequent washing with RO water. Pellets were snap frozen in liquid nitrogen and cells were ground with approximately 300 – 500 mg 0.1 mm silica beads (Biospec, Bartlesville, OK) under liquid nitrogen to break cell walls and membranes. Small RNA and total RNA fractions were selectively extracted from the same pellet using the mirVana miRNA Isolation Kit (Ambion, Austin, TX) according to manufacturer’s instructions. All RNA isolations were quality-checked using Bioanalyzer (Agilent, Santa Clara, CA) and NanoDrop (ThermoScientific, Wilmington, DE) prior to library creation and sequencing by the KAUST Bioscience Core lab. For mRNA sequencing, 2 × 100 bp paired-end reads for Illumina sequencing were generated from oligo(dT) selected total RNA using the Illumina TruSeq RNA Sample Prep Kit (Illumina, San Diego, CA) according to manufacturer’s instructions. Sequence libraries for small RNAs were created with the Illumina TruSeq Small RNA Sample Prep Kit (Illumina, San Diego, CA) according to manufacturer’s instructions. mRNA sequencing libraries for the different conditions were multiplexed in equal quantities and ran on three lanes on the Illumina HiSeq 2000 platform producing a total of 302 million paired-end reads. Small RNA libraries were sequenced on 4 lanes on an Illumina Genome Analyzer IIx (GA2x) and produced a total of 137 million small RNA reads ≤ 32nt. All small RNA and RNASeq data are available in the NCBI Gene Expression Omnibus database under accessions GSE47373 and GSE47372. The transcriptome assembly is available in the NCBI Transcriptome Shotgun Assembly Sequence Database under accession GAKY00000000.

### Data processing and identification of smRNAs

From the raw FASTQ reads, low quality 3′ ends were trimmed to produce reads with 3′ ends having a Phred score of > 20, while the average Phred score of the entire read was > 20 as well. Further, the overall quality of each read was assessed by the probability of incorrect base calls under implication of the read length. The Illumina 5′ and 3′ sequencing adapters were trimmed with Cutadapt v1.0 [86] and the small RNA libraries were further filtered to a minimum length of 18 nt. In order to remove sequences matching known rRNA, tRNA, and mRNA sequences, reads were assembled into short contigs with Velvet [87]. Assembled contigs that matched known non-coding RNAs (rRNAs, tRNAs, snoRNAs) in the NCBI nt database or contigs matching assembled transcript sequences of *S. microadriaticum* were removed from further analyses.

*Symbiodinium* miRNAs were identified with miRDeep2 [42,88]. Briefly, pre-miRNAs were predicted by miRDeep2 using a draft genome assembly of *Symbiodinium microadriaticum* and subsequently verified by assessing position and frequency of small RNA reads that match to predicted guide, loop, and passenger sequences. This procedure was conducted independently for each of the 9 treatments. We applied a conservative approach for *de novo* miRNA annotation: only miRNAs predicted with a signal-to-noise ratio of 10:1 by miRDeep2 were further examined. For verification of candidates, we followed the criteria for miRNA identification conserved among plant and bilaterian miRNAs [19,89]. Briefly, a miRNA had to fulfill the following criteria to be considered in the final dataset: (1) A distinct 5′ terminus of the mapped miRNA (guide strand) and miRNA* (passenger strand), (2) the presence of a 2 nucleotide 3′ overhang of the miRNA-miRNA* duplex, and (3) a pre-miRNA fold-back structure that had a minimum fold energy (MFE) < −25 kcal mol^-1^[90]. We considered small RNAs *bona fide* miRNAs if they were predicted in a minimum of 7 conditions and if the respective miRNA* sequence was found in at least 2 conditions.

### smRNA target gene prediction

The search for potential smRNA target genes followed criteria known from animal and plant model organisms [6,15,91]. Since miRNA target sites in animals are characterized by short complementary regions in the 5′ region of a miRNA to the UTR of a gene [15], we were looking for reverse complementary 6mer matches (so-called seeds) of the miRNAs 5′ nucleotides 2–7 to the 3′ UTRs of 12,858 mRNAs with bowtie [92] (no mismatches allowed). UTRs of mRNAs were predicted by MAKER [93] based on transcriptomic and genomic data. The seed matches were further classified by the additional complementary pairing around the seed to the 3 canonical target sites: 7mer- m8 (seed match + complementary match at position 8), 7mer-1A (seed match + adenine at position 1), and 8mer (seed match + adenine at position 1 and complementary match at position 8) according to Bartel *et al.*[15]. Plant-like miRNA silencing is highlighted by a near perfect match between the entire length of the miRNA to the CDS or UTR of the corresponding mRNA [15]. Accordingly, target prediction was performed by reverse complement alignment of the miRNA to the CDS of 51,917 genes (predicted from transcriptomic and genomic data, in the following referred to as the genomic gene set) as well as to the 3′ UTRs of 12,858 of these mRNAs. To estimate number of false positives in plant-like targets, the script ‘random_dna_strings.pl’ (http://tata-box-blog.blogspot.com/2011/06/perl-script-to-generate-n-random-dna.html) was used to generate 1,000 sets of random miRNA sequences with the same overall base composition as the native small RNAs. These were subsequently aligned in the same way as described above. Alignments were then ranked by the number of mismatches and compared to the mismatch counts of the miRNA target alignments.

Further assessment of miRNA targets was based on target site accessibility of the mRNA secondary structure with PITA [67]. The accessibility for the miRNA target site (*ΔΔG*) was calculated as the difference between the energy required to open the target mRNA secondary structure (*ΔG*_open_), including 70 nt upstream and 70 nt downstream of the target site as well as the energy gained by the miRNA binding (*ΔG*_Duplex_) [67]. Only miRNA targets with a *ΔΔG* of < −10 kcalmol^-1^ were retained. Given that 3′ UTRs can contain multiple target site copies for a single miRNA, the target accessibility of the entire UTR for a given miRNA was calculated according to the formula $\Delta \Delta G_{\mathrm{Score}}=1n\left(\sum {}_{1}^{n}e^{-\Delta \Delta G_{n}}\right)$[67].

Respective animal- and plant-like target sets were analyzed for GO category enrichment using the Adrian Alexa’s weighted scoring algorithm implemented in GOEAST [50] employing a *P* value cutoff of 0.05. The resulting *P* values were not corrected for multiple testing since the Alexa algorithm performs non-independent tests, i.e. the *P* value computed for a given GO term is conditional on neighboring terms. Therefore the multiple testing theory does not apply and the *P* values provided are considered adjusted [94].

### smRNA expression

Small RNA read counts were calculated with the quantifier.pl script of the miRDeep2 package [42] to calculate expression. Briefly, read counts of all identified smRNAs as well as small RNAs that featured 1 additional nucleotide at the 5′ terminus and/or up to 3 additional nucleotides at the 3′ terminus were taken into account and summed up. The smRNA libraries were scaled by the geometric mean normalization method implemented in DESeq 1.12.0 [95] and tested for differential expression via pairwise comparison of experimental treatments (i.e. 4°C, 16°C, 34°C, 36°C, 20 g, 60 g, DC, DS) to the selected reference condition noon with an FDR of 0.1.

### Data processing of mRNA libraries, transcriptome assembly, and expression

Raw paired-end reads from RNAseq data were trimmed using TrimBWAstyle.pl (http://wiki.bioinformatics.ucdavis.edu/index.php/TrimBWAstyle.pl) to remove low quality (Phred ≤ 4) trailing nucleotides from reads. Using k-mer counts from the software Jellyfish [96], reads were corrected with a conservative cutoff of 1.5. This correction process resulted in the trimming or removal of reads rather than error correction *per se*. The reads were subsequently error corrected using Quake [97] in order to remove very low abundant k-mers and reduce the memory footprint of later assembly steps. Jellyfish [96] was further used to record quality weighted counts of all 19mers in the data set. Subsequently, Oases [98] was used to assemble read pairs into a set of putative transcripts and corresponding loci. The assembly was carried out with the recommended protocol described in Schulz et al. [98]. The average insert size of paired-end reads was inferred by Velvet [87]. This procedure generated an assembly with 87,010 genes (or loci) and 250,046 putative transcripts.

Transcript counts were derived using bowtie2 [99]. Briefly, reads from each treatment were mapped against the assembled transcriptome using the options ‘-a -t --no-unal --rdg 6,5 --rfg 6,5 --score-min L,-.6,-.4 --no-discordant --no-mixed -p 7 --phred64 –fr’ to report all alignments. The output was analyzed with the eXpress software [100] to obtain effective read counts and FPKM (Fragments Per Kilobase of transcript per Million mapped reads) values. Effective read counts for genes were obtained by adding counts of all transcripts of this gene. Expression estimates were only retained for genes with transcripts of at least 250 bp as we found an overrepresentation of read counts for short transcripts. This yielded expression estimates for 58,649 genes. Significantly differentially expressed genes were determined by pairwise comparisons of the selected noon reference to the eight additional treatments with the DESeq software using a FDR < 0.1 [95]. Due to the lack of replicated treatments, expression dispersion was calculated across conditions. The underlying assumption is that most genes behave the same within replicates as across conditions (in line with the assumption of comparatively few differentially expressed genes). This procedure commonly yields dispersion estimates that are higher than with replicated treatments, resulting in a more conservative estimate of differential expression. Scaled smRNA and mRNA expression estimates from DESeq were correlated along all 9 treatments with Spearman’s rank correlation coefficient via the cor.test() function in the R statistical package [101]. Only smRNA-mRNA pairs with a correlation coefficient *Rho* > 0.8 or < −0.8 were retained and analyzed for enrichment of GO categories via GOEAST [50] using a *P* value cutoff of 0.05.

### Transcriptome annotation and Identification of RNAi core proteins

We annotated the assembled genes by selecting the longest transcript of each gene. The resulting sequence set was consecutively searched against SwissProt, TrEMBL, and NCBI nr using BLASTX [102] and an e-value cutoff of 1e^-5^. If a transcript produced a hit against SwissProt, this was used for annotation. If not, the best hit against TrEMBL was used. In absence of such a hit the best match to NCBI nr was used for annotation. Gene Ontology categories were assigned via the BLASTX hit to SwissProt or TrEMBL databases and subsequent mapping to the UniProt-GOA project [103].

To identify genes with transcription factor (TF) domains we assembled a list of 195 TF DNA binding domains represented by Hidden Markov Model (HMM) profiles in the Pfam database [104]. The HMMER3 program HMMScan [105] was used to search six-frame translations of all transcripts against these domains with an e-value cutoff of 1e^-5^. Genes with one or more transcripts that had a hit to one or more TF domains were counted as TF genes.

Sequences from the two core RNAi protein families (i.e. Argonaute and Dicer) and from the small RNA 2′-O-methyltransferase HEN1 were retrieved from five model organisms (*H. sapiens, D. melanogaster, C. elegans, S. pombe*, and *A. thaliana*) from UniProtKB [51] and clustered into groups with 90% sequence identities in order to generate consensus sequences of the three protein families. These consensus sequences were searched against the translated set of genomic genes from *Symbiodinium microadriaticum* (n = 51,917) with BLASTP. BLAST hits with e-values < 1e^-10^ were then queried for domains that are required for the catalytic function of the protein using InterProScan [106-108]. The crucial domains were: a pair of RNase III domains, and a PAZ domain for Dicer homologs; PAZ and Piwi domains for Argonaute proteins; and a methyltransferase domain for the small RNA 2′-O-methyltransferase HEN1. Using Clustal Omega [109], homologs were then aligned against all known RNAi proteins from the five model organisms on a per-protein basis. Resulting alignments aided the identification of conserved residues in the protein domains associated with RNAi activity. Jalview [110] was used to visualize the alignments.

## Competing interests

The authors declare no conflict of interests.

## Authors’ contributions

CRV, GM, and MA designed and conceived the experiments. MA, YJL, and AC generated data. SB, TB, YJL, and CRV analyzed data. CRV and SB wrote the manuscript. All authors have read and approved the final manuscript.

## Authors’ information

SB is currently completing his Master thesis at the King Abdullah University of Science and Technology (KAUST) in the lab of CRV. SB’s interest lies in the contribution of smRNAs to the regulation of symbiotic relationships. CRV is currently an Assistant Professor in the Red Sea Research Center at KAUST. CRV’s main research direction is ecogenomics with a focus on marine ecosystems. His group is applying high throughput molecular tools to the elucidation of the genes in ecology and the ecology in genes with an emphasis on the understanding and evolution of interspecies relationships.

## Supplementary Material

Additional file 1: miRDeep2 output of the 21 identified smRNAs. Click here for file

Additional file 2 Table detailing the annotated animal-like targets assorted by target seed properties (n = 1,720 in 3'UTRs of 12,858 mRNAs). Click here for file

Additional file 3 Table detailing the annotated plant-like targets assorted by seed matching to CDS or UTR (n = 19 in 51,917 genes from the genomic gene set). Click here for file

Additional file 4 **Annotation of genes from the *Symbiodinium microadriaticum* transcriptome assembly.** Click here for file

Additional file 5 FPKM expression estimates for 58,649 genes assorted by experimental treatment. Click here for file

Additional file 6 Details the differentially expressed genes (DEGs) assorted by fold change and experimental treatment (n = 3,285 DEGs in 58,649 genes, DESeq, FDR < 0.1). Click here for file

Additional file 7 **Table detailing enriched GO terms within the differentially expressed genes assorted by experimental treatment (Fisher's Exact test, Alexa algorithm, *P *< 0.05).** Click here for file

Additional file 8 **Alignment of functional domains of the *Symbiodinium microadriaticum *homolog of the endoribonuclease Dicer with homologs from model organisms (*S. pombe, A. thaliana*, *C. elegans*, *D. melanogaster*, *H. sapiens*).** Key functional residues are depicted with red asterisks. Click here for file

Additional file 9 **Alignment of functional domains of *Symbiodinium microadriaticum *homologs of the Argonaute effector protein with homologs from model organisms (*S. pombe, A. thaliana***, ***C. elegans*, *D. melanogaster*, *H. sapiens*).** Key functional residues are depicted with red asterisks. Click here for file

Additional file 10 **Alignment of functional domains of the *Symbiodinium microadriaticum *homolog of the small RNA 2'-O-methyltransferase (HEN1) with homologs from model organisms (*S. pombe, A. thaliana*, *C. elegans***, ***D. melanogaster*, *H. sapiens*).** Key functional residues are depicted with red asterisks. Click here for file

Additional file 11 Table detailing enrichment of GO terms of negatively and positively correlated smRNA-mRNA expression pairs. Click here for file
