# Supplementary material for: Integrating microRNA and mRNA expression profiling in Symbiodinium microadriaticum, a dinoflagellate symbiont of reef-building corals
Source: BMC Genomics. 2013 Oct 12;14:704. doi: 10.1186/1471-2164-14-704 (PMC3853145; doi:10.1186/1471-2164-14-704)
Supplement: Additional file 1: — miRDeep2 output of the 21 identified smRNAs. [file 1471-2164-14-704-S1.pdf]

Provisional ID	:	3826332_77065_2337952_3782493+,...,3787871+_24618
Score total	:	7503.4
Score for star read(s)	:	3.9
Score for read counts	:	7495.6
Score for mfe	:	2.9
Score for randfold	:	1.6
Score for cons. seed	:	-0.6
Total read count	:	14714
Mature read count	:	14248
Loop read count	:	26
Star read count	:	440

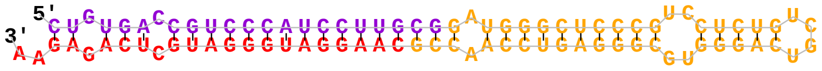

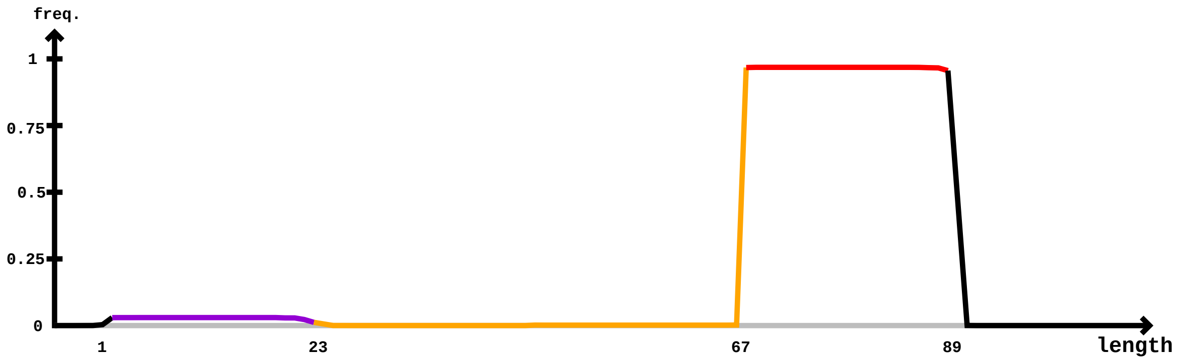

Star **Mature**

```
5'- uuuucugugaccgucccauccuugcggaugggcucccguccucugucgucagggugcgggaguccaaccgcaaggaugggaugcucagagaaaagcugaggaggcccaagga   -3'    obs
    uuuucugugaccgucccauccuugcggaugggcucccguccucugucgucagggugcgggaguccaaccgcaaggaugggaugcucagagaaaagcugaggaggcccaagga          exp
    ((((((.(((.((((((((((((((((.((((((((((..(((((....)))))..)))))))))).)))))))))))))))).))).)))))).((..((....))..)).   reads     mm      sample
    .uGucugugaccgucccauccuugc.......................................................................................   1         1       smb
    ..uucugugaccgucccauccuugU.......................................................................................   2         1       smb
    ...ucugugaccgucccauccu..........................................................................................   3         0       smb
    ...ucugugaccgucccauccuug........................................................................................   5         0       smb
    ...ucugugaccgucccauccuugc.......................................................................................   40        0       smb
    ...ucugugaccgucccauccuugU.......................................................................................   1         1       smb
    ....cugugaccgucccauccu..........................................................................................   15        0       smb
    ....cugugaccgucccauccuu.........................................................................................   2         0       smb
    ....cugugaccgucccauccuug........................................................................................   80        0       smb
    ....cugugacUgucccauccuug........................................................................................   1         1       smb
    ....cugugaccgucccauccuuU........................................................................................   1         1       smb
    ....cugugaccAucccauccuugc.......................................................................................   1         1       smb
    ....cugugaccgucccauccuugc.......................................................................................   90        0       smb
    ....cugugaccgucccauccuugA.......................................................................................   1         1       smb
    ....cugugaccgucccauAcuugc.......................................................................................   1         1       smb
    ....cugugaccgucccauccuugU.......................................................................................   21        1       smb
    ....cugugaccgucccauccuuAcg......................................................................................   1         1       smb
    ....cugugaccgucccauccuugcU......................................................................................   2         1       smb
    ....cugugaccgucccauccuugcg......................................................................................   170       0       smb
    .....ugugaccgucccauccuug........................................................................................   1         0       smb
    .....ugugaccgucccauccuugcg......................................................................................   1         0       smb
    .......ugaccgucccauccuugcggau...................................................................................   1         0       smb
    .........................ggaugggcucccguccucugucgu...............................................................   2         0       smb
    ................................................ucagggugcgggaguccaacc...........................................   2         0       smb
    ................................................ucagggugcgggaguccaaccU..........................................   1         1       smb
    ................................................ucagggugcgggaguccaaccg..........................................   18        0       smb
    ................................................ucagggugcgggaguccaaccgU.........................................   3         1       smb
    .....................................................................gcaaggaugggaugcucagaga.....................   1         0       smb
    .....................................................................Ccaaggaugggaugcucagaga.....................   1         1       smb
    .....................................................................Ccaaggaugggaugcucagagaa....................   4         1       smb
    ......................................................................caaggaugggaugcucag........................   2         0       smb
    ......................................................................caaggaugggaugcucagU.......................   2         1       smb
    ......................................................................caaggaugggaugcucaga.......................   15        0       smb
```

```
                Star                                                 Mature
uuuucugugaccgucccauccuugcggaugggcucccguccucugucgucagggugcgggaguccaaccgcaaggaugggaugcucagagaaaagcugaggaggcccaagga
......................................................................caaggaugggaugcucagaA......................    2       1       smb
......................................................................caaggaugggaugcucagaU......................    4       1       smb
......................................................................caaggaugggaugcucagag......................    5       0       smb
......................................................................caaggaugggaugcucagaAa.....................    1       1       smb
......................................................................caaggaugggaugcucagaga.....................    142     0       smb
......................................................................caaggaugggaugcucagagU.....................    5       1       smb
......................................................................caaggaugggaugcucagagaG....................    5       1       smb
......................................................................cGaggaugggaugcucagagaa....................    2       1       smb
......................................................................caaggaugggaugcuUagagaa....................    2       1       smb
......................................................................caaggaugggaugUucagagaa....................    3       1       smb
......................................................................caaggaugggaGgcucagagaa....................    3       1       smb
......................................................................caaggauAggaugcucagagaa....................    1       1       smb
......................................................................cCaggaugggaugcucagagaa....................    2       1       smb
......................................................................caaggaugAgaugcucagagaa....................    1       1       smb
......................................................................caaggaugggaugcucGgagaa....................    4       1       smb
......................................................................caaggaugggaugcucagaCaa....................    2       1       smb
......................................................................caaggaugggUugcucagagaa....................    7       1       smb
......................................................................caaggauggCaugcucagagaa....................    2       1       smb
......................................................................caaggaugggauAcucagagaa....................    3       1       smb
......................................................................caaggUugggaugcucagagaa....................    1       1       smb
......................................................................caaggaugggaugcCcagagaa....................    4       1       smb
......................................................................caaCgaugggaugcucagagaa....................    1       1       smb
......................................................................caaggaugggaugcucagCgaa....................    1       1       smb
......................................................................caaggaugggaugcucagagaU....................    37      1       smb
......................................................................caaggaugggauUcucagagaa....................    1       1       smb
......................................................................cUaggaugggaugcucagagaa....................    1       1       smb
......................................................................caaggaugggaCgcucagagaa....................    3       1       smb
......................................................................caaggaugggaugcuGagagaa....................    1       1       smb
......................................................................caaggaugUgaugcucagagaa....................    2       1       smb
......................................................................caaggaCgggaugcucagagaa....................    2       1       smb
......................................................................caaggaugggaugcucagagaa....................    13138   0       smb
......................................................................caGggaugggaugcucagagaa....................    2       1       smb
......................................................................caaggaugggaugcucaAagaa....................    2       1       smb
......................................................................caaggaugggaugcAcagagaa....................    2       1       smb
......................................................................caaggaugggGugcucagagaa....................    2       1       smb
......................................................................caaAgaugggaugcucagagaa....................    1       1       smb
......................................................................caaggauggAaugcucagagaa....................    1       1       smb
......................................................................caaggaugggaugcucagagGa....................    1       1       smb
......................................................................caaggaAgggaugcucagagaa....................    1       1       smb
......................................................................caaggGugggaugcucagagaa....................    1       1       smb
......................................................................caaggauUggaugcucagagaa....................    6       1       smb
......................................................................Uaaggaugggaugcucagagaa....................    3       1       smb
......................................................................caaggaugggaAgcucagagaa....................    3       1       smb
......................................................................caaUgaugggaugcucagagaa....................    3       1       smb
......................................................................caaggaugCgaugcucagagaa....................    1       1       smb
......................................................................caaggaugggaugcucagGgaa....................    5       1       smb
......................................................................caagAaugggaugcucagagaa....................    2       1       smb
......................................................................caagUaugggaugcucagagaa....................    1       1       smb
......................................................................caagCaugggaugcucagagaa....................    1       1       smb
......................................................................caaggauggUaugcucagagaa....................    3       1       smb
......................................................................Gaaggaugggaugcucagagaa....................    1       1       smb
......................................................................caaggaugggaugcucagagaaC...................    2       1       smb
......................................................................caaggaugggaugcucagagaaU...................    770     1       smb
......................................................................caaggaugggaugcucagagaaa...................    3       0       smb
......................................................................caaggaugggaugcucagagaaUa..................    2       1       smb
......................................................................caaggaugggaugcucagagaaaG..................    1       1       smb
......................................................................caaggaugggaugcucagagaaaU..................    1       1       smb
.......................................................................aaggaugggaugcucagagaa....................    12      0       smb
.......................................................................aaggaugggaugcucagagaaU...................    2       1       smb
```

Provisional ID	:	3811157_23229_411523_11197_669303_283453_5183_40295578_11112323_10040
Score total	:	2080.1
Score for star read(s)	:	3.9
Score for read counts	:	2074.1
Score for mfe	:	2.7
Score for randfold	:	0
Score for cons. seed	:	-0.6
Total read count	:	4080
Mature read count	:	3602
Loop read count	:	38
Star read count	:	440

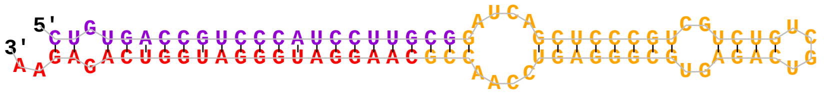

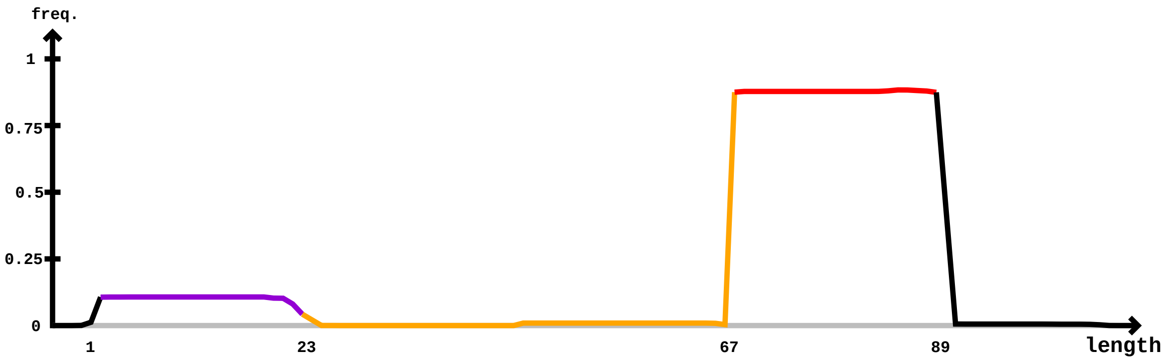

Star Mature

```
5'- auucugugaccgucccauccuugcggaucagcucccgucgucugucgucagagugcgggaguccaaccgcaaggaugggauggucagagaaaagcugaggcccaaggagga  -3'   obs
    auucugugaccgucccauccuugcggaucagcucccgucgucugucgucagagugcgggaguccaaccgcaaggaugggauggucagagaaaagcugaggcccaaggagga         exp
    .((((.((((((((((((((((((((....((((((((..((((....))))..))))))))....))))))))))))))))))))).)))).((....)).........    reads    mm    sample
    .uucugugaccgucccauccuugU.......................................................................................    2        1     smb
    ..ucugugaccgucccauccu..........................................................................................    3        0     smb
    ..ucugugaccgucccauccuug........................................................................................    5        0     smb
    ..ucugugaccgucccauccuugU.......................................................................................    1        1     smb
    ..ucugugaccgucccauccuugc.......................................................................................    40       0     smb
    ...cugugaccgucccauccu..........................................................................................    15       0     smb
    ...cugugaccgucccauccuu.........................................................................................    2        0     smb
    ...cugugacUgucccauccuug........................................................................................    1        1     smb
    ...cugugaccgucccauccuug........................................................................................    80       0     smb
    ...cugugaccgucccauccuuU........................................................................................    1        1     smb
    ...cugugaccgucccauAcuugc.......................................................................................    1        1     smb
    ...cugugaccgucccauccuugA.......................................................................................    1        1     smb
    ...cugugaccAucccauccuugc.......................................................................................    1        1     smb
    ...cugugaccgucccauccuugc.......................................................................................    90       0     smb
    ...cugugaccgucccauccuugU.......................................................................................    21       1     smb
    ...cugugaccgucccauccuugcU......................................................................................    2        1     smb
    ...cugugaccgucccauccuugcg......................................................................................    170      0     smb
    ...cugugaccgucccauccuuAcg......................................................................................    1        1     smb
    ....ugugaccgucccauccuug........................................................................................    1        0     smb
    ....ugugaccgucccauccuugcg......................................................................................    1        0     smb
    ......ugaccgucccauccuugcggau...................................................................................    1        0     smb
    ...............................................ucagagugcgggaguccaac............................................    3        0     smb
    ...............................................ucagagugcgggaguccaacc...........................................    21       0     smb
    ...............................................ucagagugcgggaguccaaccg..........................................    10       0     smb
    ...............................................ucagagugcgggaguccaaccU..........................................    4        1     smb
    ....................................................................Ccaaggaugggauggucagagaa....................    1        1     smb
    .....................................................................caaggaugggauggucag........................    1        0     smb
    .....................................................................caaggaugggauggucagU.......................    1        1     smb
    .....................................................................caaggaugggauggucaga.......................    6        0     smb
    .....................................................................caaggaugggauggucagaU......................    8        1     smb
    .....................................................................caaggaugggauggucagaga.....................    20       0     smb
    .....................................................................caaggUugggauggucagagaa....................    1        1     smb
    .....................................................................caaggauggUauggucagagaa....................    1        1     smb
```

```
    Star                                                              Mature
auucugugaccgucccauccuugcggaucagcucccgucgucugucgucagagugcgggaguccaaccgcaaggaugggauggucagagaaaagcugaggcccaaggagga
.....................................................................caaggaugggauUgucagagaa....................   1      1   smb
.....................................................................caaggauAggauggucagagaa....................   3      1   smb
.....................................................................caaAgaugggauggucagagaa....................   1      1   smb
.....................................................................caaggaugggauggucagagaG....................   2      1   smb
.....................................................................caaggaugggauggucagagaa....................   3285   0   smb
.....................................................................caaggaugggaGggucagagaa....................   1      1   smb
.....................................................................caaggaugggauggucagagaU....................   20     1   smb
.....................................................................cUaggaugggauggucagagaa....................   1      1   smb
.....................................................................caaggaugggauggAcagagaa....................   1      1   smb
.....................................................................caaggaugggauggucagaCaa....................   1      1   smb
.....................................................................caaggaugggauggucUgagaa....................   3      1   smb
.....................................................................caaggaugggaugUucagagaa....................   3      1   smb
.....................................................................caaggaugggauggucGgagaa....................   1      1   smb
.....................................................................caaggaugggauggucagaAaa....................   1      1   smb
.....................................................................caaggauUggauggucagagaa....................   1      1   smb
.....................................................................caaggaugCgauggucagagaa....................   1      1   smb
.....................................................................caaggaugAgauggucagagaa....................   1      1   smb
.....................................................................caaCgaugggauggucagagaa....................   1      1   smb
.....................................................................caaggaugggauggucagagaaU...................   222    1   smb
.....................................................................caaggaugggauggucagagaaC...................   1      1   smb
.....................................................................caaggaugggauggucagagaaUa..................   1      1   smb
......................................................................aaggaugAgauggucaga.......................   1      1   smb
......................................................................aaggaugggauggucagagaa....................   10     0   smb
....................................................................................cagagaaaagUugaggcc.........   1      1   smb
.....................................................................................agagaaaagcugaggccU........   1      1   smb
.....................................................................................agagaaaagcugaggcccaag.....   1      0   smb
.....................................................................................agagaaaagcugaggcccaagg....   6      0   smb
......................................................................................gagaaaagcugaggcccaaU.....   1      1   smb
......................................................................................gagaaaagcugaggcccaagg....   1      0   smb
......................................................................................gagaaaagcugaggcccaagga...   11     0   smb
......................................................................................gagaaaagcugaggcccaaggaU..   1      1   smb
```

Provisional ID	:	3827716_145772_4486416_3800162+,...,3747241-_26005
Score total	:	408.5
Score for star read(s)	:	3.9
Score for read counts	:	400.3
Score for mfe	:	3.2
Score for randfold	:	1.6
Score for cons. seed	:	-0.6
Total read count	:	797
Mature read count	:	495
Loop read count	:	138
Star read count	:	164

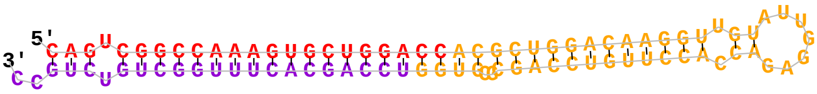

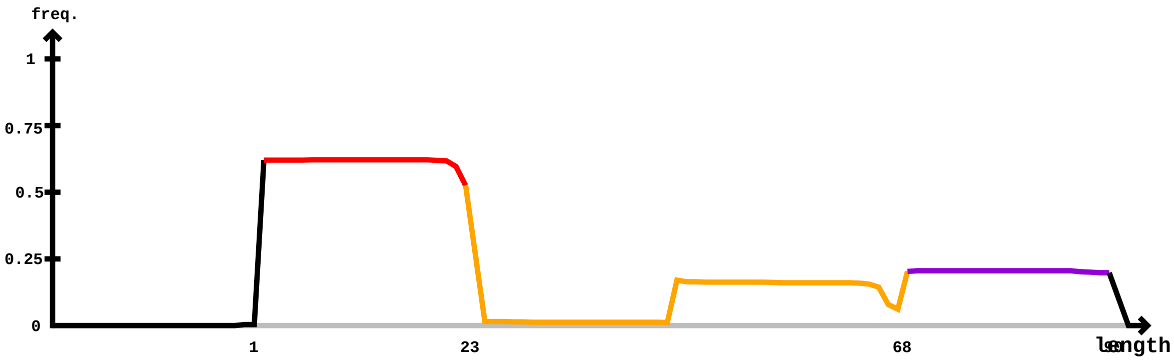


```
                     Mature                                                          Star
5'- gcccuugagagggggugugg cagucggccaaagugcuggacc acgcuggacaagguuguauuggagaccaccuuguccagcgcgugg uccagcacuuuggcugucugcc acu -3'   obs
gcccuugagagggggugugg cagucggccaaagugcuggacc acgcuggacaagguuguauuggagaccaccuuguccagcgcgugg uccagcacuuuggcugucugcc acu        exp
```

```
5'- gcccuugagagggggugugguagcagucggccaaagugcuggaccacgcuggacaagguuguauuggagaccaccuuguccagcgcgugguccagcacuuuggcugucugccacu -3'
```

```
5'- gcccuugagagggggugugg cagucggccaaagugcuggacc acgcuggacaagguuguauuggagaccaccuuguccagcgcgugg uccagcacuuuggcugucugcc acu -3'
```

```
5'- gcccuugagagggggugugg[cagucggccaaagugcuggacc][acgcuggacaagguuguauuggagaccaccuuguccagcgcgugg][uccagcacuuuggcugucugcc]acu -3'
```

```
                                    Mature                                                      Star
5'- gcccuugagagggggugugg cagucggccaaagugcuggacc acgcuggacaagguuguauuggagaccaccuuguccagcgcgugg uccagcacuuuggcugucugcc acu  -3'  obs
```

```
5'- gcccuugagagggggugugg cagucggccaaagugcuggacc acgcuggacaagguuguauuggagaccaccuuguccagcgcgugg uccagcacuuuggcugucugcc acu -3'
```

```
                    Mature                                                           Star
gcccuugagaggggguguggcagucggccaaagugcuggaccacgcuggacaaagguuguauuggagaccaccuuguccagcgcgugguccagcacuuuggcugucugccacu
................................................................gagaccaccuuguccagcgcgugg.........................   7     0     smb
................................................................gagaccaccuuguccagcgcgugU.........................   24    1     smb
........................................................................................uccagcacuuuggcuguc.......   3     0     smb
........................................................................................uccagcacuuuggcugucu......   1     0     smb
........................................................................................uccagcacuuuggcugucug.....   2     0     smb
........................................................................................uccagcCcuuuggcugucugcc...   1     1     smb
........................................................................................uccagcacuuuggcugucugcc...   122   0     smb
........................................................................................uccagcacuuuggcugucugccU..   33    1     smb
.........................................................................................ccagcacuuuggcugucugcc...   1     0     smb
.........................................................................................ccagcacuuuggcugucugccU..   1     1     smb
```

Provisional ID	:	3825705_110890_3256016_3775388+,...,3760585+_23769
Score total	:	1035.6
Score for star read(s)	:	3.9
Score for read counts	:	1028.4
Score for mfe	:	2.2
Score for randfold	:	1.6
Score for cons. seed	:	-0.6
Total read count	:	2029
Mature read count	:	1115
Loop read count	:	204
Star read count	:	710

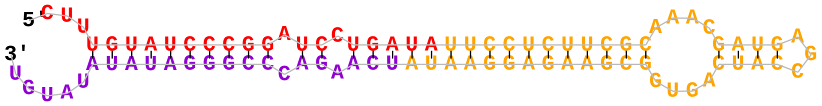

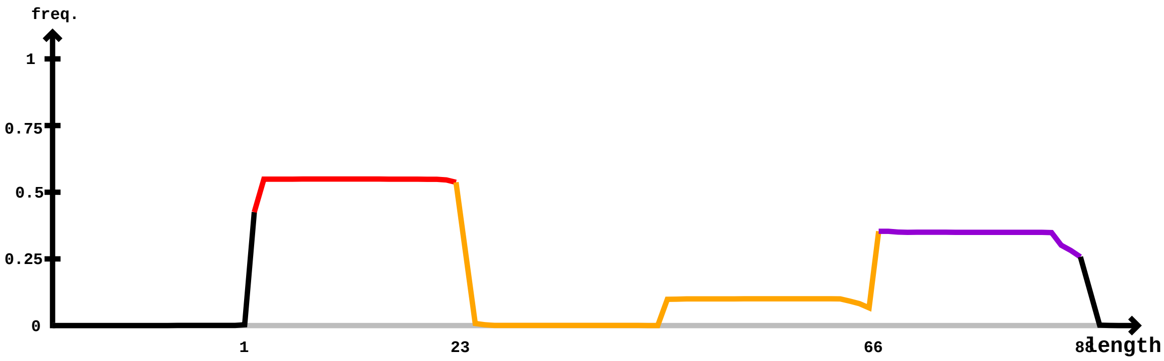

Mature Star

```
5'- cuggucaucucugcauaaccuuuguaucccggauccugauauuccucuucgcaaacgaugagccaucaguggcgaagaggaauaucaagacccgggauauauauguuauuc  -3'  obs
    cuggucaucucugcauaaccuuuguaucccggauccugauauuccucuucgcaaacgaugagccaucaguggcgaagaggaauaucaagacccgggauauauauguuauuc       exp
    ..............(((((...((((((((((.((.((((((((((((((((....((((...))))....)))))))))))))))).)).))))))))))...)))))..   reads  mm  sample
    ...........ugcauaaccuuuguaucccgga..............................................................................   1      0   smb
    ..................ccuuuguaucccggauccugau.......................................................................   5      0   smb
    ...................cuuuguaucccggauccu..........................................................................   1      0   smb
    ...................cuuuguaucccggauccuga........................................................................   5      0   smb
    ...................cuuuguaucccggauccugau.......................................................................   13     0   smb
    ...................cuuuguaucccggauccugaua......................................................................   736    0   smb
    ...................cuuuguaucccggauccugauU......................................................................   1      1   smb
    ...................cuuuCuaucccggauccugaua......................................................................   1      1   smb
    ...................cuuuguaucccggauccCgaua......................................................................   1      1   smb
    ...................cuuuguaucccggauccugUua......................................................................   1      1   smb
    ...................cuuuguaucccggauUcugaua......................................................................   1      1   smb
    ...................cCuuguaucccggauccugaua......................................................................   1      1   smb
    ...................cuuuguaucccggauccugauUu.....................................................................   1      1   smb
    ...................cuuuguauccAggauccugauau.....................................................................   1      1   smb
    ...................cuuuguaucccggauccugauau.....................................................................   77     0   smb
    ...................cuuuguaucccggauccugauaA.....................................................................   5      1   smb
    ...................cuuuguaucccggauccugauUuu....................................................................   1      1   smb
    ...................cuuuguaucccggauccugauaAu....................................................................   1      1   smb
    ...................cuuuguaucccggauccugauauA....................................................................   1      1   smb
    ...................cuuuguaucccggauccugauauu....................................................................   5      0   smb
    ...................cuuuguaucccggauccugauauuU...................................................................   5      1   smb
    ....................uuuguaucccggauccugaua......................................................................   27     0   smb
    ....................uuuguauccUggauccugauau.....................................................................   1      1   smb
    ....................uuuguaucccggauccugauau.....................................................................   221    0   smb
    ....................uuuguaucccggauccugauauC....................................................................   1      1   smb
    ....................uuuguaucccggauccugauauu....................................................................   1      0   smb
    ........................uaucccggauccugauau.....................................................................   1      0   smb
    .........................................uuccucuucgcaaacgaug...................................................   1      0   smb
    ..............................................................ccaucaguggcgaagagA...............................   1      1   smb
    ..............................................................ccaucaguggcgaagagga..............................   16     0   smb
    ..............................................................ccaucaguggcgaagaggU..............................   1      1   smb
    ..............................................................ccaucaguggcgaagaggaa.............................   16     0   smb
    ..............................................................ccaucaguggcgaagaggaU.............................   3      1   smb
```

Mature Star

cuggucaucucugcauaaccuuuguaucccggauccugauauuccucuucgcaaacgaugagccaucaguggcgaagaggaauaucaagacccgggauauauauguuauuc			
....ccaucaguggcgaagaggaau............................	33	0	smb
....ccaucaguggcgaagaggaaua...........................	104	0	smb
....ccaucaguAgcgaagaggaaua...........................	1	1	smb
....ccaucaguggcgaagaggaauU...........................	13	1	smb
....ccaucaguggcgaagaggaauaA..........................	1	1	smb
....ccaucaguggcgaagaggaauau..........................	3	0	smb
....ccaucaguggcgaagaggaauauc.........................	2	0	smb
....ccaucaguggcgaagaggaauUuc.........................	1	1	smb
....ccaucaguggcgaagaggaauauU.........................	3	1	smb
....ccaucaguggcgaagaggaauauAa........................	2	1	smb
....caucaguggcgaagaggaauau..........................	1	0	smb
....aucaguggcgaagaggaauauc.........................	2	0	smb
....ggcgaagaggaauaucaagacc...................	1	0	smb
....aucaagacccgggauauaua........	1	0	smb
....ucaagacccgggauauau.........	2	0	smb
....ucaagacccgggauauauU........	1	1	smb
....ucaagacccgggauauaua........	92	0	smb
....ucaagacccgggauCuaua........	1	1	smb
....ucaagaGccgggauauaua........	1	1	smb
....ucaagacccgggauauauau.......	37	0	smb
....ucaagacccgggauauauGu.......	1	1	smb
....ucaUgacccgggauauauaug......	1	1	smb
....ucaagacccgggauauauauA......	1	1	smb
....ucaagacccgggauauauaug......	47	0	smb
....ucaagacUcgggauauauaugu.....	1	1	smb
....ucaagacccgggauauauaugu.....	490	0	smb
....ucaagacccgggauauauauAu.....	2	1	smb
....ucaagacccgggauauauaugA.....	1	1	smb
....ucGagacccgggauauauaugu.....	1	1	smb
....ucaagacccgggauauauauguu....	19	0	smb
....ucaagacccgggauauauauguuU...	2	1	smb
....ucaagacccgggauauauauguuUu..	1	1	smb
....caagacccgggauauauau.......	2	0	smb
....caagacccgggauauauauUu.....	1	1	smb
....caagacccgggauauauaugu.....	1	0	smb
....caagacccgggauauauauguA....	1	1	smb
....aagacccgggauauauaugu.....	2	0	smb
....gacccgggauauauaugu.....	1	0	smb

Provisional ID	:	3821647_95251_2334147_3711874+,...,1309910-_17674
Score total	:	102.8
Score for star read(s)	:	3.9
Score for read counts	:	94.4
Score for mfe	:	3.4
Score for randfold	:	1.6
Score for cons. seed	:	-0.6
Total read count	:	197
Mature read count	:	106
Loop read count	:	20
Star read count	:	71

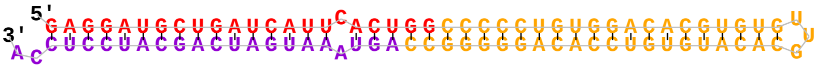

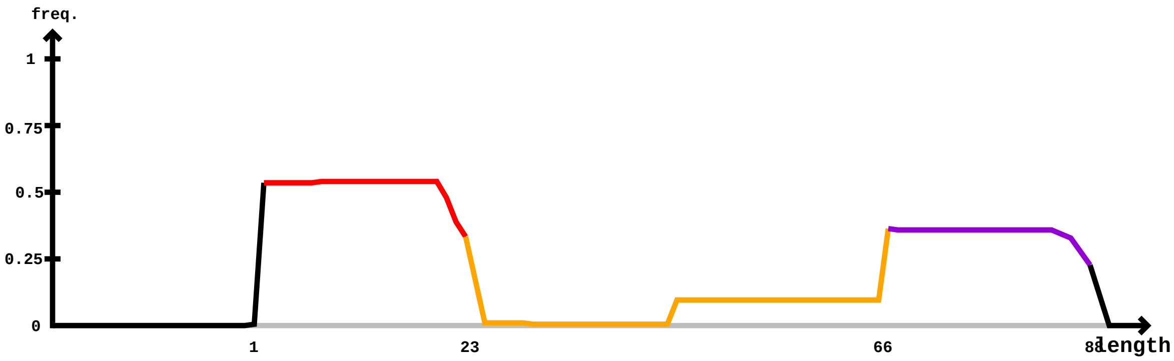

Mature **Star**

5'- ccugugucuggugucgugugGAGGAUGCUGAUCAUUCACUGG...			

5'- ccugugucuggugucgugug**gaggaugcugaucauucacugg**cccccuguggacacguguguugcacauguguccacagggggcc**aguaaaugaucagcauccucca**cacgg	-3'	obs	
ccugugucuggugucgugug**gaggaugcugaucauucacugg**cccccuguggacacguguguugcacauguguccacagggggccaguaaaugaucagcauccuccacacgg		exp	
..............(((((((((((((((((((((.(((((((((((((((((((((((((...))))))))))))))))))))))))).))))))))))))))))))))).	reads	mm	sample
...................ggaggaugcugaucauucacug...	1	0	smb
....................gaggaugcugaucauucac...	12	0	smb
....................gaggaugcugaucauucacu..	18	0	smb
....................gaggaugcugaucauucacug...	10	0	smb
....................gaggaugcugaucauucacugg..	45	0	smb
....................gaggaugcugaucauucacugU..	18	1	smb
....................gaggaugcugaucauucacuggA...	1	1	smb
....................gaggaugcugaucauucacuggU...	1	1	smb
..........................gcugaucauucacuggcccccu..	1	0	smb
....cccccuguggacacgugugu..	1	0	smb
....gcacauguguccacagggggcc...........................	18	0	smb
....gcacauguguccacagggggccU..........................	1	1	smb
....aguaaaugaucagcaucc.........	3	0	smb
....aguaaaugaucagcauccu........	3	0	smb
....aguaaaugaucagcauccuU.......	2	1	smb
....aguaaaugaucagcauccuc.......	8	0	smb
....aguaaaugaucagcauccucc......	10	0	smb
....aguaaaugaucagcauccucca.....	41	0	smb
....aguaaaugaucagcauccuccG.....	1	1	smb
....aguaaaugaucagcauccuccU.....	2	1	smb
....aguaaaugaucagcauccuccaU....	1	1	smb

Provisional ID	:	3820413_148141_4772246_3694007-,...,3789038+_15956
Score total	:	6625.4
Score for star read(s)	:	3.9
Score for read counts	:	6618.2
Score for mfe	:	2.3
Score for randfold	:	1.6
Score for cons. seed	:	-0.6
Total read count	:	12993
Mature read count	:	12833
Loop read count	:	0
Star read count	:	160

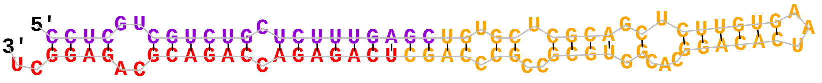

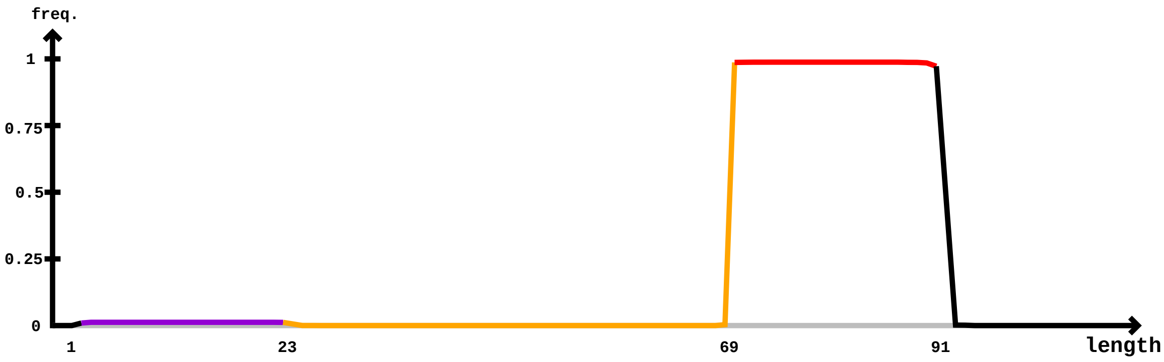

Star **Mature**

```
5'- gccucgucgucugcucuuugagcugugcucgcagcucuugugaaucacaggcacggugcgccgcccagcucagagaccagacgcagaggcuugcagcccucagcgcaaagg  -3'  obs
    gccucgucgucugcucuuugagcugugcucgcagcucuugugaaucacaggcacggugcgccgcccagcucagagaccagacgcagaggcuugcagcccucagcgcaaagg        exp
    (((((..((((((.(((((((((((.((.((((.(.((((((...))))))...).))))..)).))))))))))).))))))..))))).(((.((.....)))))....  reads  mm  sample
    .ccucgucgucugcucuuugag.........................................................................................  4      0   smb
    .ccucgucgucugcucuuugagc........................................................................................  119    0   smb
    .ccucgucgucugcucuuugagcuUu.....................................................................................  1      1   smb
    ..cucgucgucugcucuuugag.........................................................................................  2      0   smb
    ..cucgucgucugcucuuugagA........................................................................................  1      1   smb
    ..cucgucgucugcucuuugagc........................................................................................  11     0   smb
    ..cucgucgucugcucuuugagcu.......................................................................................  20     0   smb
    ..cucgucgucugcucuuugagcuU......................................................................................  1      1   smb
    ..cucgucgucugcucuuugagcuUu.....................................................................................  1      1   smb
    ....................................................................cucagagaccagacgcaga........................  2      0   smb
    ....................................................................cucUgagaccagacgcagaggc.....................  1      1   smb
    ....................................................................cucagagaccagacgcagaggc.....................  39     0   smb
    ....................................................................cucagagaccagacgcagaggUu....................  1      1   smb
    .....................................................................ucagagaccagacgcaga........................  7      0   smb
    .....................................................................ucagagaccagacgcagag.......................  3      0   smb
    .....................................................................ucagagaccagacgcagagg......................  24     0   smb
    .....................................................................ucagagaccagacgcagagU......................  1      1   smb
    .....................................................................ucagagaccagacgcagaggc.....................  115    0   smb
    .....................................................................ucagagaccagacgcagaggU.....................  1      1   smb
    .....................................................................ucagagGccagacgcagaggc.....................  1      1   smb
    .....................................................................ucagagacGagacgcagaggc.....................  1      1   smb
    .....................................................................ucagagaccagacUcagaggc.....................  1      1   smb
    .....................................................................ucagagaccagacCcagaggc.....................  1      1   smb
    .....................................................................ucagagaccUgacgcagaggc.....................  1      1   smb
    .....................................................................ucaUagaccagacgcagaggcu....................  1      1   smb
    .....................................................................ucUgagaccagacgcagaggcu....................  1      1   smb
    .....................................................................ucagagaccagacgcagaggcC....................  18     1   smb
    .....................................................................ucagagCccagacgcagaggcu....................  1      1   smb
    .....................................................................ucagagaccagacgUagaggcu....................  4      1   smb
    .....................................................................ucagaUaccagacgcagaggcu....................  1      1   smb
    .....................................................................ucagagaccagacgcagaggcu....................  12341  0   smb
    .....................................................................ucagagaccagacCcagaggcu....................  1      1   smb
    .....................................................................ucaCagaccagacgcagaggcu....................  1      1   smb
```

```
 Star                                                                  Mature
gccucgucgucugcucuuugagcugugcucgcagcucuugugaaucacaggcacggugcgccgcccagcucagagaccagacgcagaggcuugcagcccucagcgcaaagg
.....................................................................ucagagaccagacgcagagCcu....................    1     1     smb
.....................................................................ucagagaccagacgcagaggcG....................    158   1     smb
.....................................................................ucagagaccagacgcGgaggcu....................    1     1     smb
.....................................................................ucagagaccagacgcagCggcu....................    1     1     smb
.....................................................................ucagagaUcagacgcagaggcu....................    1     1     smb
.....................................................................ucagagaccagacgcagagAcu....................    1     1     smb
.....................................................................ucagagaccaAacgcagaggcu....................    1     1     smb
.....................................................................ucagagaccagacgcagGggcu....................    2     1     smb
.....................................................................ucagagaccagacgcUgaggcu....................    2     1     smb
.....................................................................ucagagGccagacgcagaggcu....................    2     1     smb
.....................................................................ucagagUccagacgcagaggcu....................    1     1     smb
.....................................................................Ccagagaccagacgcagaggcu....................    1     1     smb
.....................................................................ucagagaccagacUcagaggcu....................    8     1     smb
.....................................................................ucagagaccagacAcagaggcu....................    2     1     smb
.....................................................................ucagagacGagacgcagaggcu....................    1     1     smb
.....................................................................ucagagacUagacgcagaggcu....................    3     1     smb
.....................................................................ucagGgaccagacgcagaggcu....................    2     1     smb
.....................................................................ucagagaccUgacgcagaggcu....................    1     1     smb
.....................................................................ucagagaccagacgcagaggUu....................    2     1     smb
.....................................................................ucGgagaccagacgcagaggcu....................    5     1     smb
.....................................................................ucagagaccagacgcagaUgcu....................    1     1     smb
.....................................................................ucagagaccagacgcaCaggcu....................    2     1     smb
.....................................................................ucagagaccaUacgcagaggcu....................    1     1     smb
.....................................................................ucagagaccagacgcagaggcA....................    1     1     smb
.....................................................................ucagagaccagaUgcagaggcu....................    4     1     smb
.....................................................................ucagagaccagacgcaAaggcu....................    1     1     smb
.....................................................................ucagagaccagGcgcagaggcu....................    2     1     smb
.....................................................................ucagUgaccagacgcagaggcu....................    1     1     smb
.....................................................................ucagagaccagacgcagaggcuu...................    19    0     smb
.....................................................................ucGgagaccagacgcagaggcuu...................    1     1     smb
.....................................................................ucagagaccagacgcagaggcuug..................    5     0     smb
.....................................................................ucagagaccagacgcagaggcuugG.................    18    1     smb
......................................................................cagagaccagacgcagaggc.....................    1     0     smb
......................................................................cagagaccagacgcagaggcu....................    5     0     smb
......................................................................cagagaccagacgcagaggcuu...................    1     0     smb
.......................................................................agagaccagacgcagaggcu....................    5     0     smb
.........................................................................agaccagacgcagaggcu....................    1     0     smb
```

Provisional ID	:	3764071_8190_236910_5941_495556_175267_2079_27719403_9653088_8397
Score total	:	538
Score for star read(s)	:	3.9
Score for read counts	:	531.9
Score for mfe	:	2.8
Score for randfold	:	0
Score for cons. seed	:	-0.6
Total read count	:	1055
Mature read count	:	984
Loop read count	:	10
Star read count	:	61

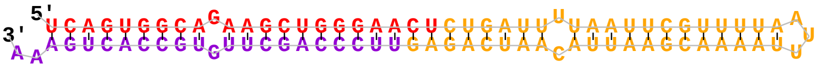

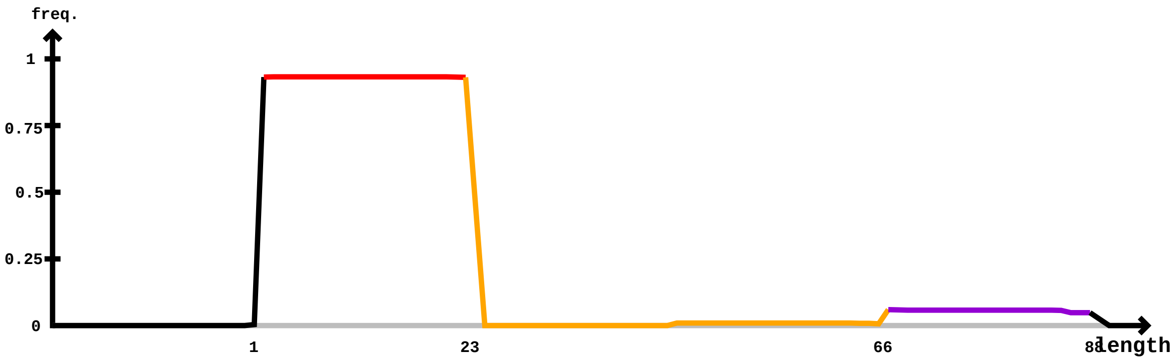

Mature   Star

```
5'- ugccaugccaaguagcugcuucaguggcagaagcugggaacucugauuuuaauucguuuuaauuuaaaacgaauuacaaucagaguucccagcuugugccacugaaacagcu  -3'   obs
    ugccaugccaaguagcugcuucaguggcagaagcugggaacucugauuuuaauucguuuuaauuuaaaacgaauuacaaucagaguucccagcuugugccacugaaacagcu         exp
    .............(((((.((((((((((.((((((((((((((((((.((((((((((((...)))))))))))).)))))))))))))))))).)))))))))).)))))  reads  mm  sample
    ...................uucaguggcagaagcugggaacu......................................................................  4      0   smb
    ....................ucaguggcagaagcugggaa........................................................................  1      0   smb
    ....................ucaguggcagaagcugggaac.......................................................................  1      0   smb
    ....................ucaguggcagaGgcugggaacu......................................................................  2      1   smb
    ....................ucUguggcagaagcugggaacu......................................................................  4      1   smb
    ....................ucaguggcagaagcCgggaacu......................................................................  1      1   smb
    ....................ucaguggcGgaagcugggaacu......................................................................  2      1   smb
    ....................ucaguggcUgaagcugggaacu......................................................................  1      1   smb
    ....................ucaguggcagGagcugggaacu......................................................................  1      1   smb
    ....................ucaguggcagaagcugggaacu......................................................................  962    0   smb
    ....................ucGguggcagaagcugggaacu......................................................................  1      1   smb
    ....................ucaguggcagaagcugggaacG......................................................................  1      1   smb
    ....................ucaguggcagaagcugggaacuU.....................................................................  2      1   smb
    .....................caguggcagaagcugggaacu......................................................................  1      0   smb
    ...............................................................uuaaaacgaauuacaauca..............................  1      0   smb
    ...............................................................uuaaaacgaauuacaaucaga............................  2      0   smb
    ...............................................................uuaaaacgaauuacaaucagag...........................  3      0   smb
    ...............................................................uuaaaacgaauuacaaucagaA...........................  1      1   smb
    ...............................................................uuaaaacgaauuacaaucagagu..........................  1      0   smb
    ...............................................................uuaaaacgaauuacaaucagaUu..........................  1      1   smb
    ...............................................................uuaaaacgaauuacaaucagagAu.........................  1      1   smb
    .....................................................................................uucccagcuugugccacu.........  1      0   smb
    .....................................................................................uucccagcuugugccacug........  9      0   smb
    .....................................................................................uucccagcuugugccacugaaU.....  1      1   smb
    .....................................................................................uucccagcuugugccacugaaa.....  47     0   smb
    .....................................................................................uucccagcuugugccacugaaC.....  1      1   smb
    .....................................................................................uucccagcuugugccacugaaaU....  1      1   smb
    ......................................................................................ucccagcuugugccacugaaac....  1      0   smb
```

Provisional ID	:	3820890_9358_73507_3700889+,3849N,3809352+_16662
Score total	:	2816.4
Score for star read(s)	:	3.9
Score for read counts	:	2808.2
Score for mfe	:	2.7
Score for randfold	:	1.6
Score for cons. seed	:	
Total read count	:	5520
Mature read count	:	3094
Loop read count	:	345
Star read count	:	2081

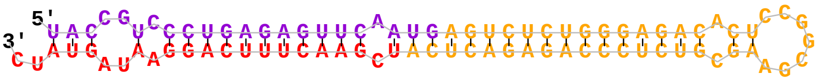

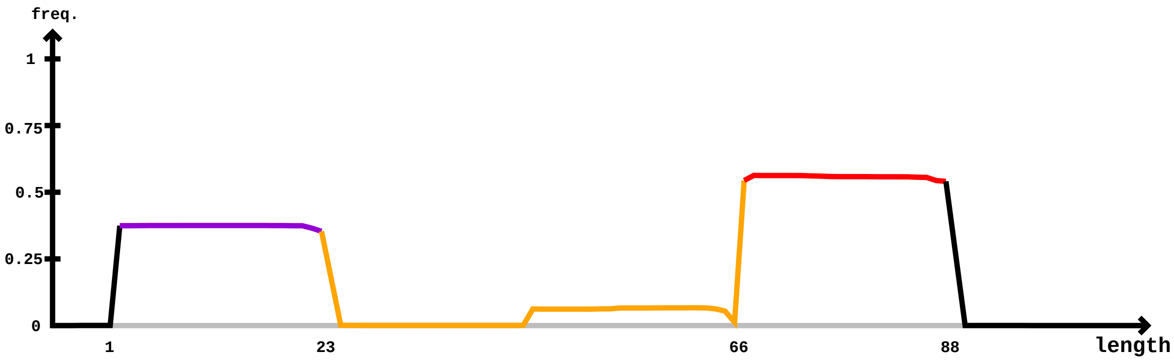

Star   Mature

```
5'- acggauaccgucccugagaguucaaugagucucugggagacacuccggcgaagcgucucccagagacucaucgaacuuucaggaauaguauccgucccggagguucuucgcc   -3'   obs
    acggauaccgucccugagaguucaaugagucucugggagacacuccggcgaagcgucucccagagacucaucgaacuuucaggaauaguauccgucccggagguucuucgcc         exp
    ((((((((..(.(((((((((((.(((((((((((((((((.((.......)).))))))))))))))))).))))))))))).)..))))))))..(((((...)))))..   reads   mm   sample
    .cggauaccgucccugagagu...........................................................................................   2       0    smb
    .....uaccgucccugagaguuc.........................................................................................   3       0    smb
    .....uaccgucccugagaguucaa.......................................................................................   51      0    smb
    .....uaccgucccugagaguucaaA......................................................................................   1       1    smb
    .....uaccgucccugagaguucaau......................................................................................   64      0    smb
    .....uaccgucccCgagaguucaaug.....................................................................................   1       1    smb
    .....uaccUucccugagaguucaaug.....................................................................................   1       1    smb
    .....uaccgucccugagaguucUaug.....................................................................................   1       1    smb
    .....Caccgucccugagaguucaaug.....................................................................................   1       1    smb
    .....uUccgucccugagaguucaaug.....................................................................................   1       1    smb
    .....uaccAucccugagaguucaaug.....................................................................................   3       1    smb
    .....uaccgucccugGgaguucaaug.....................................................................................   3       1    smb
    .....uaccgucccugagaguuUaaug.....................................................................................   1       1    smb
    .....uaccgucccugagaguucaauU.....................................................................................   5       1    smb
    .....uaccgucccugagagGucaaug.....................................................................................   1       1    smb
    .....Aaccgucccugagaguucaaug.....................................................................................   1       1    smb
    .....uaUcgucccugagaguucaaug.....................................................................................   1       1    smb
    .....uaccgucccugagaUuucaaug.....................................................................................   1       1    smb
    .....uaccgucccugagaguucaaGg.....................................................................................   1       1    smb
    .....uaccgucccugUgaguucaaug.....................................................................................   2       1    smb
    .....uaccgucccugagaguucaaug.....................................................................................   1876    0    smb
    .....uaccgAcccugagaguucaaug.....................................................................................   2       1    smb
    .....uaccgucccugagaguucaauC.....................................................................................   1       1    smb
    .....uaccgucccugagaguucaauA.....................................................................................   1       1    smb
    .....uaccgucccugagaguucaaugC....................................................................................   1       1    smb
    .....uaccgucccugagaguucaauga....................................................................................   3       0    smb
    .....uaccgucccugagaguucaaugU....................................................................................   48      1    smb
    .....uaccgucccugagaguucaaugaA...................................................................................   2       1    smb
    .......ccgucccugagaguucaauU.....................................................................................   1       1    smb
    ........cgucccugagaguucaaug.....................................................................................   2       0    smb
    ...........ccugagaguucaaugaguc..................................................................................   1       0    smb
    ...........................agucucugggagacacuccggc...............................................................   3       0    smb
    ...............................................gcgaagcgucucccagagacuc...........................................   1       0    smb
```

Star **Mature**

acggauaccgucccugagaguucaaugagucucugggagacacuccggcgaagcgucucccagagacucaucgaacuuucaggaauaguauccgucccggagguucuucgcc			
....cgaagcgucucccagagU..	1	1	smb
....cgaagcgucucccagaga..	2	0	smb
....cgaagcgucucccaUagac...	1	1	smb
....cgaagcgucucccagagac...	17	0	smb
....cgaagcgucucccagagaU...	1	1	smb
....cgaagcgucucccagagacu..	48	0	smb
....cgaagcgucucccagaUacuc...	2	1	smb
....cgaagcgucucccagagacuc...	225	0	smb
....cgaagcgucucccagagacuU...	4	1	smb
....cgaagcgucucccagagacuca..	33	0	smb
....cgaagcgucucccagagacucU..	5	1	smb
....cgaagcgucucccagagacucauU..	2	1	smb
....ucucccagagacucaucgaac....................................	1	0	smb
....ucucccagagacucaucgaacu...................................	3	0	smb
....ucucccagagacucaucgaacuuucaggaaua.........................	1	0	smb
....cucccagagacucaucgaacuu..................................	1	0	smb
....ucccagagacucaucgaacu...................................	4	0	smb
....ucccagagacucaucgaacuu..................................	3	0	smb
....ucccagagacucaucgaacuuu.................................	8	0	smb
....ucccagagacucaucgaacuuuU................................	1	1	smb
....cccagagacucaucgaacuu..................................	1	0	smb
....cccagagacucaucgaacuuuc................................	1	0	smb
....agagacucaucgaacuuucagg.............................	1	0	smb
....agagacucaucgaacuuucaggaauagu.......................	1	0	smb
....gagacucaucgaacuuucagga............................	1	0	smb
....acucaucgaacuuucagga............................	1	0	smb
....caucgaacuuucaggaauaguau.....................	1	0	smb
....caucgaacuuucaggaauaguauccgucccgg............	1	0	smb
....ucgaacuuucaggaauag........................	8	0	smb
....ucgaacuuucaggaauagu.......................	4	0	smb
....ucgaacuuucaCgaauagua......................	1	1	smb
....ucgaacuuucaggaauagua......................	63	0	smb
....ucgaacuuucaggaauaguU......................	1	1	smb
....ucgaacuuucaggaauaguau.....................	14	0	smb
....ucgaacuuucaggaauaguCu.....................	1	1	smb
....ucgaaUuuucaggaauaguauc....................	1	1	smb
....ucgaacuuucaggaauaAuauc....................	1	1	smb
....ucgaacuuucaggaGuaguauc....................	1	1	smb
....ucgaacuCucaggaauaguauc....................	1	1	smb
....ucgaacuuuUaggaauaguauc....................	1	1	smb
....ucUaacuuucaggaauaguauc....................	1	1	smb
....ucgaacuuucaggaauUguauc....................	6	1	smb
....ucgaacuuucaggaauaguUuc....................	2	1	smb
....ucgaacuuucaggaauaguauc....................	2618	0	smb
....ucgGacuuucaggaauaguauc....................	1	1	smb
....Ccgaacuuucaggaauaguauc....................	1	1	smb
....ucgaacuuucaggaauaUuauc....................	3	1	smb
....ucgaacuuucaggaauagCauc....................	1	1	smb
....ucgaacuuucaUgaauaguauc....................	2	1	smb
....ucgaacuuucGggaauaguauc....................	2	1	smb
....ucgaacuuucaggaaAaguauc....................	1	1	smb
....ucgaacuuucaggGauaguauc....................	1	1	smb
....ucgaacuuucaggaauGguauc....................	1	1	smb
....ucAaacuuucaggaauaguauc....................	1	1	smb
....ucgaCcuuucaggaauaguauc....................	1	1	smb
....ucgaacuuucaggaauaguaucU...................	217	1	smb
....ucgaacuuucaggaauaguaucA...................	1	1	smb
....ucgaacuuucaggaauaguaucc...................	31	0	smb
....cgaacuuucagCaauaguaucc...................	1	1	smb
....cgaacuuucaggaauaguaucc...................	104	0	smb

Provisional ID	:	3818239_11905_99070_3179782+,...,3760891+_13197
Score total	:	3046.9
Score for star read(s)	:	3.9
Score for read counts	:	3038.7
Score for mfe	:	3.3
Score for randfold	:	1.6
Score for cons. seed	:	-0.6
Total read count	:	5972
Mature read count	:	5409
Loop read count	:	151
Star read count	:	412

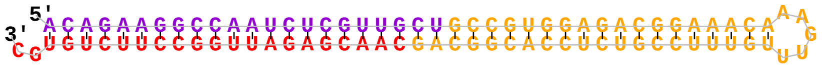

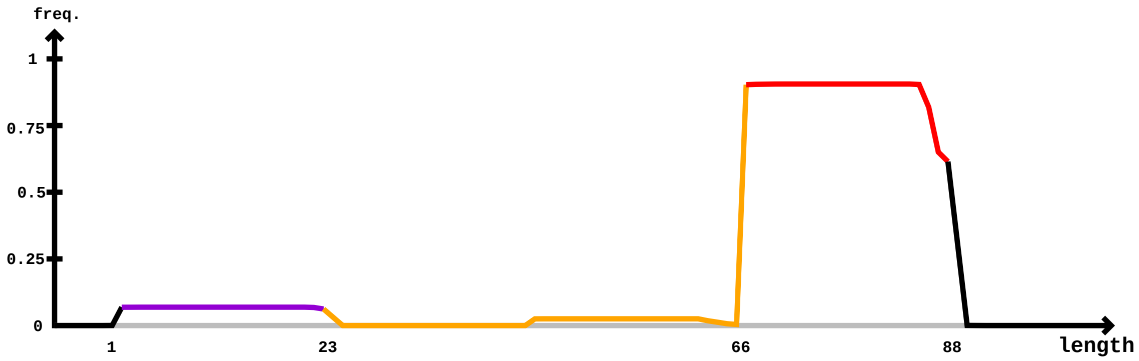

Star **Mature**

```
5'- aaagcacagaaggccaaucucguugcugccguggagacggaaacaaaguuuguuuccgucuccacggcagcaacgagauuggccuucugugcuuuggacauugaaaca  -3'  obs
    aaagcacagaaggccaaucucguugcugccguggagacggaaacaaaguuuguuuccgucuccacggcagcaacgagauuggccuucugugcuuuggacauugaaaca        exp
    (((((((((((((((((((((((((((((((((((((((((((((.....))))))))))))))))))))))))))))))))))))))))))))).............   reads   mm   sample
    ....cacagaaggccaaucucguug...................................................................................   1       0    smb
    ....cacagaaggccaaucucguugc..................................................................................   1       0    smb
    .....acagaaggccaaucucguug...................................................................................   5       0    smb
    .....acagaaggccaaucucguugU..................................................................................   3       1    smb
    .....acagaaggccaaucucguugc..................................................................................   30      0    smb
    .....acagaagCccaaucucguugcu.................................................................................   1       1    smb
    .....acagaaggccaaucucguugcC.................................................................................   1       1    smb
    .....acagaaggccaaucucguugcu.................................................................................   366     0    smb
    .....acagaaggccaaucuUguugcu.................................................................................   1       1    smb
    .....acagaaggccaaucucguugcG.................................................................................   1       1    smb
    ......cagaaggccaaucucguuU...................................................................................   1       1    smb
    .......agaaggccaaucucguugcu.................................................................................   1       0    smb
    ................................................uuuguuuccgucuccacg..........................................   42      0    smb
    ................................................uuuguuuccgucCccacg..........................................   1       1    smb
    ................................................uuuguuuccgucuccacgU.........................................   7       1    smb
    ................................................uuuguuuccgucuccacgg.........................................   26      0    smb
    ................................................uuuguuuccgucuccacggU........................................   1       1    smb
    ................................................uuuguuuccgucuccacggc........................................   33      0    smb
    ................................................uuuguuuccgucuccacggcU.......................................   3       1    smb
    ................................................uuuguuuccgucuccacggca.......................................   13      0    smb
    ................................................uuuguuuccgucuccacggcag......................................   20      0    smb
    ................................................uuuguuuccgucuccacggcaU......................................   1       1    smb
    ................................................uuuguuuccgucuccacggcagU.....................................   3       1    smb
    .................................................uuguuuccgucuccacggcag......................................   1       0    smb
    ......................................................................caacgagauuggccuucu....................   11      0    smb
    ......................................................................caacgagauuAgccuucug...................   1       1    smb
    ......................................................................caacgagauuggccuuGug...................   1       1    smb
    ......................................................................caacgagauuggccuucug...................   496     0    smb
    ......................................................................caacgagauuggccuucuU...................   10      1    smb
    ......................................................................caacgagaCuggccuucugu..................   1       1    smb
    ......................................................................caacUagauuggccuucugu..................   1       1    smb
    ......................................................................caacgagauuggcGuucugu..................   1       1    smb
    ......................................................................caacgagauuggcUuucugu..................   1       1    smb
```

Star **Mature**

aaagcacagaaggccaaucucguugcugccguggagacggaaacaaaguuuguuuccgucuccacggcagcaacgagauuggccuucugugcuuuggacauugaaaca			
....caacgagauuggccuucCgu..................	1	1	smb
....caacgagauuggccuucuUu..................	10	1	smb
....caacgagauuggccuucugu..................	985	0	smb
....caacgagauuggGcuucugu..................	1	1	smb
....caacgaUauuggccuucugu..................	1	1	smb
....caacgagauuggccuucuguU.................	72	1	smb
....caacgagauuggccuucuguA.................	6	1	smb
....caacgagauuggccuucugug.................	134	0	smb
....caaUgagauuggccuucugug.................	1	1	smb
....caacgagauuggccuucugCgc................	2	1	smb
....caacgagauuggccuucCgugc................	4	1	smb
....caacgagauugAccuucugugc................	3	1	smb
....caacgagauuggccuucuAugc................	1	1	smb
....caacgagauuggccuucuguUc................	5	1	smb
....caacgagauuggccuucugugG................	4	1	smb
....caacgagauuggccuucugugA................	5	1	smb
....caacgagauuggccuuUugugc................	1	1	smb
....caacgagauuggccuucuguAc................	3	1	smb
....caacgagauugCccuucugugc................	2	1	smb
....caaUgagauuggccuucugugc................	2	1	smb
....caacgagauuCgccuucugugc................	1	1	smb
....caacgagauuAgccuucugugc................	1	1	smb
....caacgagauuggccuucugugc................	3595	0	smb
....caacgaAauuggccuucugugc................	1	1	smb
....caacgagauuggccuucugugU................	19	1	smb
....caacgagGuuggccuucugugc................	1	1	smb
....caacgagauugUccuucugugc................	2	1	smb
....caacgagauuggccuucugugcu...............	2	0	smb
....caacgagauuggccuucugugcuu..............	1	0	smb
....caacgagauuggccuucugugUuu..............	1	1	smb
....caacgagauuggccuucugugUuuu.............	1	1	smb
....aacgagauuggccuucug...................	1	0	smb
....aacgagauuggccuucugu..................	1	0	smb
....aacgagauuggccuucugugc................	4	0	smb
....aacgagauuggccuucugugcu...............	5	0	smb
....acgagauuggccuucugu..................	1	0	smb
....acgagauuggccuucugugc................	2	0	smb
....cgagauuggccuucugugc................	3	0	smb
....gagauuggccuucugugc................	1	0	smb

Provisional ID	:	3828564_91305_3563970_3809624+,...,3757318+_27360
Score total	:	1378.7
Score for star read(s)	:	3.9
Score for read counts	:	1371
Score for mfe	:	2.8
Score for randfold	:	1.6
Score for cons. seed	:	-0.6
Total read count	:	2701
Mature read count	:	2037
Loop read count	:	3
Star read count	:	661

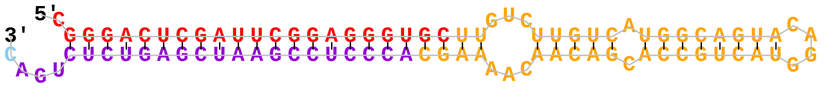

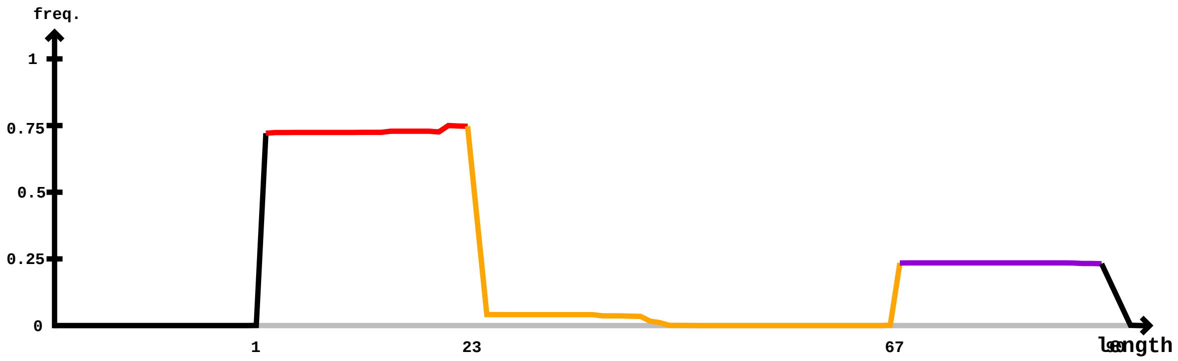

Mature Star

```
5'- uuucauggacuacgggcgugcgggacucgauucggagggugcuugucuugucauggcaguacagguacugccacgacaacaaaagcacccuccgaaucgagucucugacacc  -3'   obs
    uuucauggacuacgggcgugcgggacucgauucggagggugcuugucuugucauggcaguacagguacugccacgacaacaaaagcacccuccgaaucgagucucugacacc          exp
    .........(....)..(((((((((((((((((((((((((((...(((((.((((((((....)))))))).)))))...))))))))))))))))))))).))).))).   reads   mm   sample
    ...................gcgggacucgauucggagggugc......................................................................   1       0    smb
    ....................cgggacucgauucggagA..........................................................................   2       1    smb
    ....................cgggacucgauucggagg..........................................................................   5       0    smb
    ....................cgggacucgauucggagU..........................................................................   3       1    smb
    ....................cgggacucgauucggaggg.........................................................................   29      0    smb
    ....................cgggacucgauucggagggA........................................................................   1       1    smb
    ....................cgggacucgauucggagggu........................................................................   4       0    smb
    ....................cgggacucgauucggagggug.......................................................................   3       0    smb
    ....................cgggaUucgauucggagggugc......................................................................   1       1    smb
    ....................cgggacucgauAcggagggugc......................................................................   1       1    smb
    ....................cgggacucgauCcggagggugc......................................................................   1       1    smb
    ....................cgggacucgUuucggagggugc......................................................................   1       1    smb
    ....................cggUacucgauucggagggugc......................................................................   2       1    smb
    ....................cgggacAcgauucggagggugc......................................................................   1       1    smb
    ....................cgggacucgauucggagggugc......................................................................   1963    0    smb
    ....................cgggacucgauucggGgggugc......................................................................   2       1    smb
    ....................Agggacucgauucggagggugc......................................................................   1       1    smb
    ....................cgggacucgauucggaggguAc......................................................................   1       1    smb
    ....................cgggacucgauucggagggugU......................................................................   1       1    smb
    ....................cgggacucgauGcggagggugc......................................................................   1       1    smb
    ....................cgggacucgauucggagggugcu.....................................................................   3       0    smb
    ....................cgggacucgauucggagggugcA.....................................................................   1       1    smb
    ....................cgggacucgauucggagggugcuGg...................................................................   1       1    smb
    .....................gggacucgauucggagggugc......................................................................   6       0    smb
    .....................gggacucgauucggagggugcu.....................................................................   1       0    smb
    .......................gacucgauucggagggugc......................................................................   1       0    smb
    ..............................uucggagggugcuugucuugucaug.........................................................   1       0    smb
    .................................ggagggugcuugucuugucauU.........................................................   1       1    smb
    .................................ggagggugcuugucuugucaug.........................................................   10      0    smb
    .................................ggagggugcuugucuugucaugg........................................................   1       0    smb
    ......................................gugcuugucuCgucauggcagua...................................................   2       1    smb
    .......................................ugcuugucuCgucauggca......................................................   3       1    smb
    .......................................ugcuugucuCgucauggcag.....................................................   2       1    smb
```

```
                    Mature                                                        Star
uuucauggacuacgggcgugcgggacucgauucggagggugcuugucuugucauggcaguacagguacugccacgacaacaaaagcacccuccgaaucgagucucugacacc
.......................................ugcuugucuCgucauggcagu....................................................    51    1    smb
.......................................ugcuugucuCgucauggcagua...................................................    13    1    smb
.......................................ugcuugucuCgucauggcaguac..................................................    28    1    smb
..........................................uugucuugucauggcaguac..................................................    1     0    smb
..........................................uugucGugucauggcaguaca.................................................    1     1    smb
..........................................uugucuugucauggcaguacagU...............................................    1     1    smb
.....................................................................................cacccuccgaaucgagucucuga....    3     0    smb
......................................................................................acccuccgaaucgagucu........    1     0    smb
......................................................................................acccuccgaaucgagucuc.......    5     0    smb
......................................................................................acccuccgaaucgagucucug.....    3     0    smb
......................................................................................acccuccgaaucgagucucuUa....    1     1    smb
......................................................................................acccuccgaaucgagucucugU....    2     1    smb
......................................................................................acccuccgaaucgagucucuga....    618   0    smb
......................................................................................acccuccgaaucgagucucugac...    1     0    smb
......................................................................................acccuccgaaucgagucucugaA...    3     1    smb
......................................................................................acccuccgaaucgagucucugaU...    16    1    smb
......................................................................................acccuccgaaucgagucucugaca..    4     0    smb
......................................................................................acccuccgaaucgagucucugacaA.    1     1    smb
......................................................................................acccuccgaaucgagucucugacaU.    2     1    smb
.......................................................................................cccuccgaaucgagucucugac...    1     0    smb
```

Provisional ID	:	2746967_287_3957_128_9588_1213_129_61353_20133_4198
Score total	:	73.9
Score for star read(s)	:	3.9
Score for read counts	:	65.9
Score for mfe	:	3.1
Score for randfold	:	1.6
Score for cons. seed	:	-0.6
Total read count	:	141
Mature read count	:	129
Loop read count	:	3
Star read count	:	9

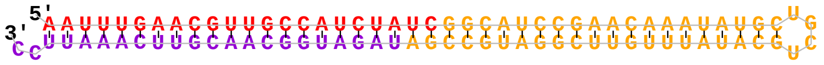

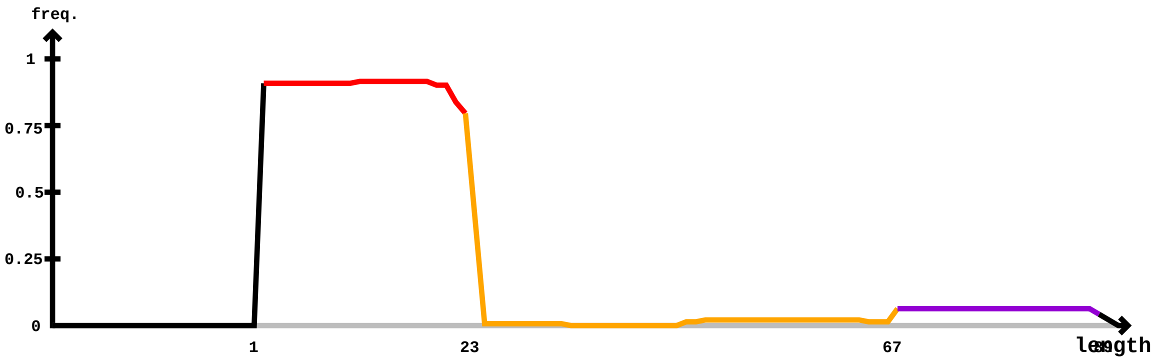

Mature   Star

```
5'- ugugguagaagcauacaggaauuugaacguugccaucuaucggcauccgaacaaauaugcugcugcauauuuguucggaugccgauagauggcaacguucaaauuccug  -3'    obs
    ugugguagaagcauacaggaauuugaacguugccaucuaucggcauccgaacaaauaugcugcugcauauuuguucggaugccgauagauggcaacguucaaauuccug          exp
    ...............(((((((((((((((((((((((((((((((((((((((((((((....)))))))))))))))))))))))))))))))))))))))))))))    reads      mm       sample
    ...................aauuugaacguugccauc........................................................................    2          0        smb
    ...................aauuugaacguugccaucua......................................................................    8          0        smb
    ...................aauuugaacguugccUucua......................................................................    1          1        smb
    ...................aauuugaacguugccaucuau.....................................................................    6          0        smb
    ...................aauuugaacguugccaucuauc....................................................................    107        0        smb
    ...................aauuugaacguugccaucuauG....................................................................    1          1        smb
    ...................aauuugaacguugccaucuaucU...................................................................    4          1        smb
    .............................uugccaucuaucggcauccgaa..........................................................    1          0        smb
    ...............................................................ugcauauuuguucggaugc...........................    1          0        smb
    ...............................................................Cgcauauuuguucggaugccga........................    1          1        smb
    .................................................................cauauuuguucggaugccga........................    1          0        smb
    .....................................................................................uagauggcaacguucaaauuc...    3          0        smb
    .....................................................................................uagauggcaacguucaaauucc..    6          0        smb
```

Provisional ID	:	3822106_68578_1601548_3718911+,...,3796014-_18578
Score total	:	595.5
Score for star read(s)	:	3.9
Score for read counts	:	587.4
Score for mfe	:	3.2
Score for randfold	:	1.6
Score for cons. seed	:	-0.6
Total read count	:	1164
Mature read count	:	1136
Loop read count	:	0
Star read count	:	28

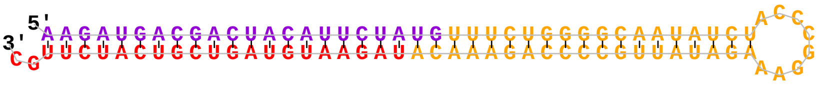

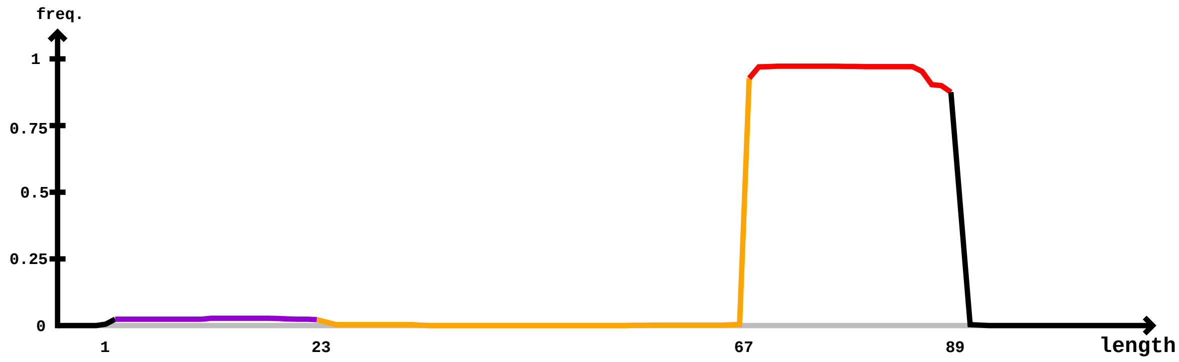

Star **Mature**

```
5'- gagcaagaugacgacuacauucuauguuucuggggcaauaucuacccggaaagauauugccccagaaacauagaauguagucgucaucuugcuccgaggcgguaccucggucc -3'   obs
    gagcaagaugacgacuacauucuauguuucuggggcaauaucuacccggaaagauauugccccagaaacauagaauguagucgucaucuugcuccgaggcgguaccucggucc        exp
    .((((((((((((((((((((((((((((((((((((((((((........))))))))))))))))))))))))))))))))))))))))))((((((.....))))))..   reads   mm   sample
    ...caagaugacgacuacauu...........................................................................................   1       0    smb
    ...caagaugacgacuacauuc..........................................................................................   2       0    smb
    ...caagaugacgacuacauucu.........................................................................................   1       0    smb
    ...caagaugacgacuacauucuau.......................................................................................   2       0    smb
    ....aagaugacCacuacauucuaug......................................................................................   1       1    smb
    ....aagaugacgacuacauucuaug......................................................................................   20      0    smb
    ....aagaugacgacuacauucuaugu.....................................................................................   1       0    smb
    ..............cuacauucuauguuucuggggc............................................................................   3       0    smb
    ..............cuacauucuauguuucuggggcU...........................................................................   1       1    smb
    ..........................................................gccccagaaacauagaauguag................................   1       0    smb
    ............................................................cccagaaacauagaauguaguc..............................   1       0    smb
    ....................................................................cauagaauguagucgucaucu.......................   1       0    smb
    .....................................................................auagaauguagucgucaucu.......................   1       0    smb
    .....................................................................Cuagaauguagucgucaucuugc....................   1       1    smb
    ......................................................................uagaauguagucgucauc........................   20      0    smb
    ......................................................................uagaauCuagucgucauc........................   1       1    smb
    ......................................................................uagaauguagucgucaucu.......................   54      0    smb
    ......................................................................uagaauguagucgucauUuu......................   1       1    smb
    ......................................................................uagaauguagucgucaucuu......................   3       0    smb
    ......................................................................uagaauguagucgucaucuug.....................   23      0    smb
    ......................................................................uagaauguaAucgucaucuug.....................   1       1    smb
    ......................................................................uagaauguagucgucaucuuU.....................   5       1    smb
    ......................................................................uagaauguaguUgucaucuugc....................   1       1    smb
    ......................................................................Cagaauguagucgucaucuugc....................   1       1    smb
    ......................................................................uagaauguagucgucaCcuugc....................   1       1    smb
    ......................................................................uagaauguagucgucaucuugc....................   931     0    smb
    ......................................................................uagaauguagucgucaucuugG....................   1       1    smb
    ......................................................................uagaauguGgucgucaucuugc....................   2       1    smb
    ......................................................................uagaaugAagucgucaucuugc....................   1       1    smb
    ......................................................................uagaauguagucgucaucuugU....................   6       1    smb
    ......................................................................uagaauguagucgucaucuugA....................   1       1    smb
    ......................................................................uagaaugCagucgucaucuugc....................   1       1    smb
    ......................................................................uagaauguagucgucaucuugAu...................   1       1    smb
```

```
    Star                                                              Mature
gagcaagaugacgacuacauucuauguuucuggggcaauaucuacccggaaagauauugccccagaaacauagaauguagucgucaucuugcuccgaggcgguaccucgguc
......................................................................uagaauguagucgucaucuugcu...................    23    0    smb
......................................................................uagaauguagucgucaucuugcuA..................    1     1    smb
......................................................................uagaauguagucgucaucuugcuU..................    1     1    smb
.......................................................................agaauguagucgucaucu.......................    2     0    smb
.......................................................................agaauguagucgucaucuugc....................    10    0    smb
.......................................................................agGauguagucgucaucuugcu...................    1     1    smb
.......................................................................agaauguagucgucaucuugcu...................    37    0    smb
........................................................................gaauguagucgucaucuugcu...................    1     0    smb
.........................................................................aauguagucgucaucuugcucc.................    2     0    smb
```

Provisional ID	:	3822019_126068_4805016_3717763-,...,3729016+_18282
Score total	:	179.9
Score for star read(s)	:	3.9
Score for read counts	:	171.9
Score for mfe	:	3.1
Score for randfold	:	1.6
Score for cons. seed	:	-0.6
Total read count	:	349
Mature read count	:	231
Loop read count	:	2
Star read count	:	116

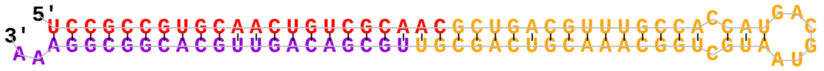

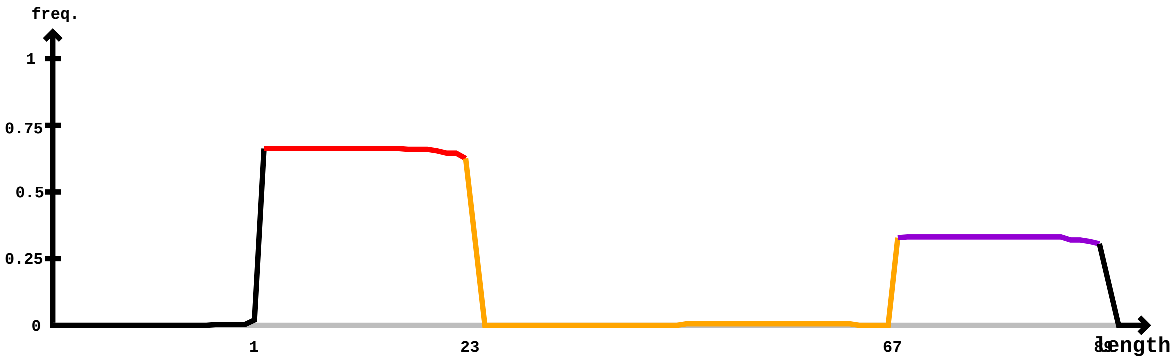


```
                    Mature                                                            Star
5'- uccaaaucugcaucaagcuuuccgccgugcaacugucgcaacgcugacguuugccaccaugacguaaugcuggcaaacgucagcguugcgacaguugcacggcggaaacccu  -3'   obs
    uccaaaucugcaucaagcuuuccgccgugcaacugucgcaacgcugacguuugccaccaugacguaaugcuggcaaacgucagcguugcgacaguugcacggcggaaacccu        exp
    ..................((((((((((((((((((((((((((((((((((((((.(((......))).))))))))))))))))))))))))))))))))))))))....   reads    mm    sample
    ...............agcuuuccgccgugUaacug.............................................................................   1        1     smb
    ...................uuccgccgugcaacugucg..........................................................................   1        0     smb
    ...................uuccgccgugcaacugucgcaa.......................................................................   4        0     smb
    ...................uuccgccgugcaacugucgcaaU......................................................................   1        1     smb
    ....................uccgccgugcaacugucg..........................................................................   1        0     smb
    ....................uccgccgugcaacugucgc.........................................................................   3        0     smb
    ....................uccgccgugcaacugucgcaa.......................................................................   3        0     smb
    ....................uccgccgugcaacugucgcaac......................................................................   200      0     smb
    ....................uccAccgugcaacugucgcaac......................................................................   1        1     smb
    ....................uccgccgugcaacugucgcaaU......................................................................   1        1     smb
    ....................uccgAcgugcaacugucgcaac......................................................................   1        1     smb
    ....................uccgccgugcaacugucgcaacU.....................................................................   15       1     smb
    ................................................................uaaugcuggcaaacguca..............................   2        0     smb
    ......................................................................................ugcgacaguugcacggcg........   4        0     smb
    ......................................................................................ugcgacaguugcacggcgga......   2        0     smb
    ......................................................................................ugcgacaguugcacggcggaa.....   2        0     smb
    ......................................................................................ugcgacaguugcacggcggaU.....   1        1     smb
    ......................................................................................ugcgacaguugcacggcggaaa....   104      0     smb
    ......................................................................................ugcgacaguugcacggcggaaaU...   2        1     smb
    .......................................................................................gcgacaguugcacggcggaaac...   1        0     smb
```

Provisional ID	:	3816686_121257_3582422_1491058+,...,3738891-_11659
Score total	:	686.4
Score for star read(s)	:	3.9
Score for read counts	:	678.7
Score for mfe	:	2.8
Score for randfold	:	1.6
Score for cons. seed	:	-0.6
Total read count	:	1343
Mature read count	:	956
Loop read count	:	365
Star read count	:	22

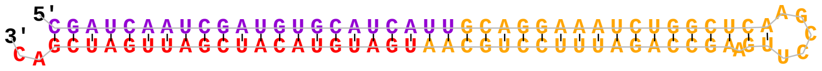

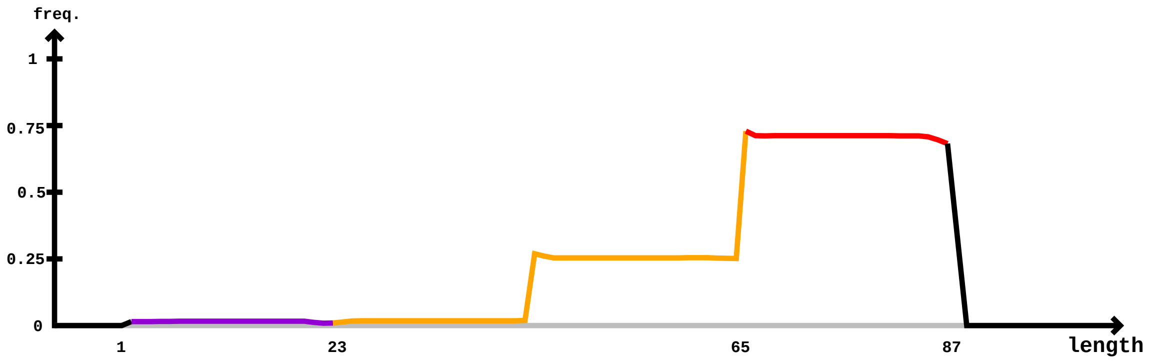

Star Mature

```
5'- aucagucgaucaaucgaugugcaucauugcaggaaaucuggcucaagccuugaagccagauuuccugcaaugauguacaucgauugaucgaccguuugauugacugguu   -3'    obs
    aucagucgaucaaucgaugugcaucauugcaggaaaucuggcucaagccuugaagccagauuuccugcaaugauguacaucgauugaucgaccguuugauugacugguu          exp
    (((((((((((((((((((((((((((((((((((((((((((((.....)).)))))))))))))))))))))))))))))))))))))))....)))).........   reads   mm   sample
    ......cgaucaaucgaugugcauc....................................................................................   6       0    smb
    ......cgaucaaucgaugugcauca...................................................................................   4       0    smb
    ......cgaucaaucgaugugcaucau..................................................................................   2       0    smb
    ......cgaucaaucgaugugcaucauu.................................................................................   8       0    smb
    .........ucaaucgaugugcaucau..................................................................................   1       0    smb
    ...........aaucgaugugcaucauuU................................................................................   1       1    smb
    ..........................ugcaggaaaucuggcucaagU..............................................................   1       1    smb
    ..........................ugcaggaaaucuggcucaagcc.............................................................   2       0    smb
    ..........................ugcaggaaaucuggcucaagcU.............................................................   1       1    smb
    ............................gcaggaaaucuggcucaagc.............................................................   2       0    smb
    ............................gcaggaaaucuggcucaagcc............................................................   5       0    smb
    ............................gcaggaaaucuggcucaagcU............................................................   4       1    smb
    ............................gcaggaaaucuggcucaagccu...........................................................   6       0    smb
    ............................gcaggaaaucuggcucaagcUu...........................................................   2       1    smb
    ..............................aggaaaucuggcucaagccu...........................................................   1       0    smb
    ...............................................ccuugaagccagauuuccugcaa.......................................   2       0    smb
    ................................................cuugaagccagauuuccug..........................................   2       0    smb
    ................................................cuugaagccagauuuccugc.........................................   1       0    smb
    ................................................cuugaagccagauuuccugca........................................   1       0    smb
    ................................................cuugaagccCgauuuccugcaa.......................................   1       1    smb
    ................................................cuugaaUccagauuuccugcaa.......................................   1       1    smb
    ................................................Guugaagccagauuuccugcaa.......................................   1       1    smb
    ................................................cuugaagccagauuuccugcaa.......................................   300     0    smb
    ................................................cuugaagccagauuuccugUaa.......................................   1       1    smb
    ................................................cuugaagccagauuuccugcaC.......................................   1       1    smb
    ................................................cuugaGgccagauuuccugcaa.......................................   1       1    smb
    ................................................cuugaagcAagauuuccugcaa.......................................   1       1    smb
    ................................................cuugaagccagauuuccugcaau......................................   23      0    smb
    ................................................cuugaagccagauuuccugcaaA......................................   2       1    smb
    ................................................Uuugaagccagauuuccugcaau......................................   1       1    smb
    ................................................cuugaagccagauuuGcugcaau......................................   1       1    smb
    ................................................cuugaagccagauuuccugcaauU.....................................   1       1    smb
    ................................................................cugcaaugauguacaucgauug........................   1       0    smb
```

```
      Star                                                                Mature
aucagucgaucaaucgaugugcaucauugcaggaaaucuggcucaagccuugaagccagauuuccugcaaugauguacaucgauugaucgaccguuugauugacugguu
......................................................................ugauguacaucgauugauc....................    5     0     smb
......................................................................ugauguacaucgauugaucg...................    15    0     smb
......................................................................ugauguacaucgauugaucgU..................    4     1     smb
......................................................................ugauguacaucgauugaucga..................    15    0     smb
......................................................................ugauguacaucgauugaucgaU.................    49    1     smb
......................................................................ugauAuacaucgauugaucgac.................    1     1     smb
......................................................................ugauguacGucgauugaucgac.................    1     1     smb
......................................................................ugauguacCucgauugaucgac.................    1     1     smb
......................................................................ugauguacaucgauugaucgac.................    611   0     smb
......................................................................ugauguacaucgUuugaucgac.................    1     1     smb
......................................................................ugauguCcaucgauugaucgac.................    1     1     smb
......................................................................Cgauguacaucgauugaucgac.................    1     1     smb
......................................................................ugauguacaucgauugaucgacU................    242   1     smb
......................................................................ugauguacaucgauugaucgacc................    2     0     smb
......................................................................ugauguacaucgauugaucgacA................    1     1     smb
......................................................................ugauguacaucgauugaucgacG................    1     1     smb
.......................................................................gauguacaucgauugaucgacc................    4     0     smb
.........................................................................uguacaucgauugaucgacc................    1     0     smb
```

Provisional ID	:	3820624_28451_573315_3696649+,...,3731600-_16317
Score total	:	2.6
Score for star read(s)	:	-1.3
Score for read counts	:	0
Score for mfe	:	2.9
Score for randfold	:	1.6
Score for cons. seed	:	-0.6
Total read count	:	1143
Mature read count	:	608
Loop read count	:	393
Star read count	:	142

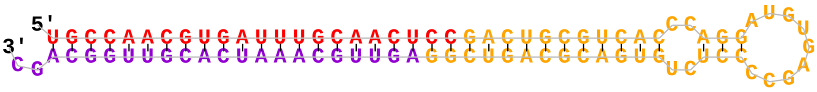

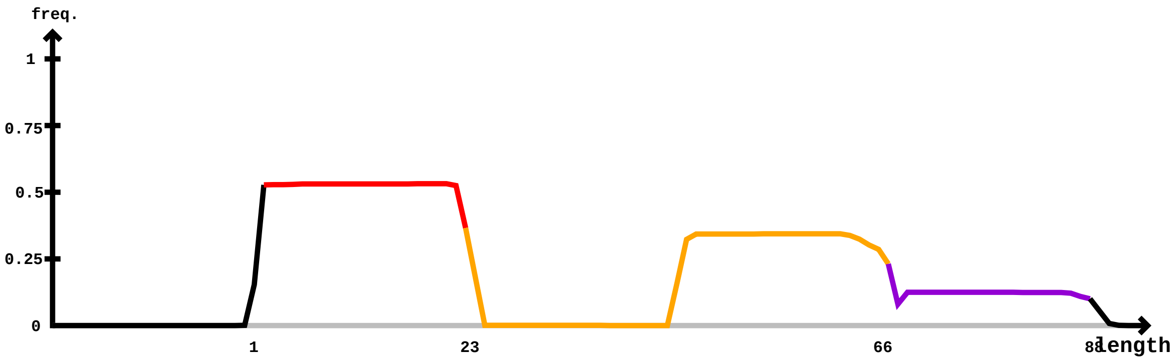

Mature Star

```
5'- gaaaacgaugcggccucgggugccaacgugauuugcaacuccgacugcgucacccaggaugugagccccucugugacgcagucggaguugcaaaucacguuggcagcgcgag   -3'   obs
    gaaaacgaugcggccucgggugccaacgugauuugcaacuccgacugcgucacccaggaugugagccccucugugacgcagucggaguugcaaaucacguuggcagcgcgag         exp
    ..............(((((.(((((((((((((((((((((((((((((((((..(((.........)))..))))))))))))))))))))))))))))))))).).))))    reads   mm   sample
    ..................ggugccaacgugauuugcaacu........................................................................    1       0    smb
    ...................gugccaacgugauuugcaacu........................................................................    3       0    smb
    ...................gugccaacgugauuugcaacAc.......................................................................    1       1    smb
    ...................gugccaacUugauuugcaacuc.......................................................................    1       1    smb
    ...................gugccaacgugauuugcaacuc.......................................................................    148     0    smb
    ...................gugccaacgugauuugcaacucA......................................................................    1       1    smb
    ...................gugccaacgugauuugcaacucU......................................................................    20      1    smb
    ...................Augccaacgugauuugcaacucc......................................................................    1       1    smb
    ....................ugccaacgugauuugcaacu........................................................................    4       0    smb
    ....................ugccaacgugauuugcaacuc.......................................................................    31      0    smb
    ....................ugccaacgugaCuugcaacucc......................................................................    1       1    smb
    ....................Cgccaacgugauuugcaacucc......................................................................    2       1    smb
    ....................ugccaacgugauuugcaacucA......................................................................    2       1    smb
    ....................ugccaacgugauuugcaacucc......................................................................    294     0    smb
    ....................ugccaacgugauuugcaacucU......................................................................    31      1    smb
    ....................ugccaacgugauuugcaacuccC.....................................................................    1       1    smb
    ....................ugccaacgugauuugcaacuccU.....................................................................    62      1    smb
    .....................gccaacgugauuugcaacucc......................................................................    1       0    smb
    .......................caacgugauuugcaacuc.......................................................................    1       0    smb
    ........................aacgugauuugcaacucc......................................................................    2       0    smb
    ....................................aacuccgacugcgucaccca........................................................    1       0    smb
    ...............................................................agccccucugugacgcag...............................    7       0    smb
    ...............................................................agccccucugugacgcagu..............................    16      0    smb
    ...............................................................agccccucugugacgcaguc.............................    23      0    smb
    ...............................................................agccccucugugacgcagucU............................    3       1    smb
    ...............................................................agccccucugugacgcagucg............................    13      0    smb
    ...............................................................agccccucugugacgcagucgU...........................    2       1    smb
    ...............................................................agccccucugugacgcagucgg...........................    102     0    smb
    ...............................................................agccccucugugacgcagucgga..........................    11      0    smb
    ...............................................................agccccucugugacgcagucggU..........................    3       1    smb
    ...............................................................agccccucugugacgcagucggaU.........................    1       1    smb
    ................................................................gccccucugugacgcaguc.............................    1       0    smb
    ................................................................gccccucugugacgcagucg............................    4       0    smb
```

Mature Star

gaaaacgaugcggccucgggugccaacgugauuugcaacuccgacugcgucacccaggaugugagccccucugugacgcagucggaguugcaaaucacguuggcagcgcgag			
....gccccucugugacgcagucgC...........................	1	1	smb
....gccccucugugacgcagucgU...........................	4	1	smb
....gccccucugugacgcagucgg...........................	7	0	smb
....gccccucugugacgcagucgga..........................	166	0	smb
....gccccucugugacgcagucggG..........................	2	1	smb
....gccccucuUugacgcagucgga..........................	2	1	smb
....gccccucugugacgcagucggU..........................	1	1	smb
....gccccucugugacgcagucggaU.........................	1	1	smb
....ccccucugugacgcaguU.............................	1	1	smb
....ccccucugugacgcagucgga..........................	1	0	smb
....ccccucugugacgcagucggag.........................	20	0	smb
....ccccucugugacCcagucggag.........................	1	1	smb
....gugacgcagucggaguugcaaaucacg.............	1	0	smb
....gaguugcaaaucacguuggcU.......	1	1	smb
....aguugcaaaucacguuggc........	3	0	smb
....aguugcaaaucacguuggcU.......	1	1	smb
....aguugcaaaucacguuggca.......	1	0	smb
....aguugcaaaucacguuggcag......	1	0	smb
....aguugcaaaucacguuggcagc.....	47	0	smb
....aguugcaaaucacgAuggcagc.....	1	1	smb
....guugcaaaucacguuggcU.......	2	1	smb
....guugcaaaucacguuggca.......	4	0	smb
....guugcaaaucacguuggcag......	4	0	smb
....guugcaaaucacguuggcaU......	1	1	smb
....guugcaaaucacguuggcagc.....	1	0	smb
....guugcaaaucacguuggcagcg....	1	0	smb
....uugcaaaucacguuggca.......	5	0	smb
....uugcaaaucacguuggcag......	3	0	smb
....uugcaaaucacguuggcaU......	1	1	smb
....uugcaaaucacguuggcagU.....	2	1	smb
....uugcaaaucacguuggcagc.....	41	0	smb
....uugcaaaucacguuggcagcU....	2	1	smb
....uugcaaaucacguuggcagcg....	11	0	smb
....uugcaaaucacguuggcagcgc...	8	0	smb
....uugcaaaucacguuggcagcgcg..	1	0	smb

Provisional ID	:	3820014_173156_5996552_3688891+,...,3767802-_15417
Score total	:	2373
Score for star read(s)	:	3.9
Score for read counts	:	2364.7
Score for mfe	:	3.4
Score for randfold	:	1.6
Score for cons. seed	:	-0.6
Total read count	:	4650
Mature read count	:	2562
Loop read count	:	2086
Star read count	:	2

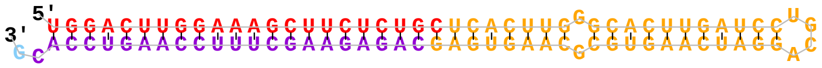

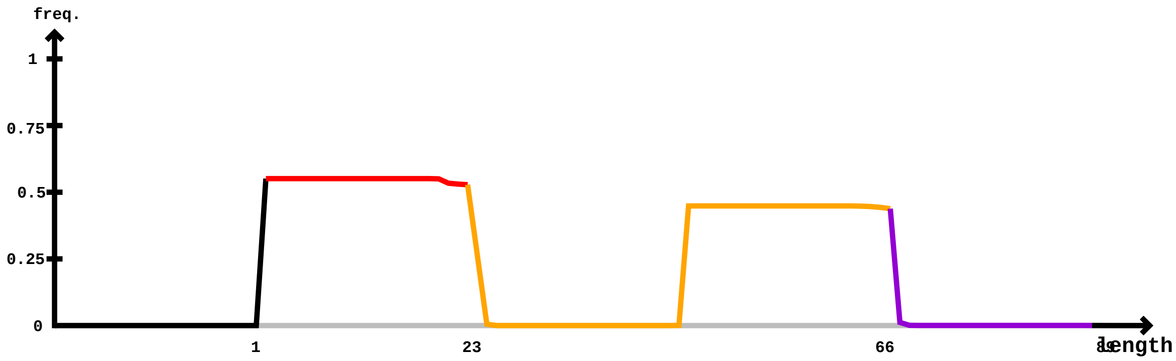


```
                  Mature                                                Star
5'- ggguugcagaugccgcuccguggacuuggaaagcuucucugcucacuugggcacuugauccugcaggaucaagugcgcaagugagcagagaagcuuuccaaguccacggagc  -3'   obs
    ggguugcagaugccgcuccguggacuuggaaagcuucucugcucacuugggcacuugauccugcaggaucaagugcgcaagugagcagagaagcuuuccaaguccacggagc        exp
    .(((.......)))(((((((((((((((((((((((((((((((((((.(((((((((((....))))))))))).)))))))))))))))))))))))))))))))))))   reads   mm   sample
    ....................uggacuuggaaagcuucu..........................................................................   4       0    smb
    ....................uggacuuggaaagcuucuc.........................................................................   75      0    smb
    ....................uggacuuggaaGgcuucuc.........................................................................   1       1    smb
    ....................uggacuuggaaagcCucucu........................................................................   1       1    smb
    ....................uggacuuggaaagcuucuUu........................................................................   1       1    smb
    ....................uggacuuggaaagcuucucu........................................................................   13      0    smb
    ....................uggacuuggaaagcuucucuU.......................................................................   1       1    smb
    ....................uggacuuggaaagcuucucug.......................................................................   10      0    smb
    ....................uggacuuggaaagcuucucuUc......................................................................   1       1    smb
    ....................uggacuuggaUagcuucucugc......................................................................   1       1    smb
    ....................uggacuuggaaagAuucucugc......................................................................   1       1    smb
    ....................uggacuuggaaagcuucucugU......................................................................   8       1    smb
    ....................uggacuuggaaagcuucucugc......................................................................   2352    0    smb
    ....................uggacuugCaaagcuucucugc......................................................................   1       1    smb
    ....................ugUacuuggaaagcuucucugc......................................................................   4       1    smb
    ....................uggacuAggaaagcuucucugc......................................................................   1       1    smb
    ....................uggacuuggaaagcuucCcugc......................................................................   2       1    smb
    ....................uggacuuggaaagcuucucCgc......................................................................   1       1    smb
    ....................uggacuuggaGagcuucucugc......................................................................   1       1    smb
    ....................uggacuuggGaagcuucucugc......................................................................   5       1    smb
    ....................uggacuuggaaagcCucucugc......................................................................   1       1    smb
    ....................uggacuuggaaagcuucucugcu.....................................................................   51      0    smb
    ....................uggacuuggaaagcuucucugUu.....................................................................   2       1    smb
    ....................uggacuuggaaagcuucucugcuU....................................................................   24      1    smb
    ...............................................................caggaucaagugcgcaagugagc..........................   3       0    smb
    ................................................................aggaucaagugcgcaagu..............................   4       0    smb
    ................................................................aggaucaagugcgcaaguU.............................   1       1    smb
    ................................................................aggaucaagugcgcaagug.............................   6       0    smb
    ................................................................aggaucaagugcgcaagugU............................   2       1    smb
    ................................................................aggaucaagugcgcaaguga............................   13      0    smb
    ................................................................aggaucaagugcgcaagugag...........................   20      0    smb
    ................................................................aggaucaagugcgcaagugaU...........................   3       1    smb
    ................................................................aggaucaaAugcgcaagugagc..........................   1       1    smb
```

```
                     Mature                                                   Star
ggguugcagaugccgcuccguggacuuggaaagcuucucugcucacuugggcacuugauccugcaggaucaagugcgcaagugagcagagaagcuuuccaaguccacggagc
................................................................aggaucaagugcgcUagugagc..........................   1      1    smb
................................................................aggaucaagugcgcaagugagc..........................   1960   0    smb
................................................................Gggaucaagugcgcaagugagc..........................   1      1    smb
................................................................aCgaucaagugcgcaagugagc..........................   1      1    smb
................................................................aggaucaagCgcgcaagugagc..........................   1      1    smb
................................................................aggaucaagugUgcaagugagc..........................   2      1    smb
................................................................aggaucaUgugcgcaagugagc..........................   1      1    smb
................................................................aggaucaagugcgcaagugagU..........................   4      1    smb
................................................................aggaucaagAgcgcaagugagc..........................   2      1    smb
................................................................aggaucaagugcAcaagugagc..........................   2      1    smb
................................................................aggaucaagugcgcaaguAagc..........................   1      1    smb
................................................................aggaucGagugcgcaagugagc..........................   1      1    smb
................................................................aggaucaagugcgcaagugagG..........................   1      1    smb
................................................................Uggaucaagugcgcaagugagc..........................   1      1    smb
................................................................aggaucaagugcgcaagugagcC.........................   2      1    smb
................................................................aggaucaagugcgcaagugagcU.........................   49     1    smb
................................................................aggaucaagugcgcaagugagcUg........................   1      1    smb
................................................................aggaucaagugcgcaagugagcaU........................   1      1    smb
.................................................................ggaucaagugcgcaagugagc..........................   1      0    smb
.....................................................................................cagagaagcuuuccaaguccac.....   1      0    smb
......................................................................................agagaagcuuuccaagucca......   1      0    smb
```

Provisional ID	:	3822730_183324_5706849_3728833+,...,3718809-_19405
Score total	:	295.3
Score for star read(s)	:	3.9
Score for read counts	:	287.2
Score for mfe	:	3.2
Score for randfold	:	1.6
Score for cons. seed	:	-0.6
Total read count	:	575
Mature read count	:	328
Loop read count	:	228
Star read count	:	19

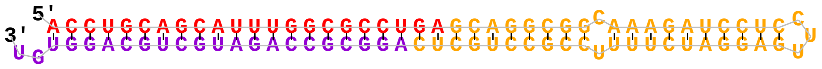

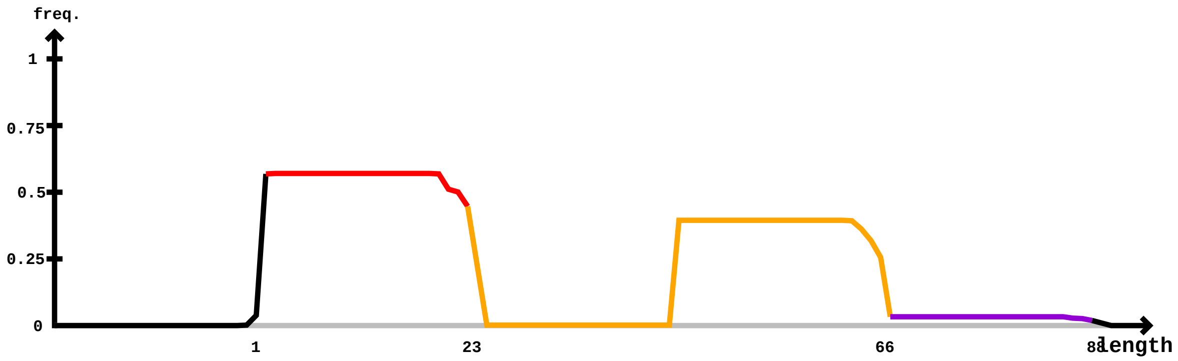

Mature **Star**

```
5'- caucauugacaacaugcuccaccugcagcauuuggcgccugagcaggcggcaaagauccuccuugaggaucuuuuccgccugcucaggcgccagaugcugcagguguggauu  -3'  obs
    caucauugacaacaugcuccaccugcagcauuuggcgccugagcaggcggcaaagauccuccuugaggaucuuuuccgccugcucaggcgccagaugcugcagguguggauu       exp
    ................(..(((((((((((((((((((((((((((((((.((((((((((...)))))))))).)))))))))))))))))))))))))))))))..)...   reads  mm  sample
    ..................cAaccugcagcauuuggcgccuga......................................................................   1      1   smb
    ...................caccugcagcauuuggcgcc.........................................................................   1      0   smb
    ...................caccugcagcauuuggcgccu........................................................................   2      0   smb
    ...................caccugcagcauuuggcgccug.......................................................................   18     0   smb
    ....................accugcagcauuuggcgc..........................................................................   1      0   smb
    ....................accugcagcauuuggcgcc.........................................................................   32     0   smb
    ....................accugcagcauuuggcgccu........................................................................   4      0   smb
    ....................accugcagcauuuggcgccGg.......................................................................   2      1   smb
    ....................accugcagcauuuggcgccug.......................................................................   11     0   smb
    ....................accugcagcaAuuggcgccuga......................................................................   1      1   smb
    ....................accugcagcauuuggcgccugU......................................................................   2      1   smb
    ....................accugcagcauuuggcgccuga......................................................................   251    0   smb
    ....................accugcagcauuuggcgUcuga......................................................................   1      1   smb
    .....................Acugcagcauuuggcgccuga......................................................................   1      1   smb
    ..........................................gcaggcggcaaagauccuccu.................................................   1      0   smb
    ...............................................................ugaggaucuuuuccgccu...............................   1      0   smb
    ...............................................................ugaggaucuuuuccgccug..............................   18     0   smb
    ...............................................................ugaggaucuuuuccgccugU.............................   3      1   smb
    ...............................................................ugaggaucuuuuccgccugc.............................   22     0   smb
    ...............................................................ugaggaucuuuuccgccugcu............................   35     0   smb
    ...............................................................ugaggaucCuuuccgccugcu............................   1      1   smb
    ...............................................................ugaggaucuuuCccgccugcuc...........................   1      1   smb
    ...............................................................ugaggaucuuuuccgcUugcuc...........................   1      1   smb
    ...............................................................ugaggaucuuuuccgccugcCc...........................   1      1   smb
    ...............................................................ugaggaucuuuuccgccugcuc...........................   144    0   smb
    .....................................................................................aggcgccagaugcugcagg........   3      0   smb
    .....................................................................................aggcgccagaugcugcaggu.......   1      0   smb
    .....................................................................................aggcgccagaugcugcaggug......   4      0   smb
    .....................................................................................aggcgccagaugcugcaggugu.....   9      0   smb
    .....................................................................................aggcgccagaugcugcagguguA....   2      1   smb
```

```
Provisional ID              : 3751232_4773_84610_4186_145921_100637_404_521785_129649_7726
Score total                 :     623.1
Score for star read(s)      :       3.9
Score for read counts       :       615
Score for mfe               :       3.2
Score for randfold          :       1.6
Score for cons. seed        :      -0.6
Total read count            :      1218
Mature read count           :       970
Loop read count             :       198
Star read count             :        50
```

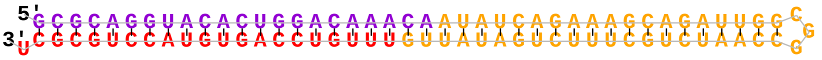

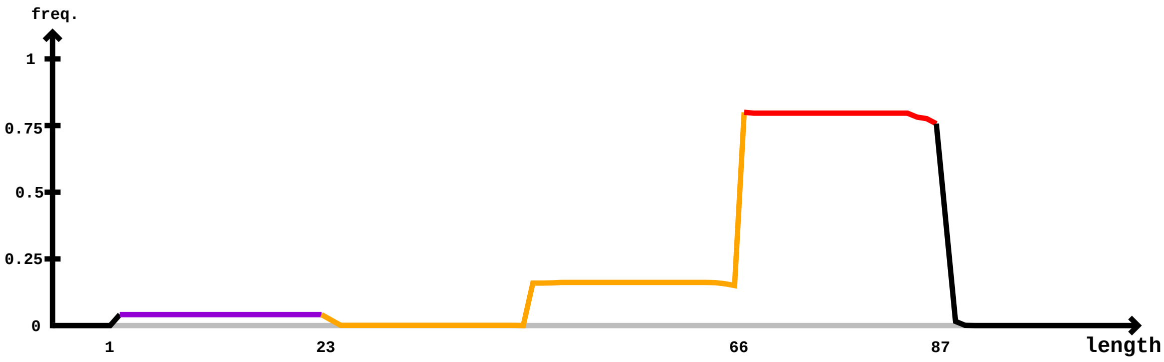


```
         Star                                      Mature
5'- auccagcgcagguacacuggacaaacaauaucagaaagcagauuggcggccaaucugcuuucugauauuguuuguccaguguaccugcgcuggaggggaggggccgcuugc -3'  obs
    auccagcgcagguacacuggacaaacaauaucagaaagcagauuggcggccaaucugcuuucugauauuguuuguccaguguaccugcgcuggaggggaggggccgcuugc       exp
    .(((((((((((((((((((((((((((((((((((((((((((((...)))))))))))))))))))))))))))))))))))))))))))))(.((.....)).)....  reads  mm  sample
    .....gcgcagguacacuggacaaaca....................................................................................  49     0   smb
    .....gcgcagguacacuggacaaacaU...................................................................................  1      1   smb
    ...........................auaucagaaagcagauuggc................................................................  1      0   smb
    ................................................gccaaucugcuuucugaua............................................  1      0   smb
    ................................................gccaaucugcuuucugauau...........................................  5      0   smb
    ................................................gccaaucugcuuucugauauu..........................................  9      0   smb
    ................................................gccaaucugcuuucugauauug.........................................  174    0   smb
    ................................................gccaaucugcuuucugauauuU.........................................  1      1   smb
    ................................................gccaaucugcuuucugauauugu........................................  4      0   smb
    ..................................................caaucugcuuucugauauug.........................................  1      0   smb
    ...................................................aaucugcuuucugauauug.........................................  2      0   smb
    .....................................................................guuuguccaguguaccugcgcu....................  1      0   smb
    ......................................................................uuuguccaguguaccugU.......................  8      1   smb
    ......................................................................uuuguccaguguaccugc.......................  10     0   smb
    ......................................................................uuuguccaguguaccugcg......................  2      0   smb
    ......................................................................uuuguccaguguaccugcU......................  4      1   smb
    ......................................................................uuuguccaguguaccugcC......................  1      1   smb
    ......................................................................uuuguccaguguaccugcgc.....................  22     0   smb
    ......................................................................uuuguccaguguaccugcgU.....................  1      1   smb
    ......................................................................uuuguccaguguaccugcgcC....................  1      1   smb
    ......................................................................uuuguccaguguaccuCcgcu....................  2      1   smb
    ......................................................................uuCguccaguguaccugcgcu....................  1      1   smb
    ......................................................................uuugCccaguguaccugcgcu....................  1      1   smb
    ......................................................................Cuuguccaguguaccugcgcu....................  1      1   smb
    ......................................................................uuuguccagugAaccugcgcu....................  2      1   smb
    ......................................................................uuuguccaguguaccCgcgcu....................  1      1   smb
    ......................................................................uuuguccaguguaccugcgUu....................  2      1   smb
    ......................................................................uuuguccaguguaccugcgcu....................  693    0   smb
    ......................................................................uuuguccaguguaccugcgcG....................  2      1   smb
    ......................................................................uuuguccaguguaUcugcgcu....................  1      1   smb
    ......................................................................uuuguccaguguacUugcgcu....................  1      1   smb
    ......................................................................uuuguccagugAaccugcgcug...................  1      1   smb
    ......................................................................uuuguccaUuguaccugcgcug...................  1      1   smb
```

```
     Star                                                                Mature
auccagcgcagguacacuggacaaacaauaucagaaagcagauuggcggccaaucugcuuucugauauuguuuguccaguguaccugcgcuggaggggaggggccgcuugc
......................................................................uuuguccaguguaccugcCcug...................   1     1     smb
......................................................................uuuguccaguguaccugcgcug...................   191   0     smb
......................................................................uuuguccaguguaccugcgcugU..................   18    1     smb
......................................................................uuuguccaguguaccugcgcugUa.................   1     1     smb
```

Provisional ID : 3820154_60939_1893288_3690600+,...,3803400+_15625
Score total : 433.2
Score for star read(s) : 3.9
Score for read counts : 424.8
Score for mfe : 3.5
Score for randfold : 1.6
Score for cons. seed : -0.6
Total read count : 845
Mature read count : 534
Loop read count : 0
Star read count : 311

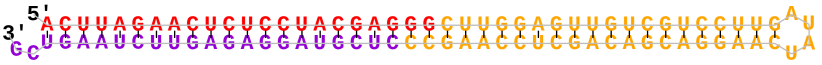

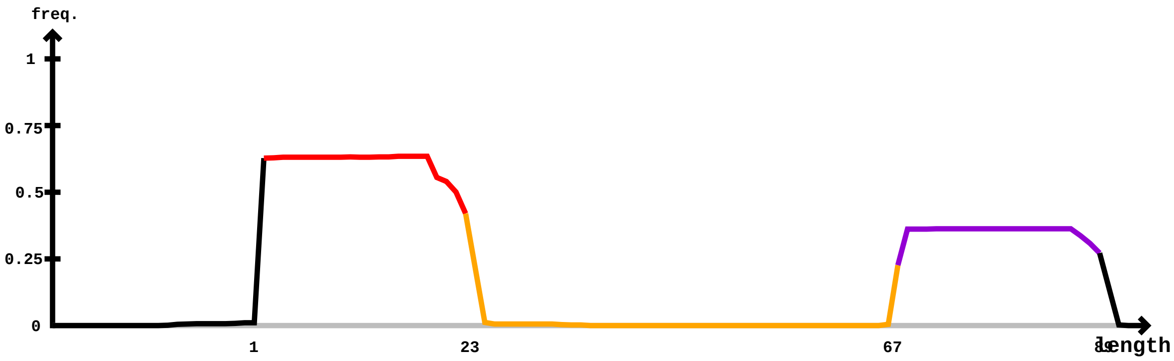

Mature Star

```
5'- agccugacugaucauggacgacuuagaacucuccuacgagggcuuggaguugucguccuugauaucaaggacgacagcuccaagcccucguaggagaguucuaagucgucca  -3'  obs
    agccugacugaucauggacgacuuagaacucuccuacgagggcuuggaguugucguccuugauaucaaggacgacagcuccaagcccucguaggagaguucuaagucgucca        exp
    ..............(((((((((((((((((((((((((((((((((((((((((((((((....)))))))))))))))))))))))))))))))))))))))))))))))  reads  mm  sample
    ..........aucauggacgacuuagaacucuccuacgaggg......................................................................  1  0  smb
    ...........ucauggacgacuuagaacu..................................................................................  1  0  smb
    ...........ucauggacgacuuagaacucuccuacgagggc.....................................................................  2  0  smb
    ............cauggacgacuuagaacucuccuacgagggcu....................................................................  1  0  smb
    .............auggacgacuuagaacucuccu.............................................................................  1  0  smb
    .................acgacuuagaacucuccuacgagggcu....................................................................  1  0  smb
    ..................cgacuuagaacuGuccuacgagggcu....................................................................  2  1  smb
    ....................acuuagaacucuccuacg..........................................................................  66  0  smb
    ....................acuuagaaUucuccuacg..........................................................................  1  1  smb
    ....................acuuagaacucuccuacU..........................................................................  1  1  smb
    ....................acuuagaacucuccuacgU.........................................................................  2  1  smb
    ....................acuuagaacucuccuacga.........................................................................  10  0  smb
    ....................acuuagaacucuccuacgaU........................................................................  1  1  smb
    ....................acuuagaacucuccuacgag........................................................................  33  0  smb
    ....................acuuagaacucuccuacgagU.......................................................................  1  1  smb
    ....................acuuGgaacucuccuacgagg.......................................................................  1  1  smb
    ....................acuuagaacucuccuacgagg.......................................................................  67  0  smb
    ....................acuuagaaUucuccuacgaggg......................................................................  1  1  smb
    ....................acuuagaacucuccuacgaggU......................................................................  2  1  smb
    ....................acuuagaacucuccuacgaggg......................................................................  333  0  smb
    ....................acuuagaacucuUcuacgaggg......................................................................  1  1  smb
    ....................acuuagaacucuccuacgagggU.....................................................................  8  1  smb
    ....................acuuagaacucuccuacgagggA.....................................................................  1  1  smb
    .....................cuuagaacucuccuacga.........................................................................  1  0  smb
    ......................uuagaacuGuccuacgaggg......................................................................  1  1  smb
    ......................uuagaacuGuccuacgagggcu....................................................................  1  1  smb
    .............................ucuccuacgagggcuuggaguu.............................................................  1  0  smb
    ................................ccuacgagggcuuggaguu.............................................................  1  0  smb
    ..................................uacgagggcuuggaguug............................................................  1  0  smb
    ..................................uacgagggcuuggaguuguc..........................................................  1  0  smb
    ...................................acgagggcuuAgaguuguc..........................................................  1  1  smb
    .....................................................................................ccucguaggagaguucuaag.......  1  0  smb
    .....................................................................................ccucguaggagaguucuaaguc.....  2  0  smb
```

Mature **Star**

```
agccugacugaucauggacgacuuagaacucuccuacgagggcuuggaguugucguccuugauaucaaggacgacagcuccaagcccucguaggagaguucuaagucgucca
.....................................................................................Ucucguaggagaguucuaagucg....	1	1	smb
......................................................................................cucguaggagaguucuaaU.......	2	1	smb
......................................................................................cucguaggagaguucuaag.......	19	0	smb
......................................................................................cucguaggagaguucuaagu......	24	0	smb
......................................................................................cucguaggagaguucuaaguc.....	29	0	smb
......................................................................................cucguaggagaguucuaagucg....	107	0	smb
......................................................................................cucguaggagaguucuaagucU....	2	1	smb
......................................................................................cucguaggagaguucuaagucgu...	7	0	smb
.......................................................................................ucguaggagaguucuaagu......	1	0	smb
.......................................................................................ucguaggagaguucuaagucU....	1	1	smb
.......................................................................................ucguaggagaguucuaagucg....	5	0	smb
.......................................................................................ucguaggagaguucuaagucgu...	107	0	smb
.......................................................................................ucguaggagaguucuaagucgG...	1	1	smb
.......................................................................................ucguaggagaguucuaagucguU..	1	1	smb
..........................................................................................uaggagaguucuaagucguc..	1	0	smb
```

Provisional ID	:	3821282_105958_2860554_3706534-,...,3810739+_17209
Score total	:	532
Score for star read(s)	:	3.9
Score for read counts	:	523.7
Score for mfe	:	3.4
Score for randfold	:	1.6
Score for cons. seed	:	-0.6
Total read count	:	1039
Mature read count	:	499
Loop read count	:	0
Star read count	:	540

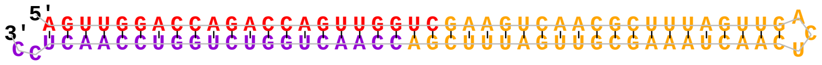

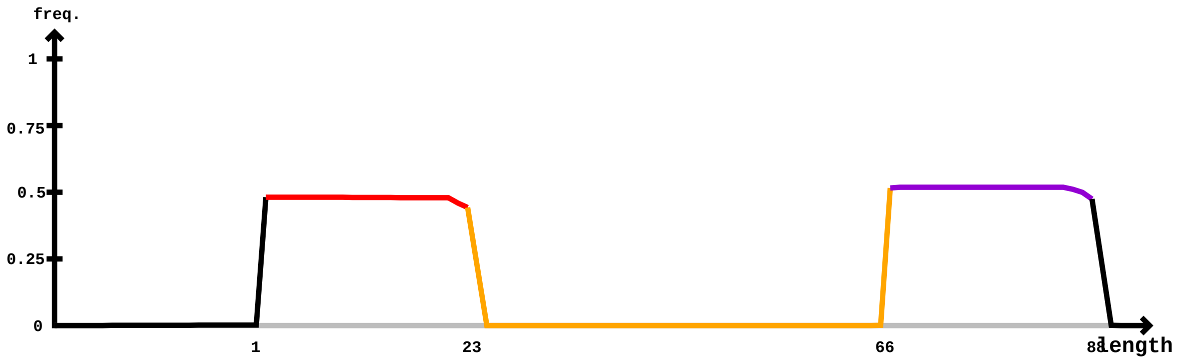

Mature **Star**

```
5'- uaauuucacaucuuuggcggaguuggaccagaccaguuggucgaagucaacgcuuuaguugacucaacuaaagcguugauuucgaccaacuggucugguccaacuccgccaa  -3'   obs
    uaauuucacaucuuuggcggaguuggaccagaccaguuggucgaagucaacgcuuuaguugacucaacuaaagcguugauuucgaccaacuggucugguccaacuccgccaa        exp
    .............((((((((((((((((((((((((((((((((((((((((((((((((...))))))))))))))))))))))))))))))))))))))))))))))))   reads   mm   sample
    ....uucacaucuuuggcggaguuggacc...................................................................................   1       0    smb
    .............uuggcggaguuggaccagacc..............................................................................   1       0    smb
    ....................aguuggaccagaccaguugg........................................................................   21      0    smb
    ....................aguuggaccagaccaguuggu.......................................................................   17      0    smb
    ....................aguuggaccagaccaguugguc......................................................................   451     0    smb
    ....................aguuggaccagaccUguugguc......................................................................   1       1    smb
    ....................aguuggaccagaccaguugguU......................................................................   3       1    smb
    ....................Gguuggaccagaccaguugguc......................................................................   1       1    smb
    ....................aguuggaccagaccaguuggucU.....................................................................   5       1    smb
    ....................................................................................accaacuggucugguccaacuc......   1       0    smb
    .....................................................................................ccaacuggucugguccaaU........   1       1    smb
    .....................................................................................ccaacuggucugguccaac........   7       0    smb
    .....................................................................................ccaacuggucugguccaacu.......   12      0    smb
    .....................................................................................ccaacuggucugguccaacuc......   25      0    smb
    .....................................................................................ccGacuggucugguccaacucc.....   1       1    smb
    .....................................................................................Acaacuggucugguccaacucc.....   1       1    smb
    .....................................................................................ccaacuggucugguccaacucc.....   432     0    smb
    .....................................................................................ccaacuggucugguccaacucU.....   10      1    smb
    .....................................................................................ccaacuggucugguccaacuccU....   47      1    smb
    ......................................................................................caacuggucugguccaacucc.....   2       0    smb
    ......................................................................................caacuggucugguccaacuccgU...   1       1    smb
```

Provisional ID	:	3826458_5601_156149_3783984-,569N,3805326-_24732
Score total	:	188.7
Score for star read(s)	:	3.9
Score for read counts	:	181.1
Score for mfe	:	2.7
Score for randfold	:	1.6
Score for cons. seed	:	-0.6
Total read count	:	367
Mature read count	:	233
Loop read count	:	127
Star read count	:	7

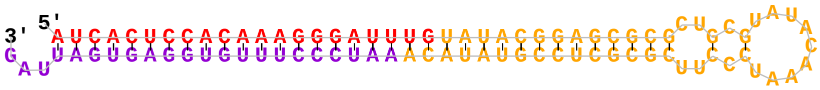

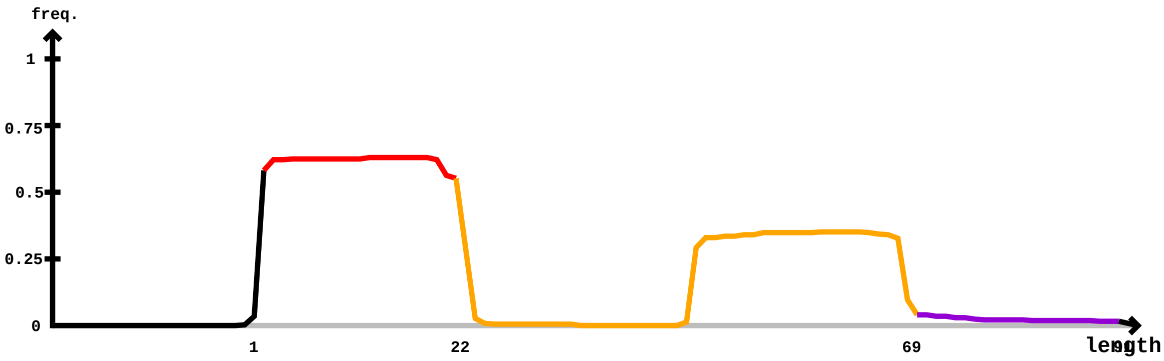

Mature Star

```
5'- cugcacaaaugcacaaaccaaucacuccacaaagggauuuguauacggagcgcgcugcguauacaaaucccuucgcgcuccguauacaaaucccuuuguggagugauuagu -3'    obs
    cugcacaaaugcacaaaccaaucacuccacaaagggauuuguauacggagcgcgcugcguauacaaaucccuucgcgcuccguauacaaaucccuuuguggagugauuagu         exp
    .((((....))))...((.(((((((((((((((((((((((((((((((((((((..(.(.........).)..))))))))))))))))))))))))))))))))))))).))   reads   mm   sample
    ..................caaucacuccacaaagggau.........................................................................   1   0   smb
    ...................aaucacuccacaaagggauuug......................................................................   11   0   smb
    ...................aaucacuccacaaagggauuugu.....................................................................   1   0   smb
    ....................aucacuccacaaagggau.........................................................................   2   0   smb
    ....................aucacuccacaaagggauu........................................................................   21   0   smb
    ....................aucacuccacaaagggauuu.......................................................................   4   0   smb
    ....................aucacuccacaaagggauuug......................................................................   135   0   smb
    ....................aucacuccacaaaUggauuug......................................................................   1   1   smb
    ....................aucacuccacaaagggauuugu.....................................................................   40   0   smb
    ....................aucacuccacaaagggauuuguU....................................................................   1   1   smb
    .....................ucacuccacaaagggauu........................................................................   1   0   smb
    .....................ucacuccacaaagggauuug......................................................................   7   0   smb
    .....................ucacuccacaaagggauuugua....................................................................   6   0   smb
    .....................ucacuccacaaagggauuuguau...................................................................   1   0   smb
    .......................acuccacaaagggauuuU......................................................................   1   1   smb
    ...............................aagggauuuguauacggagcgc..........................................................   2   0   smb
    ................................................................aaaucccuucgcgcuccguaua.........................   4   0   smb
    ................................................................aaaucccuucgcgcuccguauac........................   1   0   smb
    .................................................................aaucccuucgcgcuccgu............................   1   0   smb
    .................................................................aaucccuucgcgcuccgua...........................   2   0   smb
    .................................................................aaucccuucgcgcuccguau..........................   1   0   smb
    .................................................................aaucccuucgcgcuccguaua.........................   1   0   smb
    .................................................................aaucccuucgcgcuccguauac........................   85   0   smb
    .................................................................aaucccuucgcgcuccguauaU.........................   1   1   smb
    .................................................................aaucccuucgcgcuccguauacU.......................   12   1   smb
    .................................................................aaucccuucgcgcuccguauaca.......................   1   0   smb
    ..................................................................aucccuucgcgcuccguauaca.......................   14   0   smb
    ....................................................................cccuucgcgcuccguauacaaa.....................   2   0   smb
    ......................................................................cuucgcgcuccguauacaaauc...................   2   0   smb
    ........................................................................ucgcgcuccguauacaaauccc.................   2   0   smb
    ........................................................................ucgcgcuccguauacaaaucccu................   1   0   smb
    ..............................................................................uccguauacaaaucccuuugug...........   1   0   smb
    .......................................................................................aaaucccuuuguggagugau....   1   0   smb
```

```
                    Mature                                                                                 Star
cugcacaaaugcacaaaccaaucacuccacaaagggauuuguauacggagcgcgcugcguauacaaaucccuucgcgcuccguauacaaaucccuuuguggagugauuagu
........................................................................................aaucccuuuguggagugauuag.    4    0    smb
........................................................................................aaucccuuuguggagugauuagu    2    0    smb
```
