# Supplementary figures and images for: Integrating microRNA and mRNA expression profiling in Symbiodinium microadriaticum, a dinoflagellate symbiont of reef-building corals
Source: BMC Genomics. 2013 Oct 12;14:704. doi: 10.1186/1471-2164-14-704 (PMC3853145; doi:10.1186/1471-2164-14-704)

## RNase III 1

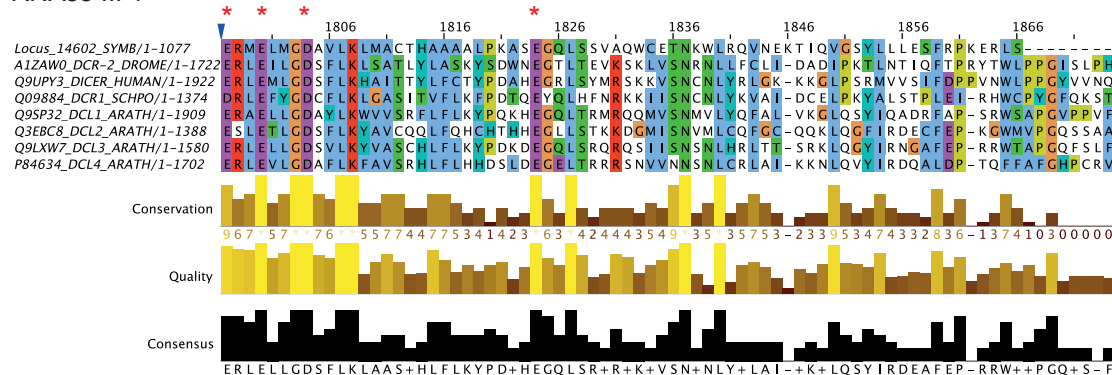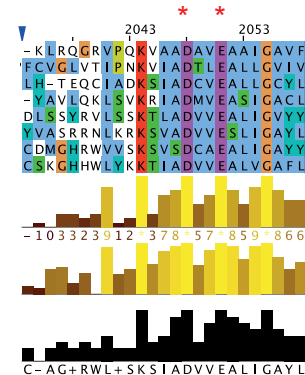

## RNase III 2

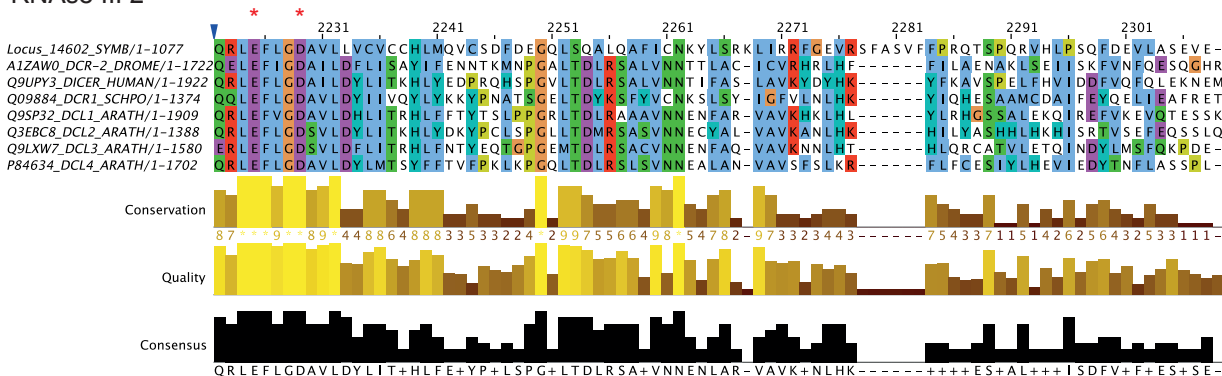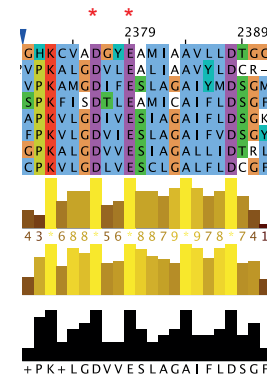

Supplement: Additional file 8 — Alignment of functional domains of the Symbiodinium microadriaticum homolog of the endoribonuclease Dicer with homologs from model organisms (S. pombe, A. thaliana, C. elegans, D. melanogaster, H. sapiens). Key functional residues are depicted with red asterisks. [file 1471-2164-14-704-S8.pdf]

## PAZ

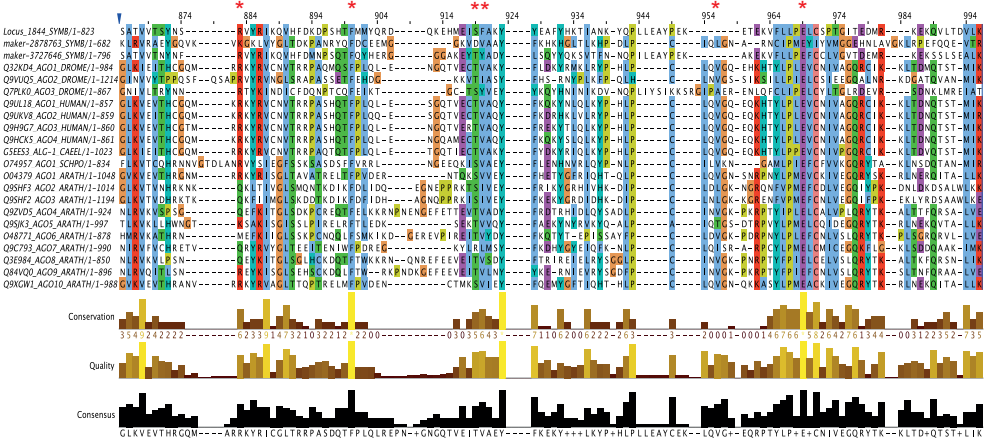

## PIWI

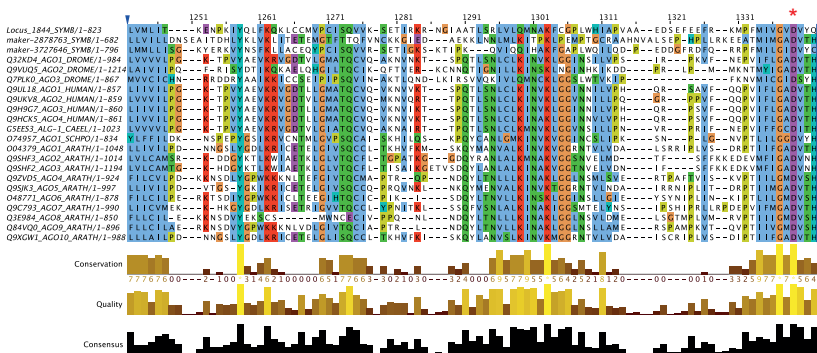

## PIWI

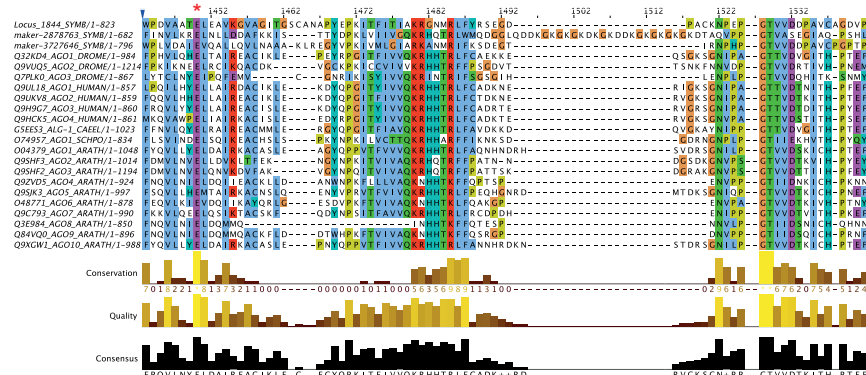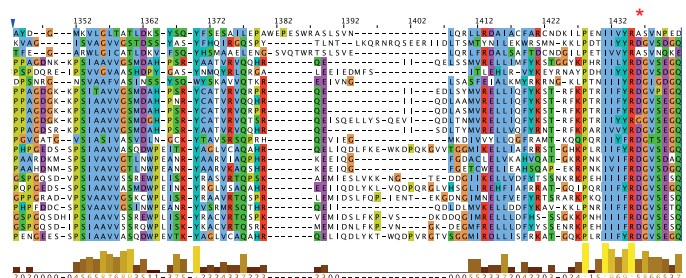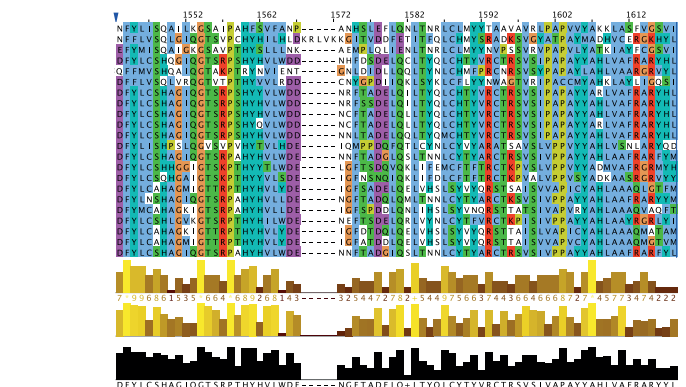

Supplement: Additional file 9 — Alignment of functional domains of Symbiodinium microadriaticum homologs of the Argonaute effector protein with homologs from model organisms (S. pombe, A. thaliana, C. elegans, D. melanogaster, H. sapiens). Key functional residues are depicted with red asterisks. [file 1471-2164-14-704-S9.pdf]

# Methyltransferase domain

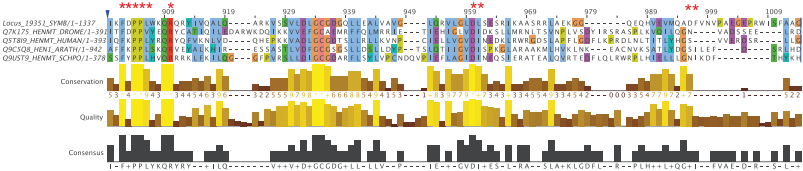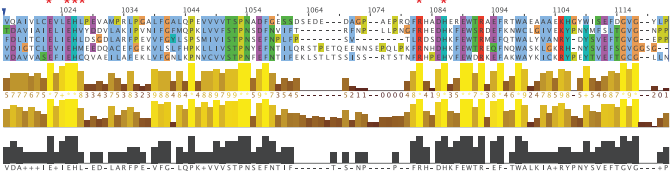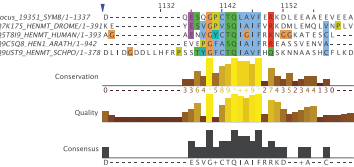

Supplement: Additional file 10 — Alignment of functional domains of the Symbiodinium microadriaticum homolog of the small RNA 2'-O-methyltransferase (HEN1) with homologs from model organisms (S. pombe, A. thaliana, C. elegans, D. melanogaster, H. sapiens). Key functional residues are depicted with red asterisks. [file 1471-2164-14-704-S10.pdf]
